# The transcriptomic profile of *Spodoptera frugiperda* differs in response to a novel insecticide, cyproflanilide, compared to chlorantraniliprole and avermectin

**DOI:** 10.1186/s12864-022-09095-2

**Published:** 2023-01-03

**Authors:** Haijuan Shu, Yufeng Lin, Zhengbing Zhang, Lin Qiu, Wenbing Ding, Qiao Gao, Jin Xue, Youzhi Li, Hualiang He

**Affiliations:** 1 Hunan Provincial Key Laboratory for Biology and Control of Plant Diseases and Insect Pests, College of Plant Protection, Hunan Agricultural University, Changsha, 410128 China; 2 Agriculture and Rural Department of Hunan Province, Plant Protection and Inspection Station, Changsha, 410005 China; 3 National Research Center of Engineering & Technology for Utilization of Botanical Functional Ingredients, Hunan Agricultural University, Changsha, 410128 China

**Keywords:** Cyproflanilide, *Spodoptera frugiperda*, Transcriptomic analyses, GABAR, P450

## Abstract

**Background:**

Cyproflanilide is a novel chemical that is already undergoing insecticide registration in China and has been categorized as a member of group 30 by the IRAC. Since it was first detected in 2019, the fall armyworm (FAW), *Spodoptera frugiperda*, has become a serious pest in China. Our laboratory and field efficacy trials indicated that cyproflanilide exhibits high larvicidal activity against FAW. However, the effect of cyproflanilide against FAW remains unknown. And it is worth exploring further before the cyproflanilide becomes commercially available.

**Results:**

We found larvae exposed to cyproflanilide had significantly shorter body length and higher death rates compared to control larvae. Additionally, we found surviving larvae had a significantly longer developmental period compared to control larvae. The potential molecular mechanisms of cyproflanilide against FAW were investigated using comparative transcriptomic analyses on larval samples subjected to three insecticide treatments, including cyproflanilide and two other commonly used insecticides against FAW in China, chlorantraniliprole and avermectin. We found that several subunits of the γ-aminobutyric acid receptor (GABAR), a possible target protein of cyproflanilide, were significantly up-regulated at the transcriptional level during cyproflanilide-induced stress. Additionally, between the control and cyproflanilide-treated samples, we identified 131 differentially expressed genes (DEGs) associated with detoxification metabolism. Of these, we found four P450 genes that were significantly up-regulated under cyproflanilide stress but were not DEGs when exposed to chlorantraniliprole and avermectin, or 23 other pesticides from previous reports. Furthermore, we discovered an interesting gene aggregation region for insect cuticle proteins (CPs) on the 18^th^ chromosome, which is likely related to FAW cross-resistance to cyproflanilide and avermectin.

**Conclusions:**

Our results contribute to a greater understanding of the mechanisms by which cyproflanilide affects FAW. Additionally, we identified the similarities and differences in transcriptomic profiling of FAW between the novel insecticide cyproflanilide and two other commonly used insecticides.

**Supplementary Information:**

The online version contains supplementary material available at 10.1186/s12864-022-09095-2.

## Background

Cyproflanilide is a novel, environmentally safe chemical, developed in recent years by CAC Nantong Taihe Chemical Co, Ltd (China) [1, 2]. In June 2020, the International Organization for Standardization (ISO) Technical Committee on Pesticide Nomenclature approved the use of cyproflanilide. Cyproflanilide exhibits high activity against a variety of pests, including lepidopteran, coleopteran, and thysanopteran pests, and to date has not shown cross-resistance with any existing insecticides [1, 2]. Cyproflanilide is a meta-diamide compound which was developed with biamide insecticide as the lead compound. It is a potent antagonist at the γ-aminobutyric acid receptor (GABAR) and has been categorized as a member (estimated to be the fourth member) of group 30 by the Insecticide Resistance Action Committee (IRAC). Cyproflanilide has a similar chemical structure to broflanilide, the first member of group 30 [3]. Broflanilide has been shown to metabolize to desmethyl-broflanilide. The site of action for desmethyl-broflanilide is thought to be close to G336 in the M3 region of the Drosophila RDL GABA receptor [4]. The insecticidal mechanisms of cyproflanilide and whether its metabolism resembles that of desmethyl-broflanilide remain unknown.

Since it was first detected in 2019, the fall armyworm (FAW), *Spodoptera frugiperda*, has become serious pest in China [5]. Many insecticides have been reported to control FAW larvae. The transcriptomic profiles of FAW have been analyzed following exposure to a variety of insecticides including avermectin [6], lambda-cyhalothrin [7, 8], azadirachtin [9], chlorantraniliprole, and other diamide insecticides [10], lufenuron [11], organophosphate and pyrethroid [12], harmine [13] or other plant allelochemicals [14], and the toxin produced by Bacillus thuringiensis (Bt) [15]. Studies on four species, including FAW, found that the cytochrome P450 monooxygenases (P450s; encoded by CYP genes) were the most upregulated detoxification genes in response to xenobiotics [16]. In our laboratory and field efficacy trial, we examined changes in the weight of FAW larvae fed an artificial diet treated with different cyproflanilide concentrations and found that cyproflanilide exhibits high larvicidal activity against FAW.

The aim of this study was to compare the different transcriptomic responses of FAW to cyproflanilide along with two other commonly used insecticides, chlorantraniliprole and avermectin. Chlorantraniliprole, is a type of amide compound that has some chemical similarities to cyproflanilide and avermectin is thought to cause tremor/convulsion through the GABA-ergic action with hyperpolarization of nerve/muscle cells [17]. Avermectin and cyproflanilide are both involved in GABA-ergic action. Our results reveal the similarities and differences in transcriptomic profiling of FAW between cyproflanilide, chlorantraniliprole, and avermectin. This research also contributes to a greater understanding of the mechanisms by which cyproflanilide affects FAW.

## Results

### Toxicity of cyproflanilide against *S. frugiperda* larval

**Fig. 1:**
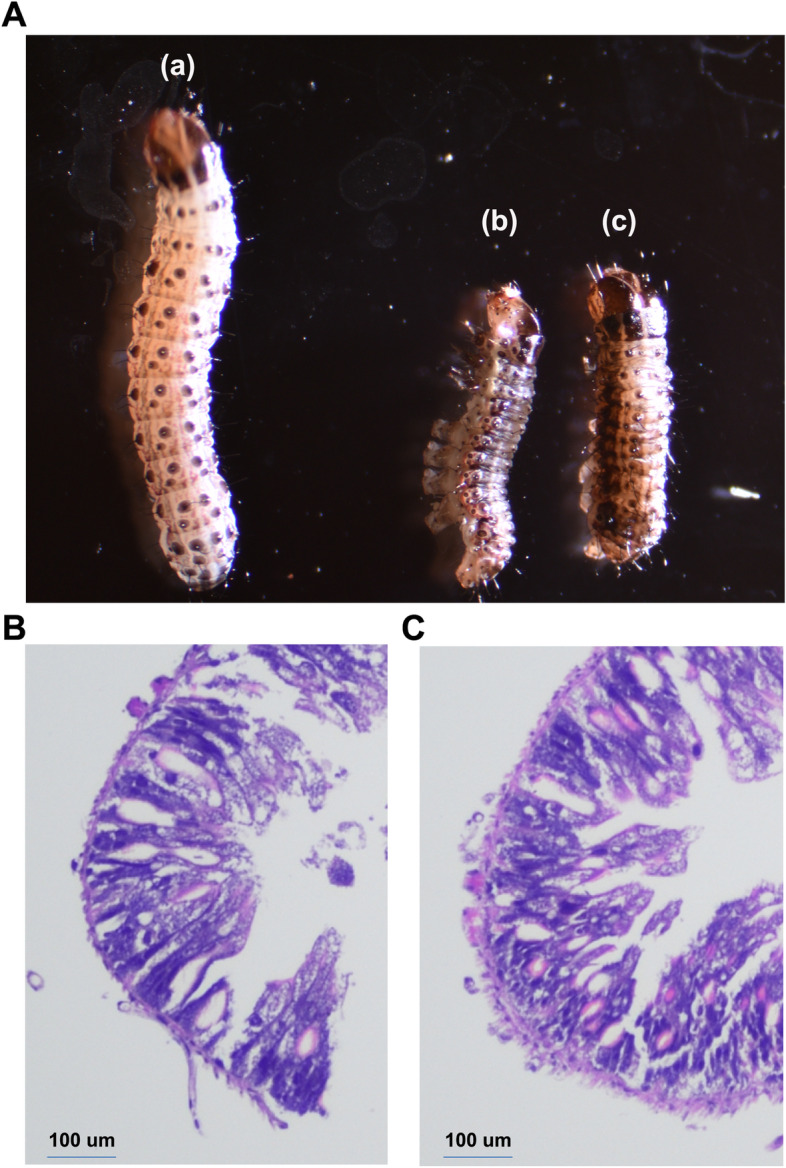
Larval poisoning state under stress of cyproflanilide and histopathological structural change analysis of the midgut. **A** The poisonous or lethal effect of cyproflanilide on third stage larvae of *Spodoptera frugiperda*. **a** Control larvae were fed an artificial diet supplemented with acetone. **b** Continuously writhing body of larva fed an artificial diet supplemented with acetone and cyproflanilide (LC_50_). **c** Dead larva fed on artificial diet supplemented with acetone and cyproflanilide (LC_50_). **B** Hematoxylin–eosin staining of the midgut dissected from larvae in the control fed an artificial diet supplemented with acetone. **C** Histopathological changes in the midgut dissected from larvae fed a diet supplemented with cyproflanilide (LC_50_)

We used probit regression analysis to calculate the different 24 h lethal concentrations (LC) of cyproflanilide (Supplement Fig. 1). To examine the toxicity of cyproflanilide against FAW, third-instar larvae were initially fed artificial diets containing sub-lethal concentrations (LC_10_, 0.15 μg/g) and (LC_30_, 0.21 μg/g) of cyproflanilide. The developmental duration of larvae for each stage, pupal weight, emergence, and the number of eggs per female were recorded following treatment. We found that larvae treated with cyproflanilide had a significantly longer developmental time compared to the control group (Supplement Table 1). However, there was no significant difference in pupal weight or the number of eggs per female between the cyproflanilide and control groups (Supplement Table 2). Third-instar larvae were then fed an artificial diet containing a median lethal concentration (LC_50_, 0.27 μg/g) of cyproflanilide. We found the body length of dead larvae was significantly shorter compare to control larvae (Fig. 1A, Supplement Table 3). Hematoxylin–eosin (HE) staining of midgut sections from larvae fed for 48 h on a diet containing cyproflanilide revealed only minor histopathological changes with midgut cells arranged tightly in multiple layers with a thick intestinal wall in control larvae (Fig. 1B), and a similar tight multiple layer arrangement of the midgut cells in larvae fed the cyproflanilide diet (Fig. 1C).

**Table 1:** Profile of DEGs on P450 superfamily, CPs and neurotransmitter receptors

Gene or family	Treatments							Total DEGs	Total unigenes
	**Cy**	**Av**	**Cl**	**Cy + Av**	**Cy + Cl**	**Av + Cl**	**Cy + Av + Cl**		
**P450**	14	22	13	14	19	9	3	93	173
**CP**	3	15	69	40	2	3	8	140	252
**GABAR**	0	0	0	0	1^ m^ + 2^i^	0	1^ m^ + 1^i^	2^ m^ + 3^i^	4^ m^ + 8^i^
**GluCl**	1	0	0	0	0	0	1	2	3
**RyR**	0	0	0	0	0	0	0	0	2

*Cy* Cyproflanilide, *Av* Avermectin, *Cl* Chlorantraniliprole, *m* Metabotropic GABAR, *I* Ionotropic GABAR. + , indicates that it is listed as DEG under two different treatments, or among three different treatments

### Transcriptomic analyses and functional annotation of differentially expressed genes (DEGs)

Larvae samples treated with cyproflanilide (LC_50_) for 24 h and control samples were used for transcriptomic analyses. Additionally, we dissected midgut samples from other larvae treated with cyproflanilide (LC_50_) for 24 h as well as larvae from the control group. The number of raw reads from libraries ranged from 40,221,888 to 53,396,952. High quality reads ranged from 38,054,356 to 52,454,226 (Supplement Table 4). The assembly of all combined reads yielded a total of 21,525 unigenes, including 695 novel unigenes. The unigenes included 951 lncRNA, 103 rRNA, 638 tRNA, 106 snRNA, 35 snoRNA, 153 transcribed_pseudogene, and 18,811 protein_coding unignenes.

**Fig. 2:**
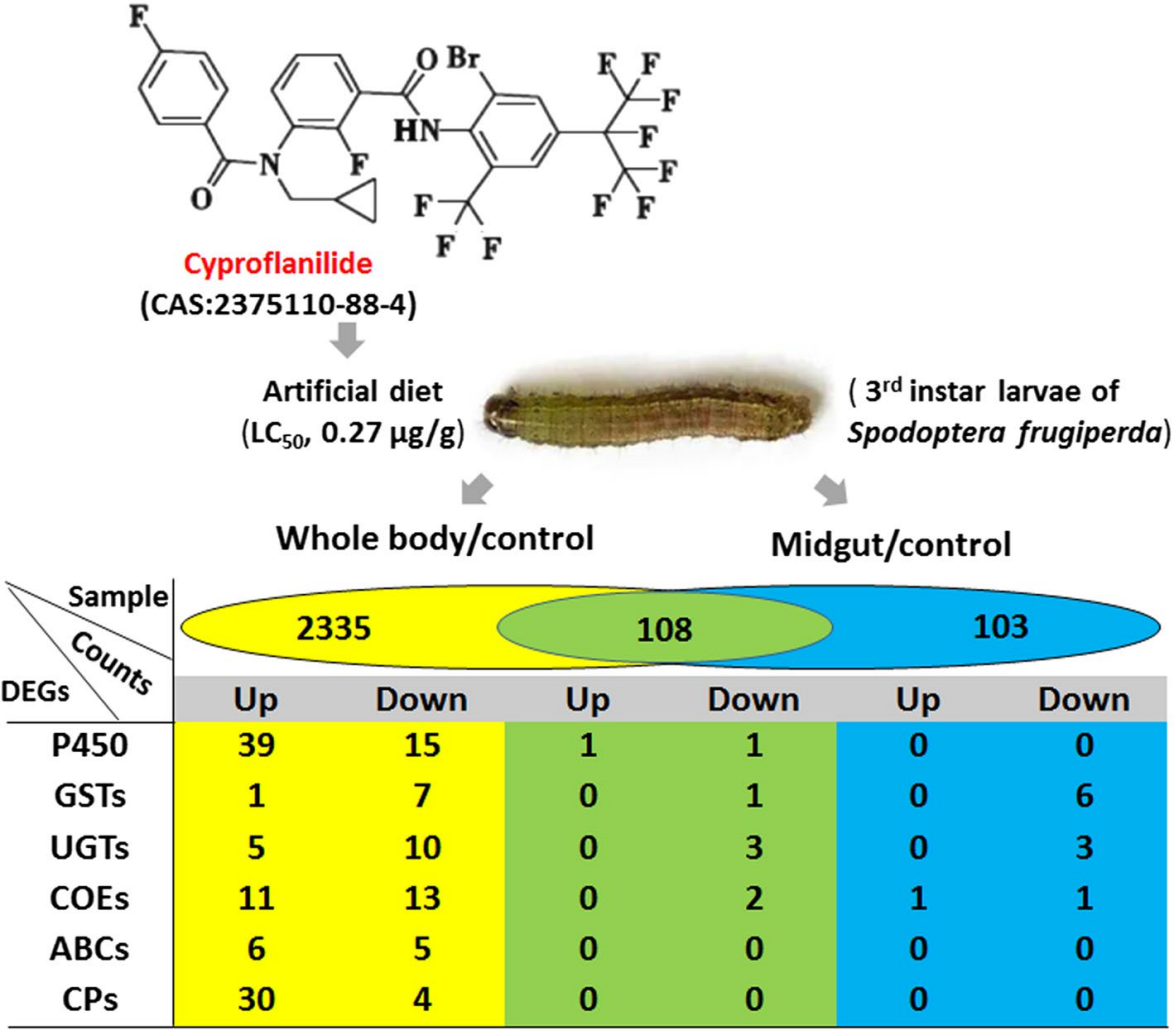
Partial DEGs of whole body and midgut of larvae under cyproflanilide stress

A total of 2443 (11.3%) differentially expressed genes (DEGs) were found between with the cyproflanilide treated larvae group and the control group, including 1326 up-regulated and 1117 down-regulated DEGs. Additionally, from the midgut samples, a total of 211 unigenes were differentially expressed between the cyproflanilide-treated and control groups, including 98 upregulated and 113 down-regulated DEGs. Among the above 2443 and 211 DEGs, our primary focus was on genes associated with detoxification metabolism (Fig. 2). The first major group of DEGs was comprised of genes involved in detoxification, which included genes encoding cytochrome P450 monooxygenases (P450s), glutathione S-transferases (GSTs), UDP glucosyltransferases (UGTs), carboxylesterases (COEs), and ATP-binding cassette transporters (ABCs). 131 detoxification genes were differentially expressed between the control and cyproflanilide-treated samples (Supplement table 5). The second major group of DEGs included 34 genes encoding cuticle proteins (CPs), which are critical structural components for insect tissues and influence the penetration efficiency of insecticides into the insect body (Supplement table 6). In larvae samples treated with cyproflanilide, 88.2% of CPs were up-regulated, whereas no CPs in the midgut were up- or down-regulated significantly.

**Fig. 3:**
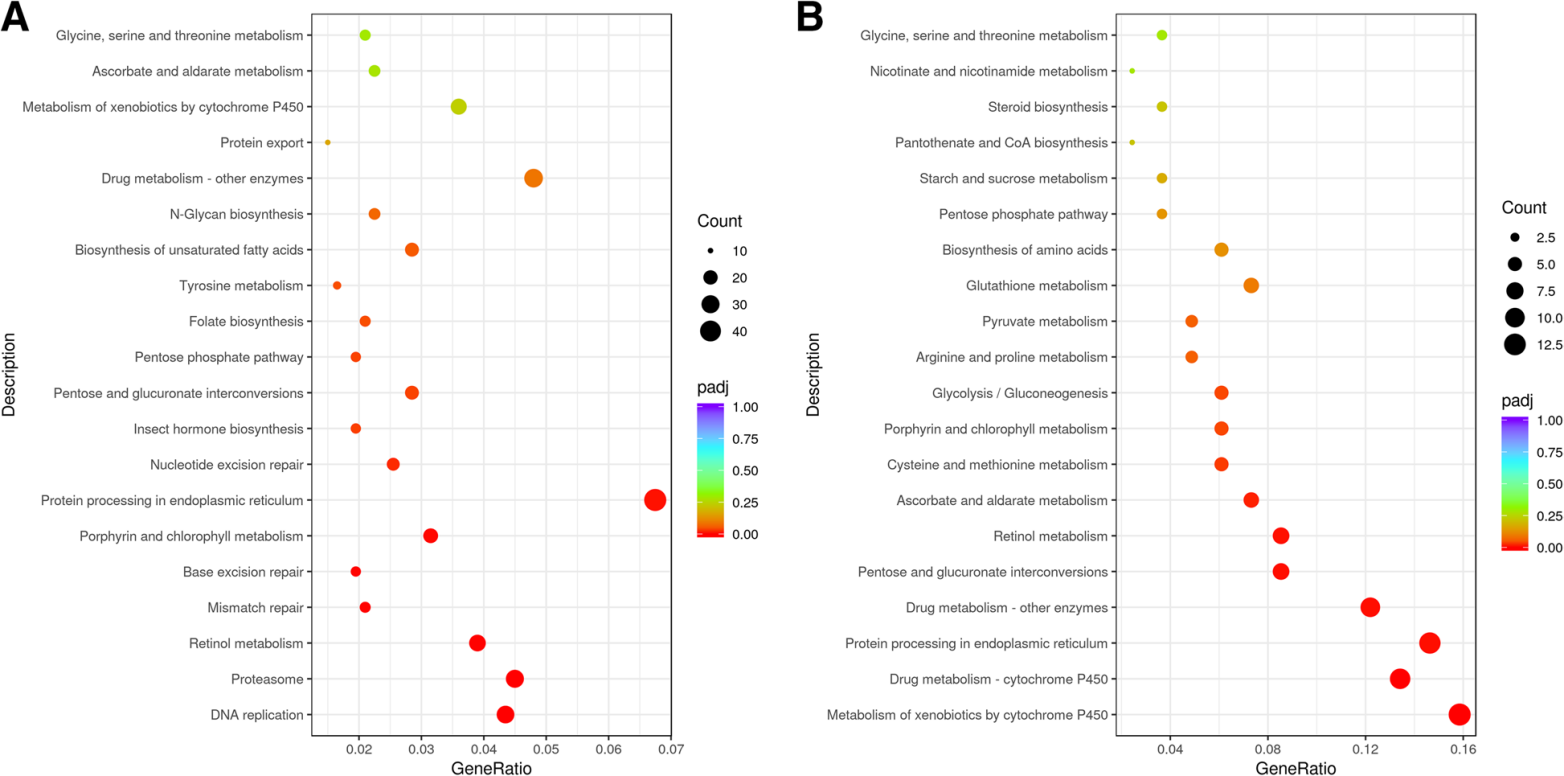
KEGG pathway analyses of identified DEGs from whole body and midgut. **A** The top 20 pathways enriched with DEGs obtained from whole body samples from larvae treated with cyproflanilide with FDR values. Among them, 13 pathways were significantly enriched with corrected *P*-values < 0.05. **B** 10 pathways significantly enriched with DEGs obtained from midgut samples (corrected *P*-values < 0.05). The x-axis represents rich factor. GeneRatio index means the ratio of the number of differential genes annotated to the KEGG pathway number to the total number of differential genes

KEGG (Kyoto Encyclopedia of Genes and Genomes) pathway enrichment analysis was assigned for DEGs under cyproflanilide stress. According to our findings, the most significantly enriched pathways in whole larvae body and midgut samples were ‘DNA replication’ (29 DEGs, 1.2%) and ‘Metabolism of xenobiotics by cytochrome P450’ (13 DEGs, 6.2%), respectively (Fig. 3). The number of DEGs in each category and the exact value of the P(adjust) are available in the additional files (Supplement table 7 and supplement table 8).

Current untested views predicated that cyproflanilide is a potent antagonist of the insect GABA receptor. In total, we obtained 12 unigenes which encode GABA receptors and one GABA receptor-associated protein. We found two metabotropic GABA(B) receptors and three ionotropic GABA(A) receptors were DEGs that were significantly up-regulated in whole body samples treated with cyproflanilide (Supplement table 9). Interestingly, both subunits of the metabotropic GABA(B) receptor belonged to DEGs. The human genome contains eight kinds of inotropic GABA(A) receptor subunits, including α, β, γ, δ, ε, π, θ, and ρ. Among the three candidate DEGs analyzed in this study (118,277,195, 118,263,634, and 118,273,029), all were annotated as probably subunit β-like. Additionally, none of the GABA receptors in midgut samples treated with cyproflanilide were DEGs.

### Comparative analysis of DEGs between the cyproflanilid, chlorantraniliprole, and avermectin treatment groups

**Fig. 4:**
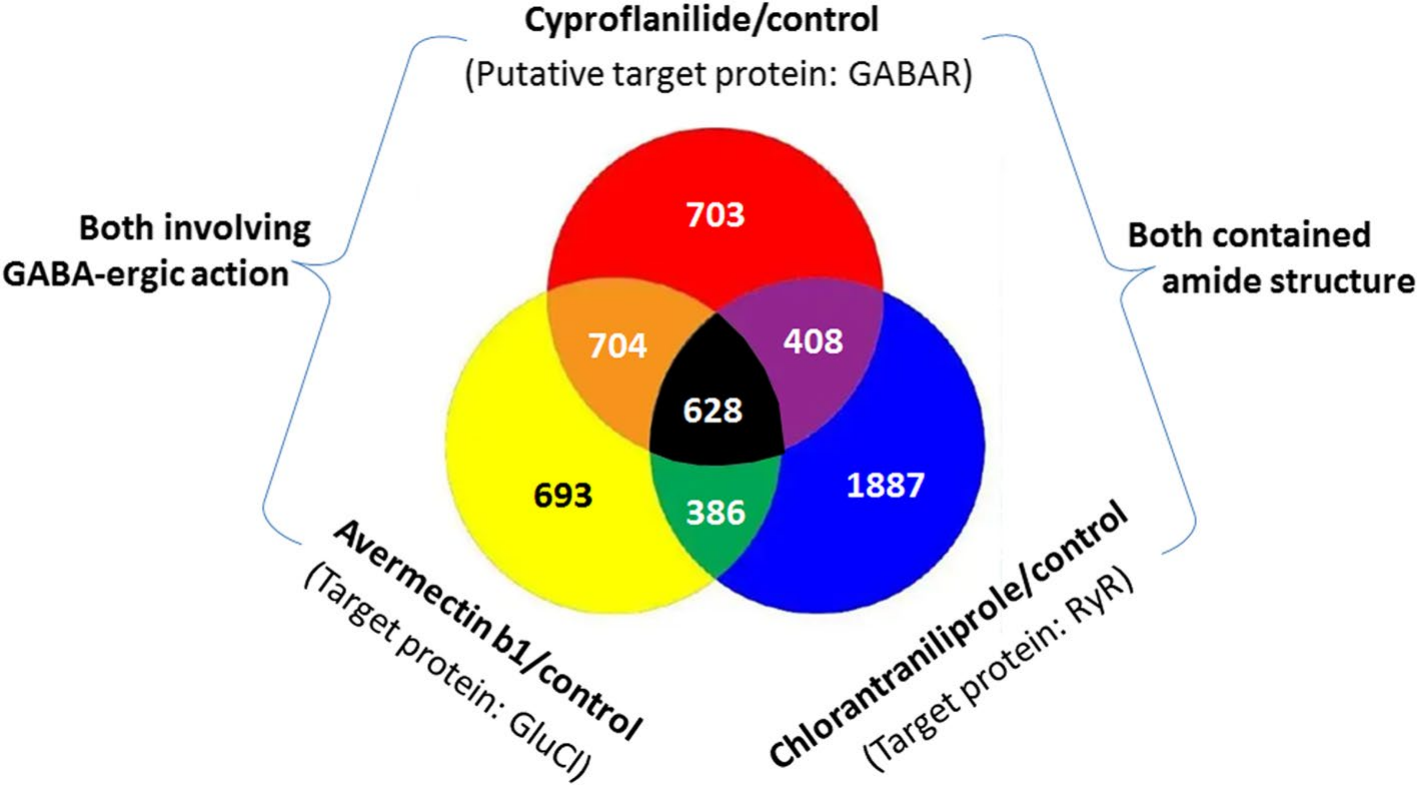
Venn diagram of DEGs obtained from the three comparative analyses

Using the same protocol as for cyproflanilide, whole body larval samples were treated with chlorantraniliprole and avermectin, respectively, for 24 h and then used for transcriptomic analyses. There were 3309 DEGs between the chlorantraniliprole-treated group and its control, and 2411 DEGs between the avermectin-treated group and its control. A venn diagram of DEGs was obtained from the comparative analyses of the three different treatment groups (cyproflanilide, chlorantraniliprole, and avermectin, in Fig. 4). We found 703 DEGs specific to cyproflanilide stress, accounting for approximately 28.8% of all DEGs in the cyproflanilide group. Additionally, 628 DEGs were shared by all three treatment groups, accounting for approximately 25.8% of all DEGs in the cyproflanilide group.

We initially focused on the P450 superfamily and CPs family, which are both associated with metabolic resistance to insecticides. There are 173 genes that encode P450 in the reference FAW genome used in this research. These genes were described in the annotation files of the FAW genome [18] (Supplement table 10), along with 9 gene loci yet to be confirmed that may be related to P450 members of another FAW genome [19] (Supplement table 11), and 252 genes that encode CPs (Supplement table 12). Of these, 93 P450s and 140 CPs belong to DEGs produced under the stress of these three insecticides (Table 1). The cause for the variation in P450 numbers between the three different insecticide treatments was not immediately obvious. In contrast, the deviation of CPs was significant, with only three (2.1%) CPs specific to cyproflanilide, and 69 (48.9%) CPs specific to chlorantraniliprole (Table 1). Interestingly, 40 (28.3%) CPs changed significantly under the stress of both cyproflanilide and avermectin, accounting for nearly one-third of the total CPs belonging to the DEGs identified under avermectin stress.

We also focused on DEGs associated with target proteins of these three insecticides. GABAR was presumed to be a likely target protein for the novel chemical cyproflanilide. We found that five GABARs belonged to DEGs under cyproflanilide-induced stress (Table 1, Supplement Table 13, Supplement Fig. 2). Furthermore, these five DEGs involved two metabotropic and three ionotropic GABARs. There were no GABAR that were specifically induced by cyproflanilide. Additionally, previous studies showed that the Glutamate-gated chloride channels (GluCl) [20] and ryanodine receptor (RyR) [21] were target proteins of avermectin and chlorantraniliprole, respectively. However, despite not being the target protein of cyproflanilide, one GluCl-like was found to be specifically induced by it (Table 1, Supplement Table 13).

### P450s and CPs distribution on chromosomes and analysis of transcriptional regulation under different insecticide-induced stresses

**Fig. 5:**
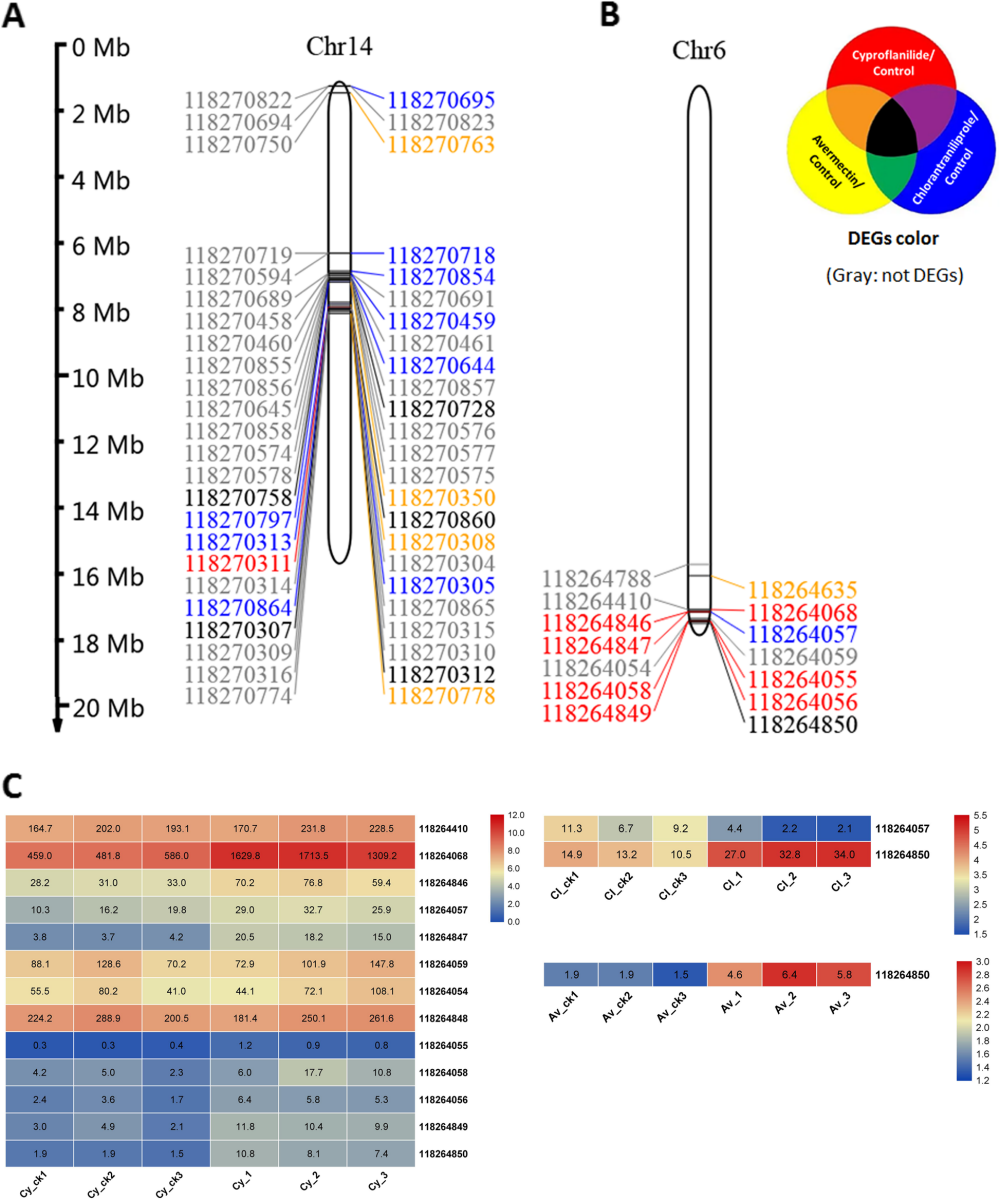
P450 distribution at the chromosomal level. **A**. chromosome 14 had the highest number of P450 genes; **B**. chromosome 6 displayed the highest level of P450 site compactness; **C**. heat map of DEGs on chromosomes 6 based on fragments per kilobase per million (FPKM). Cy, cyproflanilide; Av, avermectin; Cl, chlorantraniliprole; ck, control group

**Fig. 6:**
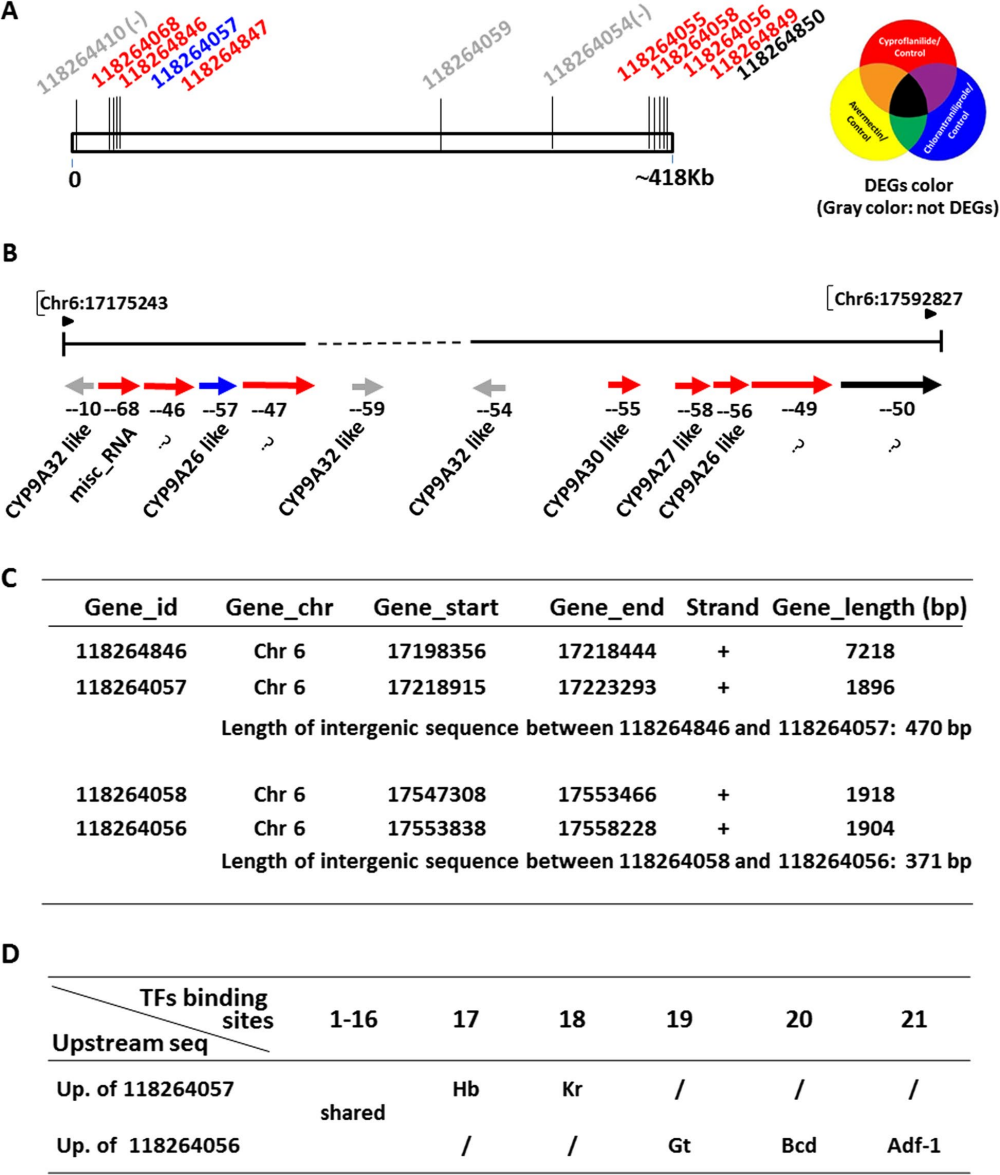
Expressional regulation analysis of P450s on the local region of the 6th chromosome. **A** Estimated position of the 12 P450s on chromosome 6; **B** Position and orientation of 12 P450s on chromosome 6; **C** Intergenic sequences between two representative groups of P450. **D** Transcription factor binding region of P450 promoter. Red color indicates DEGs that respond specifically to cyproflanilide; blue color indicates DEGs that respond specifically to chlorantraniliprole; black indicates DEGs responding to all three insecticides; gray indicates no response to any of the three insecticides. ‘– + number’ in Fig. 6B refers to the abbreviations of the accession number of P450 in Fig. 6A, with the last two digits retained for identification. ‘/–/’ refers to the intergenic sequences between two P450 genes. ‘?’ refers to gene loci that could contain multiple P450 genes (also were shown in Supplement table 11). The dashed ellipse indicates the differential binding site for transcription factors in the promoter region. Kr, Krueppel homolog 2 (CG9159 in *Drosophila melanogaster* genome); Hb, Hunchback (CG9786); Gt, Giant (CG7952), Bcd; Bicoid(CG1034); Adf-1, Adh distal factor-1(CG15845). 1–16 indicates 16 common transcription factors that were shared by the two above upstream sequences, including B factor, Bcd, Dfd, Dl, DSXF, DSXM, En, Eve, Ftz, Mad, Prd, SGF-3, Tll, Zen-1, Zeste [T00918],Zeste [T02100]

All 182 P450s mentioned above were distributed among the 32 chromosomes of FAW (Fig. 5, Supplement Fig. 3). Chromosome 14 has the largest number of P450, including 19 DEGs and 30 non DEGs (Fig. 5A). Chromosome 6 has a locus with a high P450 distribution density and contain nine DEGs within an approximately 418 Kb DNA regions (Fig. 5B, Fig. 6A). Figure 5C depicts the FPKM data of these DEGs on chromosome 6 for the three insecticides. Interestingly, one DEG (CYP9A26, No. 118264057) responded specifically to chlorantraniliprole (marked in blue color in Fig. 6B), while another DEG (No. 118264850) responded to all three insecticides (black color in Fig. 6B). The other seven P450s (No. 118264068, 118,264,846, 118,264,847, 118,264,055, 118,264,058, 118,264,056, 118,264,849) within the 418 Kb DNA regions responded specifically to cyproflanilide (red color in Fig. 6B). Many of these P450 genes are very close together, such as the 470 bp of the intergenic region between 118,264,846 and 118,264,057, and the 371 bp between 118,264,058 and 118,264,856 (Fig. 6C). We analyzed the regulatory pattern of the upstream sequence of P450 and found that both 118,264,057 and 118,264,856 were alignment to the same known CYP9A26 [19] (Fig. 6B). We then used an online tool (PROMO) [22] to determine the possible transcriptional factors binding to the promoter regions of these two P450 genes. We found binding sites for ‘Kr’ and ‘Hb’ [23, 24] in the upstream region of 118,264,057, and binding sites for ‘Gt’, ‘Bcd’ and ‘Adf-1’ [23, 24] in the upstream region of 118,264,856 (Fig. 6D, Supplement Fig. 4). These findings suggest differing responses of 118,264,856 and 118,264,057 to the two insecticide stresses, most likely due to different transcriptional factor binding or combinations. Further tests are needed to verify the true regulatory status of P450 genes and their responses to different insecticides.

**Fig. 7:**
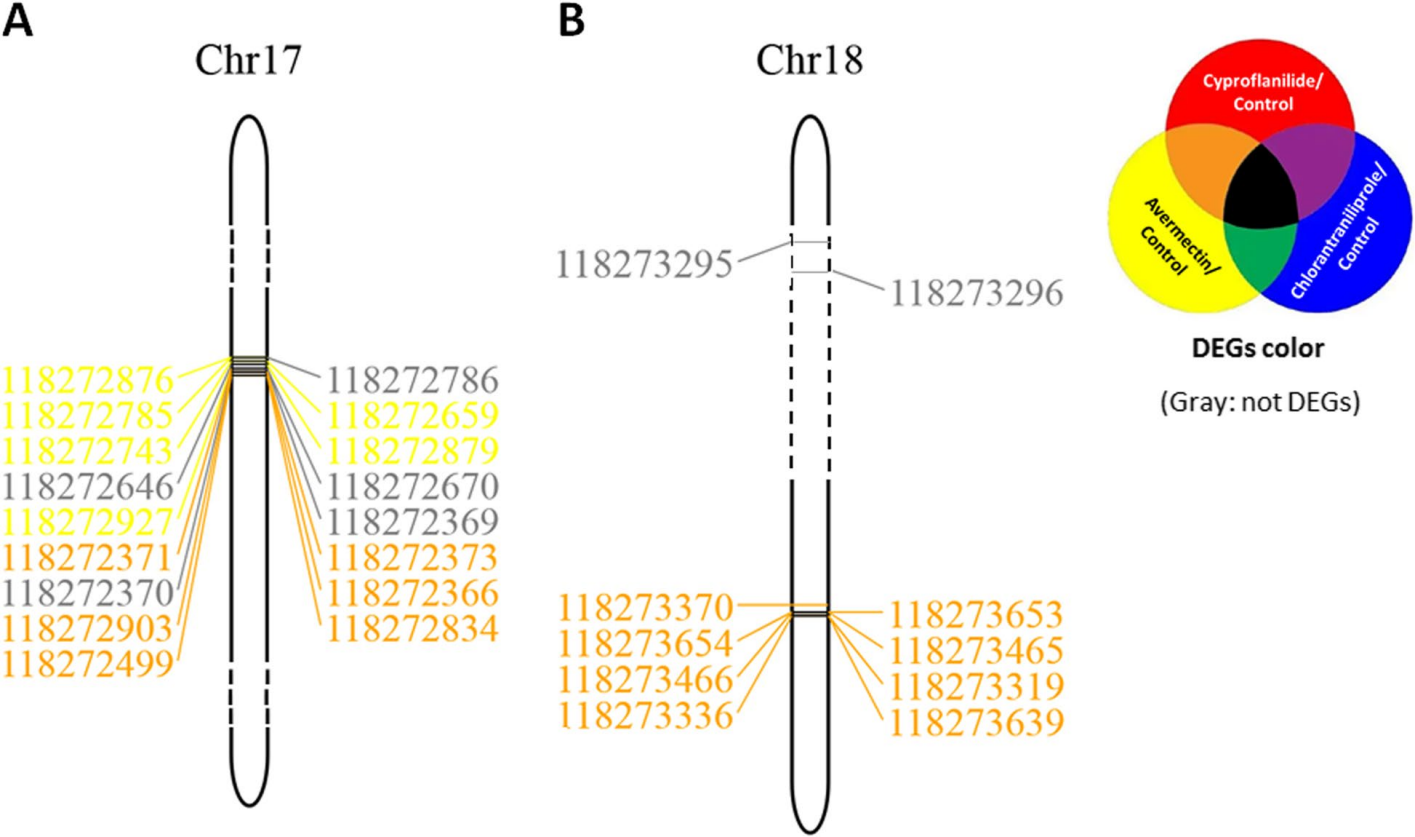
Cuticle protein (CP) distribution at the chromosomal level. **A** a representative group of genes appeared in response to avermectin or both cyproflanilide and avermectin within a compact region on chromosome; **B** a representative group of genes uniformly responding to cyproflanilide and avermectin within a compact region on chromosome. Yellow color of accession number indicates a P450 response specific to avermectin; brown color indicates a P450 response to both cyproflanilide and avermectin; gray color indicates no P450 response to any of the three insecticides

We also obtained 252 unigenes encoding CPs, 140 of which were DEGs that responded to one or all three insecticides. All the CPs were localized to 32 FAW chromosomes (Fig. 7, Supplement Fig. 5). In general, the response of DEGs to different insecticides would interleave on one chromosome (Fig. 7A). Interestingly, eight CPs located on the plus and minus strands of DNA within a relatively compact area of the 18^th^ chromosome showed a similar trend in response to cyproflanilide and avermectin stress (Fig. 7B). The intergenic region between every two CPs of the above eight CPs are over 3000 bp, hence we did not analyze the probable binding sites of the transcription factors as we did for P450. According to the description for these eight CPs (118,273,370, 118,273,653, 118,273,654, 118,273,465, 118,273,466, 118,273,319, 118,273,336, and 118,273,639), we found that seven (not including 118,273,653) of them encoded a larval/pupal rigid cuticle 66-like protein not found on any other chromosomes. Aside from a study that found larval/pupal rigid cuticle protein 66 is induced at low temperature [25], little is known about its function. Our future research will focus on whether these results indicate the possible development of cross-resistance in FAW to cyproflanilide and avermectin in FAW, and if the eight CPs, which showed similar response trends to both insecticides, could serve as potential cross-resistance DNA monitoring areas.

### Quantitative real-time PCR (qRT-PCR) validation

**Fig. 8:**
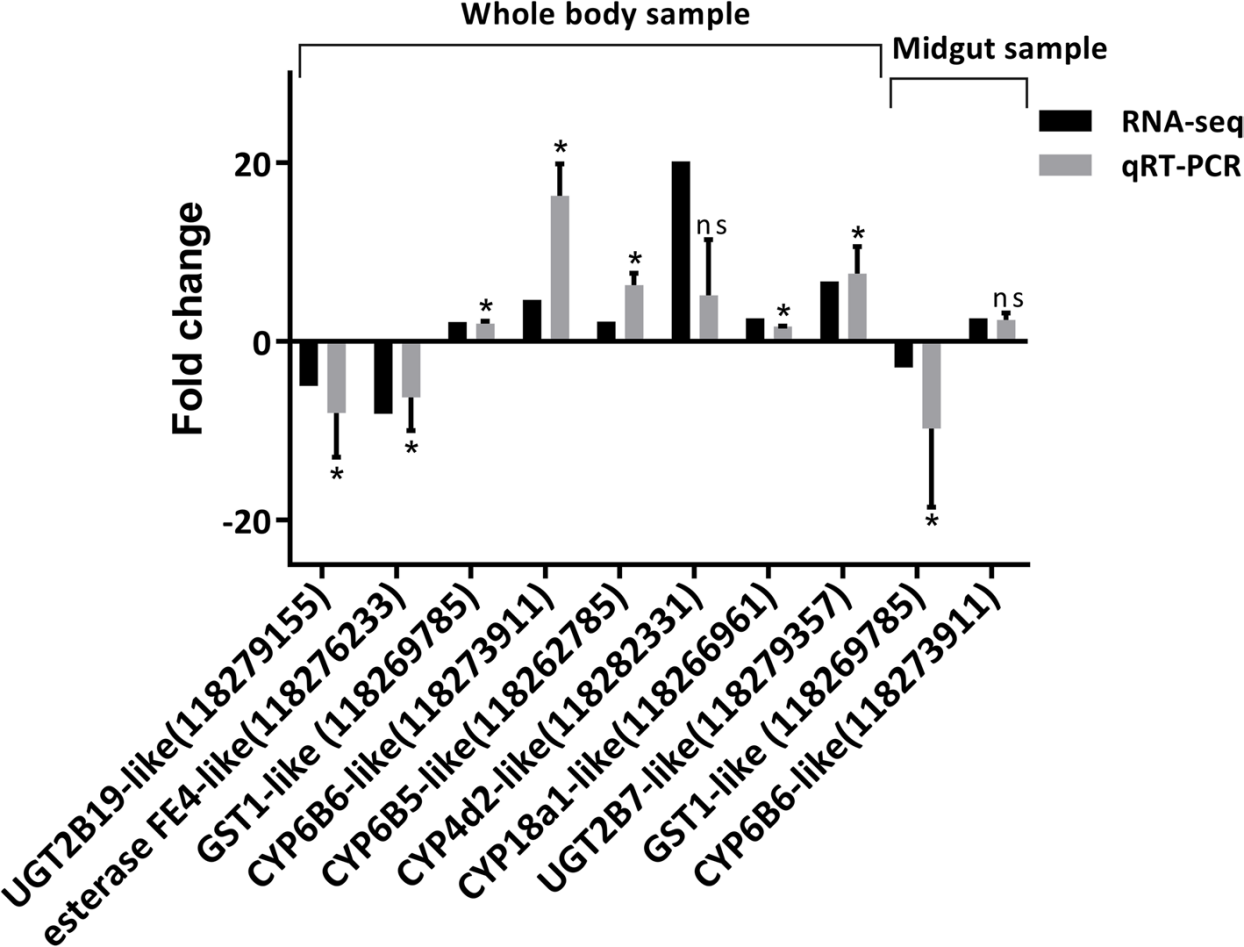
Quantitative real-time PCR (qRT-PCR) and RNA-Seq data of selected genes. Up-regulated or down-regulated DEGs were selected for PCR analysis and validation. Beta-actin was used as the reference gene for qRT-PCR normalization. mRNA expression levels for the selected genes were calculated using the 2^−△△CT^ method. * indicates significant difference (*p* < 0.05) between treatment and control based on student’s *t* tests

To validate the transcriptomic analysis results, eight DEGs from whole larvae body samples and two DEGs from midgut samples involved in detoxification were selected for qRT-PCR validation (Fig. 8). All DEGs, except for two candidates (118,282,331, 118,273,911), differed significantly between the cyproflanilide and control treatment groups. However, the upward and downward trends in gene expression levels were the same for both groups. Interestingly, GST1-like (118,269,785) was upregulated in the midgut but downregulated in whole body samples under cyproflanilide stress. The changes in gene expression levels of above candidates based on qRT-PCR were basically consistent with the transcriptomic data.

## Discussion

Since it was first detected in 2019, FAW has become a serious insect pest in China 2019 [5]. During the past three years, multi-site trials have examined the effects of a novel chemical, cyproflanilide, on FAW and the results confirm that cyproflanilide has the potential to be an effective insecticide against FAW. Several field studies have evaluated the efficacy of various insecticides in the control of this pest, including chlorantraniliprole, avermectin, spinetoram, and emamectin benzoate [26, 27]. Although FAW has not yet developed obvious resistance to cyproflanilide, chlorantraniliprole, or avermectin in China, resistance of FAW will undoubtedly evolve and occur when insecticides are used incorrectly. Previous studies analyzing genes related to pesticide- and Bt-resistance in FAW found that the risk of FAW developing resistance to conventional insecticides, such as organophosphates and pyrethroids, is very high [28]. Cyproflanilide will almost certainly be promoted for use in the field in China, if not globally, in the coming years. Thus, it is important to clarify in advance the similarities, differences, and potential cross resistance between cyproflanilide and other commonly used insecticides.

Broflanilide, which is chemically similar to cyproflanilide, has been reported to cause symptoms of toxicity in zebrafish such as reduced heart rate and shortened larval body length [29]. In FAW, sublethal doses of broflanilide can cause failure of ecdysis, reduced body length of larvae, malformation of pupae, and vestigial wing formation in adults [30]. In this study, a sublethal dose (LC_10_ and LC_30_) of cyproflanilide did not significantly affect FAW pupae weight or the reproductive capacity of adults (Fig. 1; Supplement Table 1, 2). However, the median lethal dose of cyproflanilide has a clear toxic effect on larvae. The insect midgut is an important organ responsible for food digestion and nutrient absorption. Whether the primary site of action for cyproflanilide against FAW is in the midgut remains unknown. In this study, we did not observe any obvious intestinal wall degradation in the midgut of FAW after feeding on an artificial diet mixed with a lethal dose of cyproflanilide.

Transcriptomic analysis is a common method for identifying differentially expressed genes in insects in response to toxic compounds, particularly for novel chemicals where the mechanisms of action in insects is unclear [9, 31]. Since 2019, transcriptomic analyses have been conducted to identify DEGs in Chinese populations of FAW in response to 23 pesticides, including 4 biological, 10 single, and nine mixed chemical pesticides [32], among which we identified chlorantraniliprole. However, the study used a 35% chlorantraniliprole water dispersible granule, whereas we used 98% raw chlorantraniliprole dissolved in DMSO and tween 80. The novel chemical cyproflanilide was obviously not among the above 23 pesticides. In general, insect responses to different insecticides would differ in terms of transcriptional expressions. By merging DEGs following 23 different pesticide treatments, Gui et al. (2022) [32] obtained a union set with 7,991 DEGs, including 107 differentially expressed P450 genes. In our study, we obtained 14 differentially expressed P450 genes that responded specifically to cyproflanilide, and 35 differentially expressed P450 genes that were co-induced by chlorantraniliprole and avermectin (Table 1). When we compared these two sets of P450 we found four P450 genes that were significantly up-regulated and one P450 genes that was significantly down-regulated under cyproflanilide stress but were not DEGs when under stress from 23 pesticides [32] (Supplement Table 14). Based on the analysis of the above 26 pesticides, it is very likely that these four P450 genes were specifically up-regulated by cyproflanilide. The expression profiles of these four P450 genes, their modes of action, and whether they mediate the metabolism of cyproflanilide requires further research.

GABAR-targeted insecticides such as broflanilide, fluralaner, and fipronil exhibit high toxicity against lepidopteran pests [33]. Studies suggest that the target site of meta-diamides such as broflanilide differs from that of conventional noncompetitive inhibitors such as fipronil. Furthermore, meta-diamides are effective against fipronil-resistant pests that carry target-site mutations [34]. Cyproflanilide is a potent insect GABA receptor antagonist classified as a member of group 30 by the IRAC. However, in the absence of resistant strains and electrophysiological studies of FAW, we cannot currently verify the GABA receptor target. Broflanilide has been shown to influence the transcripts of genes associated with dopamine and GABA expression [35]. In this study, the transcripts of genes associated with GluCl and GABAR were also affected under cyproflanilide stress, even though GluCl is not the target protein of cyproflanilide (Table 1). Additionally, cyproflanilide-induced stress significantly upregulated the expression of three ionotropic GABA (A) receptors, the likely target of cyproflanilide, as well as the expression of two metabotropic GABA (B) receptors. Whether the upregulation of the target protein expression was related to the interaction between the insecticide and its target protein remains unknown. GABA (B) belongs to class C of the G protein-coupled receptors. Unfortunately, we were not able to obtain information regarding how GABA (B) interacted with other candidates within the predicted signal pathways of the G protein. Hence, based on the known interactions of the reference species, the GABA (B) network of FAW under cyproflanilide stress could not be established.

Cuticle proteins (CPs) play a crucial role in various physiological processes including cuticle integration, body shape, activity, resistance, and innate immunity. Furthermore, they are an essential structural component of the insect midgut peritrophic membrane. Surprisingly, there was no difference in the number of differentially expressed CPs genes in the midgut between larvae treated with cyproflanilide and the control larvae (Fig. 2) which supports our assumption that the site of action of cyproflanilide is unlikely the midgut. In contrast, we found that 92.6% of CPs were upregulated in whole body samples of larvae treated with cyproflanilide. While insect target sites and metabolic resistance have been studied extensively, other resistance mechanisms exist outside of these paradigms. One such process involves reducing the penetration of insecticides into the insect body by increasing the thickness or modifying the composition of the cuticle, primarily through enhanced deposition of structural components, such as epicuticular lipids and/or structural cuticular proteins [36–38]. Interestingly, *Anopheles gambiae* was found to have remarkable tolerance to multiple insecticide classes and had thicker leg cuticles as a result of enriched epicuticle hydrocarbon deposition. Underpinning this phenotype was the over-expression of two cytochrome P450s (CYP4G16 and CYP4G17) in the abdominal oenocytes, which synthesized the extra hydrocarbons [39]. Thus, there could be important unknown networks of relationships between the CPs and P450, the two major gene families focused on in this study. Additionally, we also found some interesting CP data including 40 (28.3%) CP genes that changed significantly under both cyproflanilide and avermectin stress (Table 1). Given that the novel chemical cyproflanilide will be commercially available in approximately two years, we hope our study provides valuable information for improving the implementation of proper application methods in the field and managing insect resistance.

## Conclusions

In conclusion, growth inhibition and lethal effects were observed in FAW larvae treated with the novel chemical, cyproflanilide. Comparative transcriptomic analyses identified numerous DEGs triggered by cyproflanilide, chlorantraniliprole and avermectin, either together or individually. Our research analyzed the similarities, differences, and correlations of multiple DEG genes, focusing on the P450 superfamily, CP genes, and neurotransmitter receptor genes, under the stress of three insecticides. Our results provide the foundation for future research on the basic mechanism of cyproflanilide and its potential practical application in FAW control.

## Methods

### Insect rearing

A laboratory colony of FAW was provided by the South China Agricultural University in 2020 and maintained for more than 30 generations in our laboratory under a 16:8 h (L:D) photoperiod at 26 ± 1℃ and 80% relative humidity. Third-instar larvae were feed 1.5 kg of an artificial diet made up of 240 g vitakraft, 225 g wheat germ slice, 15 g yeast, 45 g agar, 12 g vitamin C, 5.7 g methyl 4-hydroxybenzoate, and 1.5 g sorbic acid. Adults were fed 10% honey water.

### Bioassays of cyproflanilide on *S. frugiperda*

Cyproflanilide (98.91%) was provided from CAC Nantong Taihe Chemical CO. Ltd. (China). The Bioassays on third-instar *S. frugiperda* larvae were performed using the diet incorporation method. Cyproflanilide was dissolved in 10 mL acetone to create a primary solution of 1000 mg/L. The concentrations were selected based on the results of a pre-bioassay. Seven concentrations (2.90, 3.48, 4.18, 5.02, 6.04, 7.24, 8.70 mg/L) of working-solution were serially diluted from the primary solution using acetone. The control group was treated with acetone only. For the diet incorporation method, 1 mL of working-solution was mixed into 20 g of artificial diet to produce seven gradient concentrations (0.145, 0.174, 0.209, 0.251, 0.302, 0.362, 0.435 μg/g). Thirty third-instar larvae were selected for each cyproflanilide treatment gradient and left for 24 h. Three biological replicates were carried out per treatment.

Here is the treatment evaluation and statistical analysis. The third-instar larvae were considered dead there was no physical reaction when touched with a fine soft-pointed brush. If the mortality rates in the control groups ranged from 5 to 20%, the mortality of larvae was calculated using the adjusted mortality method according to Abbott's formula: adjusted mortality (%) = 100 × (mortality in the pesticide-treated group—mortality in the control group)/(100 − mortality in the control group). If the mortality rates in the control group were above 20%, the experiment was repeated. The results, including LC with corresponding 95% confidence limits (CL) and chi- square (χ^2^), and *P* values, were calculated using probit regression analysis in SPSS Version 22. Data from the sublethal effect experiments were considered as significantly different if *P* < 0.05.

### Exposure test of cyproflanilide, avermectin and chlorantraniliprole for transcriptomic analysis

Avermectin b1 (91.5%) and chlorantraniliprole (98%) were purchased from Hunan Nongjie Biotechnology Co.,Ltd (China), and were dissolved in acetone and DMSO (mixed with 1% tween 80), respectively. Healthy third-instar FAW larvae of the same size were selected for the experiment and fed on an artificial diet containing cyproflanilide, avermectin b1, or chlorantraniliprole for 48 h, according to the LC_50_ of the FAW biological assay test. Larvae in the control group were fed the artificial diet with acetone or DMSO. Because both the cyproflanilide and avermectin treatments were dissolved in acetone they shared the same control group. Each treatment or control group had three replicates with thirty larvae in each..

### Samples collection and Illumina sequencing

Three groups of thirty larvae were exposed one of three insecticides: cyproflanilide, avermectin b1, or chlorantraniliprole. Additionally, 150 larval midgut samples were dissected from other larvae which were also exposed to cyproflanilide. All samples were stored at -80℃ and sent on dry ice to Novogene Co., Ltd. in Tianjing (China) for transcriptome sequencing within three days of arrival. The clustering of the index-coded samples was sequenced on an Illumina Novaseq 6000 platform and 150 bp paired-end reads were generated. Reference genome and gene model annotation files [18] were downloaded directly from the relevant genome website (https://ftp.ncbi.nlm.nih.gov/genomes/all/GCF/011/064/685/GCF_011064685.1_ZJU_Sfru_1.0/GCF_011064685.1_ZJU_Sfru_1.0_genomic.gff.gz). Quantification of gene expression levels was based on fragments per kilobase per million (FPKM). Differential expression analysis between different treatments was performed using the DESeq2 R package (1.20.0). Genes with an adjusted *P*-value < 0.05 found by DESeq2 were considered to be differentially expressed. A HeatMap of DEGs based on FPKM data was drawn using the software TBtools [40].

### Chromosomes and transcriptional regulatory analysis

FAW chromosome gene mapping was carried out using online software (http://mg2c.iask.in/mg2c_v2.1/) with default parameters, according to the software parameters setting guide [41]. The location information for each gene was obtained from the annotation files of the reference genome. Transcriptional factor binding site prediction was carried out using the online software PROMO [22] (http://alggen.lsi.upc.es/cgi-bin/promo_v3/promo/promoinit.cgi?dirDB=TF_8.3). The upstream sequence of each transcript or DEG was obteined from the genome sequence file, based on the location site of each transcript in the genome. If the size of the intergenic region exceeded 3000 bp, only the 3000 bp nucleotides closest to the target transcript were taken. The parameters of SelectSpecies and SelctFactor were both selected as ‘insecta’. The parameters of maximum matrix dissimilarity rate were set at 15%.

### RNA isolation and qRT-PCR for revalidation of the expression levels of candidate genes

Sample preparation for qRT-PCR was the same as that described above for transcriptome analysis. Ground FAW samples were placed in RNase-free tubes and 1 ml of Trizol (TaKaRa, Dalian, China) lysed tissue was added. Trichloromethane was added to extract the RNA layer (supernatant) followed by isopropanol to obtein the RNA precipitation. The RNA precipitation was then washed with 75% ethanol and the final precipitation was dissolved in water-DEPC. 1 µg of total RNA was used to synthesize the first-strand cDNA using a PrimeScript™ RT reagent kit with gDNA eraser (TaKaRa, Dalian, China) following the manufacturer’s instructions. qRT-PCR primers of candidate genes (Supplement Table 15) were designed using information from the National Center for Biotechnology Information profile server (http://www.ncbi.nlm.nih.gov/tools/primer-blast). We first assessed the efficiency of the qRT-PCR primers using a fivefold dilution series of cDNA corresponding to 1 µg total RNA. This was used to produce a standard curve (cDNA concentration vs. Ct) [42]. qRT-PCR was carried out in quintuplicate using TB Green Premix Ex TaqTM II (TaKaRa) on a CFX96 real-time PCR system (BIO-RAD). The qRT-PCR protocol was as follows: one cycle of denaturation at 94 °C for 30 s, followed by 40 cycles of denaturation at 95 °C for 5 s, annealing at 60 °C for 30 s, followed by a melting curve analysis. The endogenous normalization controls were beta-actin [43]. To calculate the relative expression levels of each candidate gene, the arithmetic mean values of beta-actin were used as the internal references. Gene expression data was analyzed using the 2^−ΔΔCt^ method.

### Data analysis

The statistical significance of differences between treatment and control means were assessed using Student’s *t*-test. All analyses and figures were prepared using GraphPad Prims 7 software and Adobe PhotoShop.

## Supplementary Information

**Additional file 1:** **Supplement Table 1.** Sub-lethal effects of cyproflanilide against FAW on developmental periods. **Supplement Table 2.** Sub-lethal effects of cyproflanilide against FAW on several developmental indexes. **Supplement Table 3.** Sub-lethal effects of cyproflanilide against FAW on body length. **Supplement Table 4.** Information of high quality reads from different sample groups. **Supplement Table 5.** Information of 131 DEGs involved in detoxification (corresponding to data in figure 2). **Supplement Table 6.** Information of 34 cuticle proteins (CPs) (corresponding to data in figure 2). **Supplement Table 7.** KEGG pathway enrichment for DEGs of larvae (whole body) under cyproflanilide stress. **Supplement Table 8.** KEGG pathway enrichment for DEGs of larval midgut samples under cyproflanilide stress. **Supplement Table 9.** Expression profile of GABA receptor candidates of larvae treated with cyproflanilide. **Supplement Table 10.** P450 genes which were descripted in the annotation files of FAW genome (Xiao et al. 2020). **Supplement table 11.** Nine gene loci in the FAW genome which possible relates to P450 family. **Supplement Table 12.** CPs genes which were descripted in the annotation files of FAW genome (Xiao et al. 2020). **Supplement Table 13.** P value associated to fold change of seven target proteins. **Supplement Table 14.** Comparison and analysis of two sets of differentially expressed P450 genes. **Supplement Table 15.** Real-time PCR primer. **Supplement Figure 1.** Liner regression of morality (probit unit) of *Spodoptera frugiperda* and cyproflanilide concentration (Log transformed). **Supplement Figure 2.** Heat map of DEGs on different target proteins based on fragments per kilobase per million (FPKM). **Supplement Figure 3.** P450s scattered among different chromosomes of *Spodoptera frugiperda*. **Supplement Figure 4.** TF binding site prediction in the upstream of DGEs. **Supplement Figure 5.** CPs scattered among different chromosomes of *Spodoptera frugiperda*.

## Data Availability

The datasets generated and analyzed during the current study are available in the NCBI SRA database, with the accession number from SRA:SRS15030500 to SRA:SRS15030520 (BioProject: PRJNA877689) (https://www.ncbi.nlm.nih.gov/bioproject/?term=PRJNA877689).

## Abbreviations

ABCs: ATP-binding cassette transporters
Bt: Bacillus thuringiensis
COEs: Carboxylesterases
CPs: Cuticle proteins
P450s: Cytochrome P450 monooxygenases
DEGs: Differentially expressed genes
FAW: Fall armyworm
FPKM: Fragments per kilobase per million
GSTs: Glutathione S-transferases
GluCl: Glutamate-gated chloride channels
HE: Hematoxylin–eosin
IRAC: Insecticide Resistance Action Committee
ISO: International Organization for Standardization
KEGG: Kyoto Encyclopedia of Genes and Genomes
GABAR: γ-Aminobutyric acid receptor
RyR: Ryanodine receptor
TF: Transcription factor
UGTs: UDP glucosyltransferases
qRT-PCR: Quantitative real-time PCR
