# Supplementary material for: The transcriptomic profile of Spodoptera frugiperda differs in response to a novel insecticide, cyproflanilide, compared to chlorantraniliprole and avermectin
Source: BMC Genomics. 2023 Jan 3;24:3. doi: 10.1186/s12864-022-09095-2 (PMC9811769; doi:10.1186/s12864-022-09095-2)
Supplement: Supplementary file 1 — Additional file 1: Supplement Table 1. Sub-lethal effects of cyproflanilide against FAW on developmental periods. Supplement Table 2. Sub-lethal effects of cyproflanilide against FAW on several developmental indexes. Supplement Table 3. Sub-lethal effects of cyproflanilide against FAW on body length. Supplement Table 4. Information of high quality reads from different sample groups. Supplement Table 5. Information of 131 DEGs involved in detoxification (corresponding to data in figure 2). Supplement Table 6. Information of 34 cuticle proteins (CPs) (corresponding to data in figure 2). Supplement Table 7. KEGG pathway enrichment for DEGs of larvae (whole body) under cyproflanilide stress. Supplement Table 8. KEGG pathway enrichment for DEGs of larval midgut samples under cyproflanilide stress. Supplement Table 9. Expression profile of GABA receptor candidates of larvae treated with cyproflanilide. Supplement Table 10. P450 genes which were descripted in the annotation files of FAW genome (Xiao et al. 2020). Supplement table 11. Nine gene loci in the FAW genome which possible relates to P450 family. Supplement Table 12. CPs genes which were descripted in the annotation files of FAW genome (Xiao et al. 2020). Supplement Table 13. P value associated to fold change of seven target proteins. Supplement Table 14. Comparison and analysis of two sets of differentially expressed P450 genes. Supplement Table 15. Real-time PCR primer. Supplement Figure 1. Liner regression of morality (probit unit) of Spodoptera frugiperda and cyproflanilide concentration (Log transformed). Supplement Figure 2. Heat map of DEGs on different target proteins based on fragments per kilobase per million (FPKM). Supplement Figure 3. P450s scattered among different chromosomes of Spodoptera frugiperda. Supplement Figure 4. TF binding site prediction in the upstream of DGEs. Supplement Figure 5. CPs scattered among different chromosomes of Spodoptera frugiperda. [file 12864_2022_9095_MOESM1_ESM.docx]

**Supplement files for**

**The transcriptomic profile of *Spodoptera frugiperda* differs in response to a novel insecticide, Cyproflanilide, compared to chlorantraniliprole and avermectin**

**Haijuan Shu^1^, Yufeng Lin^2^, Zhengbing Zhang^2^, Lin Qiu^1^, Wenbing Ding^1,3^, Qiao Gao^1^, Jin Xue^1^, Youzhi Li^1,3^, Hualiang He^1,^***

^1^Hunan Provincial Key Laboratory for Biology and Control of Plant Diseases and Insect Pests, College of Plant Protection, Hunan Agricultural University, Changsha, 410128, China.

^2^Agriculture and Rural Department of Hunan Province, Plant Protection and Inspection Station, Changsha, 410005, China.

^3^National Research Center of Engineering & Technology for Utilization of Botanical Functional Ingredients, Hunan Agricultural University, Changsha, 410128, China.

**Supplement Table 1 Sub-lethal effects of cyproflanilide against FAW on developmental periods**

| **Treatments** | **3L(d)** | **4L(d)** | **5 L(d)** | **6 L(d)** | **Pupal stage (d)** | **Adult (d)** |
| --- | --- | --- | --- | --- | --- | --- |
| **Control** | 1.40±0.06c | 1.99±0.06b | 2.51±0.07c | 3.29±0.07a | 8.78±0.10a | 9.12±0.33a |
| **LC_10_** | 1.89±0.12b | 2.79±0.12a | 3.09±0.09b | 3.52±0.10a | 8.91±1.41a | 8.65±0.30a |
| **LC_30_** | 2.6±0.92a | 2.80±0.20a | 3.60±0.51a | 3.40±0.51a | 9.00±0.63a | 8.20±0.58a |

Control, indicates larvae were fed the artificial diet without insecticide, but with acetone. Data were mean ± SE of three replication. Means within a column followed by different letters are significantly different by Duncan’s new multiple range test (*P*<0.05).

**Supplement Table 2 Sub-lethal effects of cyproflanilide against FAW on several developmental indexes**

| **Treatments** | **Counts** | **Pupation (%)** | **Pupal weight(mg)** | **Emergence (%)** | **Eggs per female** |
| --- | --- | --- | --- | --- | --- |
| **Control** | 90 | 98.89±1.11a | 198.73±2.06a | 95.56±1.11a | 953.13±96.26a |
| **LC_10_** | 90 | 90.00±3.85a | 197.99±2.29a | 84.45±2.22b | 782.53±100.21a |
| **LC_30_** | 90 | 80.00±3.85b | 186.67±11.84a | 72.22±4.45c | 791.13±101.49a |

Control, indicates larvae were fed the artificial diet without insecticide, but with acetone. Data were mean ± SE of three replication. Means within a column followed by different letters are significantly different by Duncan’s new multiple range test (*P*<0.05).

**Supplement Table 3 Sub-lethal effects of cyproflanilide against FAW on body length**

| **Cyproflanilide treatment** | **Larval body length (mm)** | |
| --- | --- | --- |
|  | **24 h** | **48 h** |
| **Control** | 15.39±0.35a | 18.41±0.55a |
| **LC_50_** | 13.06±0.21b^＆^ | 11.65±0.29b^＆^ |
| **LC_50_** | 7.74±0.25c^#^ | 8.20±0.22c^#^ |

Control, indicates larvae were fed the artificial diet without insecticide, but with acetone. Data were mean ± SE of three replication. Means within a column followed by different letters are significantly different by Duncan’s new multiple range test (*P*<0.05). ＆, body length of alive larvae; #, body length of dead larvae.

**Supplement Table 4 Information of high quality reads from different sample groups**

| Sample | Library | Raw_reads | Clean_reads | Clean_bases | Error_rate | Q20 | Q30 | GC_pct |
| --- | --- | --- | --- | --- | --- | --- | --- | --- |
| CyM_1 | FRAS210164705-1r | 46762258 | 44578656 | 6.69G | 0.03 | 97.39 | 93.16 | 46.7 |
| CyM_2 | FRAS210164706-1r | 44755902 | 42655826 | 6.4G | 0.03 | 97.72 | 93.83 | 45.71 |
| CyM_3 | FRAS210164707-1r | 42661388 | 39249884 | 5.89G | 0.02 | 98.47 | 95.43 | 46.68 |
| CyM_Ck_1 | FRAS210164702-1r | 46043524 | 43701250 | 6.56G | 0.03 | 97.42 | 93.2 | 46.29 |
| CyM_Ck_2 | FRAS210164703-1b | 53396952 | 52454226 | 7.87G | 0.03 | 97.87 | 93.74 | 43.5 |
| CyM_Ck_3 | FRAS210164704-1r | 43563382 | 40920530 | 6.14G | 0.03 | 97.43 | 93.27 | 46.73 |
| Ck1_1 (for Cy and Av) | FRAS210164687-1r | 44324094 | 41878826 | 6.28G | 0.03 | 97.74 | 93.9 | 47.11 |
| Ck1_2 (for Cy and Av) | FRAS210164688-1r | 43563738 | 41150982 | 6.17G | 0.03 | 97.39 | 93.17 | 46.83 |
| Ck1_3 (for Cy and Av) | FRAS210164689-1r | 44484756 | 42506436 | 6.38G | 0.03 | 97.6 | 93.64 | 47.06 |
| Cy_1 | FRAS210164690-1r | 42288626 | 40407840 | 6.06G | 0.03 | 97.52 | 93.44 | 46.54 |
| Cy_2 | FRAS210164691-1r | 42830984 | 40463928 | 6.07G | 0.03 | 97.6 | 93.65 | 47.69 |
| Cy_3 | FRAS210164692-1r | 51724046 | 49448386 | 7.42G | 0.03 | 97.58 | 93.57 | 47.75 |
| Av_1 | FRAS210164693-1r | 46816412 | 44937920 | 6.74G | 0.03 | 97.44 | 93.29 | 47.85 |
| Av_2 | FRAS210164694-1r | 49915606 | 47974886 | 7.2G | 0.03 | 97.92 | 94.22 | 48.16 |
| Av_3 | FRAS210164695-1r | 43580844 | 41532960 | 6.23G | 0.03 | 97.54 | 93.48 | 48.21 |
| Cl_1 | FRAS210164699-1r | 44204004 | 41956792 | 6.29G | 0.03 | 97.78 | 93.96 | 47.81 |
| Cl_2 | FRAS210164700-1r | 43128156 | 40261882 | 6.04G | 0.03 | 97.4 | 93.28 | 49.07 |
| Cl_3 | FRAS210164701-1r | 45990208 | 42940158 | 6.44G | 0.03 | 97.64 | 93.71 | 48.76 |
| Ck2_1 (for Cl) | FRAS210164696-1r | 41898580 | 39964420 | 5.99G | 0.03 | 97.9 | 94.23 | 49.43 |
| Ck2_2 (for Cl) | FRAS210164697-1r | 41854284 | 40361196 | 6.05G | 0.03 | 97.9 | 94.2 | 48.87 |
| Ck2_3 (for Cl) | FRAS210164698-1r | 40221888 | 38054356 | 5.71G | 0.03 | 97.78 | 93.93 | 48.97 |

Cy, cyproflanilide; Av, avermectin; Cl, chlorantraniliprole; ck, control group. CyM, midgut group under cyproflanilide stress

**Supplement Table 5 Information of 131 DEGs involved in detoxification (corresponding to data in figure 2)**

| No. | gene_id | log2  FoldChange | padj | gene_chr | gene_start | gene_end | gene_strand | gene_length | family |
| --- | --- | --- | --- | --- | --- | --- | --- | --- | --- |
| 39 up-regulated P450 members in the group of 2335 data set | | | | | | | | | |
| 1 | 118270778 | 2.76 | 2.19E-19 | Chr 14 | 7688680 | 7701440 | + | 3176 | p450 |
| 2 | 118264850 | 2.23 | 1.02E-17 | Chr 6 | 17573890 | 17592827 | + | 3434 | p450 |
| 3 | 118262727 | 1.64 | 4.90E-17 | Chr 4 | 9287201 | 9301883 | - | 2313 | p450 |
| 4 | 118273800 | 1.46 | 3.93E-12 | Chr 19 | 472282 | 491288 | - | 8595 | p450 |
| 5 | 118282331 | 4.34 | 1.12E-10 | Chr 3 | 4612465 | 4617755 | - | 1613 | p450 |
| 6 | 118272796 | 2.69 | 2.03E-10 | Chr 17 | 7797450 | 7803289 | + | 1697 | p450 |
| 7 | 118263357 | 1.25 | 1.25E-09 | Chr 5 | 11903979 | 11912812 | - | 2138 | p450 |
| 8 | 118266763 | 1.31 | 5.19E-09 | Chr 9 | 10510141 | 10514554 | - | 3794 | p450 |
| 9 | 118282431 | 3.12 | 3.32E-08 | Chr 3 | 2894492 | 2899311 | + | 1595 | p450 |
| 10 | 118263885 | 1.77 | 1.04E-06 | Chr 5 | 12050968 | 12061544 | - | 2139 | p450 |
| 11 | 118264635 | 1.45 | 2.79E-06 | Chr 6 | 16033571 | 16039759 | - | 1775 | p450 |
| 12 | 118270311 | 1.61 | 3.97E-06 | Chr 14 | 7463961 | 7473908 | + | 1693 | p450 |
| 13 | 118271683 | 3.04 | 7.72E-06 | Chr 16 | 8655739 | 8664742 | - | 1601 | p450 |
| 14 | 118275193 | 1.24 | 1.18E-05 | Chr 20 | 7094643 | 7105337 | + | 2328 | p450 |
| 15 | 118266961 | 1.36 | 1.26E-05 | Chr 9 | 7467397 | 7470685 | + | 2586 | p450 |
| 16 | 118262785 | 1.13 | 1.71E-05 | Chr 4 | 12194228 | 12197161 | + | 1799 | p450 |
| 17 | 118274175 | 1.07 | 2.57E-05 | Chr 19 | 10094933 | 10097438 | - | 1994 | p450 |
| 18 | 118281718 | 1.70 | 3.02E-05 | Un | 116251 | 127272 | + | 2247 | p450 |
| 19 | 118269078 | 1.70 | 7.72E-05 | Chr 12 | 17029657 | 17070242 | + | 2781 | p450 |
| 20 | 118282305 | 2.09 | 1.30E-04 | Chr 3 | 4324090 | 4329117 | + | 1662 | p450 |
| 21 | 118268617 | 1.10 | 2.08E-04 | Chr 11 | 5078869 | 5080443 | - | 1575 | p450 |
| 22 | 118275081 | 2.32 | 3.23E-04 | Chr 20 | 7059296 | 7084287 | + | 1998 | p450 |
| 23 | 118282370 | 1.94 | 3.57E-04 | Chr 3 | 7348613 | 7361295 | + | 1823 | p450 |
| 24 | 118270728 | 1.21 | 4.56E-04 | Chr 14 | 6487404 | 6500384 | - | 1822 | p450 |
| 25 | 118270763 | 2.74 | 5.89E-04 | Chr 14 | 362920 | 370231 | + | 1728 | p450 |
| 26 | 118263660 | 1.32 | 7.60E-04 | Chr 5 | 485203 | 495454 | + | 1697 | p450 |
| 27 | 118281824 | 3.19 | 9.77E-04 | Un | 70768 | 80091 | - | 1666 | p450 |
| 28 | 118270307 | 1.04 | 1.14E-03 | Chr 14 | 7548751 | 7560666 | - | 1830 | p450 |
| 29 | 118264058 | 1.50 | 2.58E-03 | Chr 6 | 17547308 | 17553466 | + | 1918 | p450 |
| 30 | 118264056 | 1.11 | 4.54E-03 | Chr 6 | 17553838 | 17558228 | + | 1904 | p450 |
| 31 | 118281717 | 1.82 | 5.52E-03 | Un | 92694 | 112830 | + | 1964 | p450 |
| 32 | 118279836 | 1.15 | 1.46E-02 | Chr 28 | 8157150 | 8159979 | - | 1824 | p450 |
| 33 | 118282336 | 1.10 | 1.49E-02 | Chr 3 | 4342276 | 4349008 | - | 2001 | p450 |
| 34 | 118279731 | 1.28 | 1.64E-02 | Chr 28 | 10143214 | 10147061 | - | 1835 | p450 |
| 35 | 118269105 | 1.37 | 1.77E-02 | Chr 12 | 10626124 | 10666837 | - | 2814 | p450 |
| 36 | 118264055 | 1.43 | 2.63E-02 | Chr 6 | 17525761 | 17535173 | + | 2057 | p450 |
| 37 | 118272240 | 1.03 | 2.78E-02 | Chr 16 | 8675276 | 8683030 | + | 1602 | p450 |
| 38 | 118270308 | 1.84 | 3.41E-02 | Chr 14 | 7403928 | 7419572 | - | 2743 | p450 |
| 39 | 118265346 | 1.65 | 3.91E-02 | Chr 7 | 6282410 | 6288684 | - | 1728 | p450 |
| 15 down-regulated P450 members in the group of 2335 data set in figure 2 | | | | | | | | | |
| 1 | 118278003 | -1.77 | 2.12E-12 | Chr 25 | 6402468 | 6424711 | - | 3545 | p450 |
| 2 | 118272349 | -1.13 | 6.43E-11 | Chr 17 | 9984286 | 9986990 | + | 2014 | p450 |
| 3 | 118274506 | -1.32 | 2.48E-10 | Chr 20 | 12631441 | 12633474 | + | 1739 | p450 |
| 4 | 118270312 | -1.29 | 2.29E-09 | Chr 14 | 7626406 | 7641574 | - | 1775 | p450 |
| 5 | 118271197 | -3.28 | 1.43E-07 | Chr 15 | 2291738 | 2294699 | - | 2505 | p450 |
| 6 | 118270758 | -2.12 | 3.12E-07 | Chr 14 | 6591849 | 6609775 | - | 1750 | p450 |
| 7 | 118281224 | -1.93 | 8.22E-07 | Chr 31 | 4601335 | 4609185 | - | 1932 | p450 |
| 8 | 118263077 | -2.04 | 3.22E-05 | Chr 4 | 12283962 | 12285850 | - | 1889 | p450 |
| 9 | 118263048 | -1.17 | 3.47E-05 | Chr 4 | 12280553 | 12282385 | + | 1833 | p450 |
| 10 | 118270350 | -1.58 | 5.46E-04 | Chr 14 | 6640451 | 6659733 | + | 2141 | p450 |
| 11 | 118281189 | -1.87 | 6.89E-04 | Chr 31 | 1196085 | 1203682 | + | 2894 | p450 |
| 12 | 118270860 | -1.04 | 1.65E-02 | Chr 14 | 7333139 | 7343484 | - | 1705 | p450 |
| 13 | 118274106 | -5.58 | 2.15E-02 | Chr 19 | 2081548 | 2094942 | + | 2063 | p450 |
| 14 | 118269113 | -2.45 | 3.08E-02 | Chr 12 | 2055472 | 2061510 | - | 2194 | p450 |
| 1 up-regulated P450 members in the group of 108 data set in figure 2 | | | | | | | | | |
| 1 | 118273911 | 2.21 | 5.08E-12 | Chr 19 | 6014934 | 6017890 | + | 2455 | p450 |
| 1 down-regulated P450 members in the group of 108 data set in figure 2 | | | | | | | | | |
| 1 | 118281490 | -1.57 | 1.70E-03 | Chr 3 | 203169 | 206842 | + | 3674 | p450 |
| 1 up-regulated GST members in the group of 2335 data set in figure 2 | | | | | | | | | |
| 1 | 118261724 | 1.62 | 1.11E-09 | Chr 3 | 8506416 | 8517854 | - | 2086 | GST |
| 7 down-regulated GST members in the group of 2335 data set in figure 2 | | | | | | | | | |
| 1 | 118271638 | -3.66 | 5.00E-05 | Chr 16 | 15510221 | 15513223 | + | 858 | GST |
| 2 | 118266003 | -1.06 | 6.56E-05 | Chr 8 | 6791297 | 6794352 | + | 1126 | GST |
| 3 | 118271634 | -2.00 | 9.63E-04 | Chr 16 | 158621 | 162734 | + | 1020 | GST |
| 4 | 118269546 | -1.06 | 3.67E-05 | Chr 13 | 12695055 | 12705389 | + | 1173 | GST |
| 5 | 118270030 | -3.14 | 4.38E-05 | Chr 13 | 16606258 | 16608873 | - | 1126 | GST |
| 6 | 118270149 | -1.24 | 5.39E-03 | Chr 13 | 15660665 | 15669376 | - | 1507 | GST |
| 7 | 118271633 | -1.40 | 3.81E-02 | Chr 16 | 150687 | 157479 | + | 981 | GST |
| 1 down-regulated GST members in the group of 108 data set in figure 2 | | | | | | | | | |
| 1 | 118269785 | 1.12 | 2.71E-02 | Chr 13 | 13410251 | 13421648 | - | 1028 | GST |
| 6 down-regulated GST members in the group of 103 data set in figure 2 | | | | | | | | | |
| 1 | 118277779 | -1.85 | 5.92E-04 | Chr 25 | 3836724 | 3842157 | + | 932 | GST |
| 2 | 118261929 | -1.34 | 3.89E-03 | Chr 3 | 8188368 | 8191022 | + | 910 | GST |
| 3 | 118277778 | -1.69 | 7.68E-03 | Chr 25 | 3744017 | 3748267 | + | 1130 | GST |
| 4 | 118266190 | -1.02 | 3.53E-02 | Chr 8 | 1573379 | 1575278 | + | 1444 | GST |
| 5 | 118270053 | -2.47 | 6.83E-03 | Chr 13 | 16610976 | 16614683 | - | 809 | GST |
| 6 | 118271636 | -1.03 | 3.62E-02 | Chr 16 | 15493888 | 15497382 | + | 1133 | GST |
| 5 up-regulated UGT members in the group of 2335 data set in figure 2 | | | | | | | | | |
| 1 | 118266879 | 1.46 | 1.77E-18 | Chr 9 | 5892284 | 5899900 | - | 2195 | UGT |
| 2 | 118279357 | 2.75 | 1.45E-09 | Chr 27 | 9844142 | 9847228 | - | 1811 | UGT |
| 3 | 118277902 | 1.17 | 2.96E-05 | Chr 25 | 4942876 | 4949872 | + | 1891 | UGT |
| 4 | 118277895 | 1.92 | 6.08E-03 | Chr 25 | 4838130 | 4843892 | + | 2057 | UGT |
| 5 | 118278231 | 1.06 | 2.91E-02 | Chr 25 | 4829952 | 4831438 | + | 726 | UGT |
| 10 down-regulated UGT members in the group of 2335 data set in figure 2 | | | | | | | | | |
| 1 | 118279153 | -2.95 | 1.88E-14 | Chr 27 | 9665913 | 9669720 | - | 1728 | UGT |
| 2 | 118269227 | -1.62 | 7.18E-09 | Chr 12 | 6066924 | 6070890 | + | 1787 | UGT |
| 3 | 118281901 | -3.03 | 1.14E-06 | Un | 135636 | 142684 | + | 2101 | UGT |
| 4 | 118277904 | -1.17 | 2.92E-06 | Chr 25 | 4877095 | 4890764 | + | 2381 | UGT |
| 5 | 118279191 | -1.47 | 1.07E-05 | Chr 27 | 9645234 | 9646186 | - | 953 | UGT |
| 6 | 118277768 | -1.13 | 2.94E-04 | Chr 25 | 3988755 | 4000652 | + | 1623 | UGT |
| 7 | 118269255 | -2.96 | 3.15E-03 | Chr 12 | 5235954 | 5239845 | + | 1704 | UGT |
| 8 | 118275940 | -2.15 | 1.15E-02 | Chr 21 | 5637192 | 5647509 | - | 2466 | UGT |
| 9 | 118278649 | -1.25 | 3.51E-02 | Chr 26 | 8164157 | 8173417 | + | 1557 | UGT |
| 10 | 118279417 | -1.82 | 4.15E-02 | Chr 27 | 9874431 | 9877094 | + | 1606 | UGT |
| 3 down-regulated UGT members in the group of 108 data set in figure 2 | | | | | | | | | |
| 1 | 118279155 | -2.32 | 5.51E-29 | Chr 27 | 9649899 | 9653994 | - | 1760 | UGT |
| 2 | 118279411 | -2.57 | 5.71E-12 | Chr 27 | 9856396 | 9860182 | + | 1876 | UGT |
| 3 | 118279190 | -1.22 | 1.56E-04 | Chr 27 | 9640702 | 9641722 | - | 1021 | UGT |
| 3 down-regulated UGT members in the group of 103 data set in figure 2 | | | | | | | | | |
| 1 | 118279189 | -1.45 | 1.31E-03 | Chr 27 | 9630010 | 9643920 | - | 2644 | UGT |
| 2 | 118278849 | -2.12 | 9.44E-03 | Chr 26 | 8123516 | 8135553 | - | 1717 | UGT |
| 3 | 118278039 | -1.26 | 3.12E-02 | Chr 25 | 4956854 | 4968438 | - | 3309 | UGT |
| 11 up-regulated COE members in the group of 2335 data set in figure 2 | | | | | | | | | |
| 1 | 118275677 | 1.90 | 2.06E-14 | Chr 21 | 2294340 | 2301648 | + | 2899 | COE |
| 2 | 118276152 | 1.49 | 4.13E-07 | Chr 22 | 890020 | 893562 | + | 1786 | COE |
| 3 | 118277744 | 1.01 | 2.02E-06 | Chr 2 | 16652348 | 16655317 | + | 2187 | COE |
| 4 | 118263041 | 1.00 | 4.07E-04 | Chr 4 | 14945342 | 14949860 | - | 1761 | COE |
| 5 | 118262722 | 1.99 | 2.02E-03 | Chr 4 | 14927775 | 14930778 | - | 1686 | COE |
| 6 | 118272089 | 1.97 | 3.17E-03 | Chr 16 | 5318986 | 5357757 | + | 2882 | COE |
| 7 | 118266127 | 1.51 | 1.18E-02 | Chr 8 | 1690926 | 1708144 | + | 3269 | COE |
| 8 | 118273464 | 1.04 | 2.32E-02 | Chr 18 | 8193217 | 8264517 | - | 5906 | COE |
| 9 | 118262827 | 1.75 | 2.61E-02 | Chr 4 | 10957954 | 11062443 | - | 4342 | COE |
| 10 | 118273142 | 1.25 | 2.65E-02 | Chr 18 | 3071018 | 3143667 | - | 3474 | COE |
| 11 | 118266364 | 1.41 | 3.75E-02 | Chr 8 | 1672906 | 1677288 | - | 2703 | COE |
| 13 down-regulated COE members in the group of 2335 data set in figure 2 | | | | | | | | | |
| 1 | 118276231 | -2.05 | 8.90E-22 | Chr 22 | 1564615 | 1569842 | - | 1730 | COE |
| 2 | 118275979 | -2.81 | 7.61E-12 | Chr 22 | 1573547 | 1582705 | - | 3431 | COE |
| 3 | 118269824 | -1.77 | 8.77E-05 | Chr 13 | 3480008 | 3488010 | + | 2207 | COE |
| 4 | 118275861 | -1.21 | 1.09E-04 | Chr 21 | 795939 | 798407 | - | 1753 | COE |
| 5 | 118275549 | -1.47 | 1.15E-04 | Chr 21 | 774941 | 784827 | - | 1906 | COE |
| 6 | 118276233 | -3.04 | 1.02E-03 | Chr 22 | 1552582 | 1555748 | - | 1973 | COE |
| 7 | 118269160 | -1.31 | 1.62E-03 | Chr 12 | 14879167 | 14892037 | + | 585 | COE |
| 8 | 118263588 | -1.17 | 3.75E-03 | Chr 5 | 8719258 | 8724517 | - | 3827 | COE |
| 9 | 118276232 | -2.85 | 4.05E-03 | Chr 22 | 1558655 | 1561578 | - | 1924 | COE |
| 10 | 118263045 | -1.06 | 4.68E-03 | Chr 4 | 14967542 | 14970465 | - | 2119 | COE |
| 11 | 118265387 | -2.80 | 7.87E-03 | Chr 7 | 9506581 | 9509023 | - | 2003 | COE |
| 12 | 118275552 | -1.59 | 1.28E-02 | Chr 21 | 777409 | 779469 | - | 1648 | COE |
| 13 | 118275668 | -3.14 | 1.73E-02 | Chr 21 | 6980951 | 7005381 | - | 2932 | COE |
| 2 down-regulated COE members in the group of 108 data set in figure 2 | | | | | | | | | |
| 1 | 118263044 | -2.11 | 4.13E-04 | Chr 4 | 14972061 | 14974098 | - | 1744 | COE |
| 2 | 118263046 | -1.10 | 2.18E-02 | Chr 4 | 14963660 | 14966713 | - | 1908 | COE |
| 1 up-regulated COE members in the group of 103 data set in figure 2 | | | | | | | | | |
| 1 | 118279115 | 3.35 | 5.00E-03 | Chr 27 | 6545428 | 6558854 | + | 3488 | COE |
| 1 down-regulated COE members in the group of 103 data set in figure 2 | | | | | | | | | |
| 1 | 118263047 | -1.17 | 3.20E-02 | Chr 4 | 14959233 | 14962353 | - | 1933 | COE |
| 6 up-regulated ABC members in the group of 2335 data set in figure 2 | | | | | | | | | |
| 1 | 118274703 | 1.98 | 5.74E-04 | Chr 20 | 2243512 | 2307883 | + | 6910 | ABC |
| 2 | 118278698 | 1.52 | 9.96E-03 | Chr 2 | 19614691 | 19635168 | - | 2329 | ABC |
| 3 | 118272465 | 1.20 | 1.95E-02 | Chr 17 | 9800108 | 9891456 | + | 4841 | ABC |
| 4 | 118270222 | 1.67 | 2.59E-02 | Chr 14 | 13532185 | 13592054 | + | 3936 | ABC |
| 5 | 118273834 | 1.39 | 3.10E-02 | Chr 2 | 17553234 | 17578022 | + | 2276 | ABC |
| 6 | 118265120 | 1.65 | 3.33E-04 | Chr 7 | 15507784 | 15551935 | + | 4837 | ABC |
| 5 down-regulated ABC members in the group of 2335 data set in figure 2 | | | | | | | | | |
| 1 | 118262348 | -1.13 | 5.36E-16 | Chr 4 | 1574893 | 1590688 | + | 3814 | ABC |
| 2 | 118267339 | -1.16 | 1.33E-11 | Chr 9 | 1784415 | 1794625 | + | 3146 | ABC |
| 3 | 118282367 | -1.13 | 2.20E-03 | Chr 3 | 14907519 | 14918167 | + | 2145 | ABC |
| 4 | 118267199 | -1.64 | 4.63E-06 | Chr 9 | 3606741 | 3629899 | - | 2336 | ABC |
| 5 | 118267197 | -1.39 | 6.52E-06 | Chr 9 | 3375633 | 3402566 | - | 2807 | ABC |

**Supplement Table 6 Information of 34 cuticle proteins (CPs) (corresponding to data in figure 2)**

| No. | gene_id | log2  FoldChange | padj | gene_chr | gene_start | gene_end | gene_strand | gene_length | family |
| --- | --- | --- | --- | --- | --- | --- | --- | --- | --- |
| 30 up-regulated CPs members in the group of 2335 data set in figure 2 | | | | | | | | | |
| 1 | 118282269 | 3.74 | 8.78E-52 | Chr 3 | 9293952 | 9309135 | + | 2040 | CP |
| 2 | 118273639 | 4.76 | 1.02E-44 | Chr 18 | 13082075 | 13082963 | - | 706 | CP |
| 3 | 118273466 | 4.32 | 1.92E-15 | Chr 18 | 13062307 | 13063511 | + | 835 | CP |
| 4 | 118282341 | 2.00 | 2.48E-10 | Chr 3 | 9765977 | 9769217 | + | 1176 | CP |
| 5 | 118262016 | 1.74 | 3.86E-10 | Chr 3 | 12688337 | 12690067 | - | 1491 | CP |
| 6 | 118261958 | 2.48 | 4.75E-10 | Chr 3 | 9596280 | 9601302 | - | 673 | CP |
| 7 | 118276814 | 1.43 | 1.37E-07 | Chr 2 | 13028389 | 13031758 | + | 1192 | CP |
| 8 | 118281347 | 1.70 | 1.70E-06 | Chr 3 | 12046162 | 12047886 | - | 1481 | CP |
| 9 | 118282288 | 5.57 | 1.78E-06 | Chr 3 | 9672763 | 9673925 | - | 605 | CP |
| 10 | 118273319 | 4.16 | 6.22E-06 | Chr 18 | 13072318 | 13073205 | + | 705 | CP |
| 11 | 118276210 | 1.67 | 2.16E-05 | Chr 2 | 13275421 | 13278829 | + | 1196 | CP |
| 12 | 118282330 | 7.34 | 2.31E-05 | Chr 3 | 9658582 | 9659971 | - | 1002 | CP |
| 13 | 118273654 | 7.35 | 6.54E-05 | Chr 18 | 13057227 | 13058027 | + | 615 | CP |
| 14 | 118282340 | 2.55 | 7.85E-05 | Chr 3 | 9759062 | 9764415 | - | 4659 | CP |
| 15 | 118273370 | 2.03 | 9.47E-05 | Chr 18 | 12994780 | 12995670 | - | 604 | CP |
| 16 | 118273653 | 3.37 | 2.87E-04 | Chr 18 | 13055812 | 13056963 | + | 1026 | CP |
| 17 | 118273465 | 4.54 | 3.42E-04 | Chr 18 | 13059259 | 13060424 | - | 846 | CP |
| 18 | 118273336 | 3.33 | 3.85E-04 | Chr 18 | 13079069 | 13079956 | + | 705 | CP |
| 19 | 118266485 | 1.22 | 4.04E-04 | Chr 8 | 13586328 | 13590545 | - | 915 | CP |
| 20 | 118282374 | 3.61 | 1.41E-03 | Chr 3 | 9613920 | 9615495 | + | 659 | CP |
| 21 | 118262071 | 2.84 | 1.46E-03 | Chr 3 | 12387276 | 12392179 | - | 1035 | CP |
| 22 | 118276778 | 5.54 | 5.15E-03 | Chr 23 | 10524138 | 10529686 | + | 1918 | CP |
| 23 | 118276841 | 3.07 | 5.54E-03 | Chr 23 | 10536951 | 10541291 | + | 957 | CP |
| 24 | 118263986 | 3.79 | 1.45E-02 | Chr 6 | 12309584 | 12311420 | - | 678 | CP |
| 25 | 118261848 | 2.71 | 1.52E-02 | Chr 3 | 9655089 | 9656046 | - | 673 | CP |
| 26 | 118261963 | 2.32 | 1.76E-02 | Chr 3 | 12260601 | 12265343 | - | 835 | CP |
| 27 | 118266524 | 2.73 | 2.44E-02 | Chr 8 | 13648281 | 13656373 | + | 829 | CP |
| 28 | 118277984 | 2.73 | 2.46E-02 | Chr 25 | 6516280 | 6517415 | + | 927 | CP |
| 29 | 118265846 | 3.20 | 2.99E-02 | Chr 8 | 13499053 | 13509869 | + | 1807 | CP |
| 30 | 118265573 | 2.64 | 3.37E-02 | Chr 7 | 246599 | 256674 | - | 1254 | CP |
| 4 down-regulated CPs members in the group of 2335 data set in figure 2 | | | | | | | | | |
| 1 | 118269443 | -1.52 | 6.85E-04 | Chr 12 | 12075294 | 12079187 | + | 639 | CP |
| 2 | 118269179 | -1.26 | 1.49E-02 | Chr 12 | 12060808 | 12064217 | + | 673 | CP |
| 3 | 118282151 | -1.13 | 1.52E-02 | Chr 3 | 9893431 | 9894377 | - | 717 | CP |
| 4 | 118282159 | -2.34 | 2.32E-02 | Chr 3 | 9900635 | 9902285 | - | 670 | CP |

**Supplement Table 7 KEGG pathway enrichment for DEGs of larvae (whole body) under cyproflanilide stress**

| No. | KEGG ID | Description | GeneRatio | Padj | Gene ID of DEGs |
| --- | --- | --- | --- | --- | --- |
| 1 | bmor03030 | DNA replication | 29/667 | 1.30E-12 | 118276661/118269327/118269774/118276817/118261842/118280862/118270792/118262491/118277548/118282057/118276084/118275939/118266401/118268916/118277869/118271258/118269633/118276101/118273079/118268917/118262492/118267086/118275632/118273848/118276818/118267087/118262316/118277500/118273376 |
| 2 | bmor03050 | Proteasome | 30/667 | 6.47E-10 | 118278553/118273500/118275905/118264598/118275405/118279178/118281880/118271650/118268860/118273178/118263013/118273449/118271668/118277121/118263428/118269022/118273101/118278603/novel.605/118274000/118277502/118271282/118279861/118267530/118267527/118281829/118272692/118271299/118263951/118263429 |
| 3 | bmor00830 | Retinol metabolism | 26/667 | 0.000257 | 118279155/118279153/118279411/118279357/118262815/118269227/118281901/118269979/118274246/118279191/118269957/118277894/118279190/118262993/118277768/118274079/118269255/118277895/118275940/118275233/118277339/118277340/118269538/118278649/118279417/118277935 |
| 4 | bmor03430 | Mismatch repair | 14/667 | 0.000277 | 118276661/118269327/118269774/118261842/118281754/118282057/118268916/118271258/118276101/118268917/118273848/118281706/118262316/118277500 |
| 5 | bmor03410 | Base excision repair | 13/667 | 0.000747 | 118263831/118276661/118269774/118276817/118261842/118280862/118277548/118269567/118275632/118273848/118276818/118274947/118277500 |
| 6 | bmor00860 | Porphyrin and chlorophyll metabolism | 21/667 | 0.001389 | 118279155/118279153/118279411/118279357/118269227/118269320/118275414/118281901/118276891/118279191/118276919/118276341/118279190/118277768/118269289/118279239/118269255/118277895/118275940/118278649/118279417 |
| 7 | bmor04141 | Protein processing in endoplasmic reticulum | 45/667 | 0.00305 | 118263256/118275759/118270230/118281274/118262976/118263300/118266323/118262575/118262163/118263319/118272540/118275235/118272081/118262609/118261831/118262403/118263561/118266311/118272400/118272650/118273177/118276613/118272926/118274154/118267118/118268793/118270739/118271897/118275936/118275263/118269717/118275460/118281809/118273244/118262141/118275131/118272539/118269710/118272987/118278608/118279023/118276394/118278622/118282000/118280750 |
| 8 | bmor03420 | Nucleotide excision repair | 17/667 | 0.012318 | 118276661/118269327/118269774/118276817/118261842/118280862/118268916/118274109/118271258/118276101/118268917/118279368/118275632/118273848/118276818/118262316/118277500 |
| 9 | bmor00981 | Insect hormone biosynthesis | 13/667 | 0.026156 | 118276057/118278003/118272796/118281224/118266961/118279176/118279351/118281189/118279322/118275209/118268832/118274846/118269113 |
| 10 | bmor00040 | Pentose and glucuronate interconversions | 19/667 | 0.029686 | 118279155/118279153/118279411/118264594/118279357/118269227/118269320/118281901/118279191/118268173/118279190/118282275/118277768/118274164/118269255/118277895/118275940/118278649/118279417 |
| 11 | bmor00030 | Pentose phosphate pathway | 13/667 | 0.029686 | 118274581/118273982/118274074/118274881/118263912/118264560/118263320/118279364/118273666/118277306/118277790/118268173/118271607 |
| 12 | bmor00790 | Folate biosynthesis | 14/667 | 0.038886 | 118280647/118280064/118277296/118269257/118268821/118274458/118273633/118266284/118276922/118275911/118273450/118262978/118263443/118280652 |
| 13 | bmor00350 | Tyrosine metabolism | 11/667 | 0.038886 | 118273421/118272070/118280997/118273572/118272996/118263443/118267010/118262964/118280999/118269176/118281154 |
| 14 | bmor01040 | Biosynthesis of unsaturated fatty acids | 19/667 | 0.051555 | 118264926/118272916/118262516/118264927/118264933/118282219/118261948/118264931/118261846/118261849/118266995/118280831/118270717/118282220/118262043/118270754/118261956/118262014/118261847 |
| 15 | bmor00510 | N-Glycan biosynthesis | 15/667 | 0.067015 | 118263300/118262609/118276613/118267118/118271897/118275936/118262706/118269717/118275460/118281809/118279034/118274450/118278608/118278542/118282000 |
| 16 | bmor00983 | Drug metabolism - other enzymes | 32/667 | 0.085526 | 118279155/118274075/118279153/118281586/118279411/118263464/118265403/118261724/118279357/118269227/118267768/118278241/118267764/118269320/118281901/118267765/118279191/118275970/118269546/118267728/118279190/118277768/118264713/118269255/118267776/118267793/118277895/118275940/118267950/118269785/118278649/118279417 |
| 17 | bmor03060 | Protein export | 10/667 | 0.16155 | 118263256/118262976/118262163/118273204/118268163/118263334/118274668/118262008/118263816/118272987 |
| 18 | bmor00980 | Metabolism of xenobiotics by cytochrome P450 | 24/667 | 0.237203 | 118279155/118279153/118279411/118280647/118261724/118279357/118269227/118281901/118273633/118279191/118269546/118271638/118279190/118277768/118273450/118271634/118269255/118277895/118275940/118269785/118280652/118278649/118271633/118279417 |
| 19 | bmor00053 | Ascorbate and aldarate metabolism | 15/667 | 0.280684 | 118279155/118279153/118279411/118279357/118269227/118281901/118279191/118279190/118277768/118269255/118277895/118275214/118275940/118278649/118279417 |
| 20 | bmor00260 | Glycine, serine and threonine metabolism | 14/667 | 0.288453 | 118268972/118271984/118275916/118268856/118276929/118281669/118268504/118271727/118278202/118274652/118263297/118273081/118272233/118272097 |

GeneRatio, means the ratio of the number of differential genes annotated to the KEGG pathway number to the total number of differential genes.

**Supplement Table 8 KEGG pathway enrichment for DEGs of larval midgut samples under cyproflanilide stress**

| No. | KEGG ID | Description | GeneRatio | Padj | Gene ID of DEGs |
| --- | --- | --- | --- | --- | --- |
| 1 | bmor00980 | Metabolism of xenobiotics by cytochrome P450 | 13/82 | 6.23E-06 | 118279155/118277779/118269785/118279189/118272158/118261929/118277778/118279411/118278849/118277029/118266190/118271636/118279190 |
| 2 | bmor00982 | Drug metabolism - cytochrome P450 | 11/82 | 4.33E-05 | 118279155/118277779/118269785/118279189/118261929/118277778/118279411/118278849/118266190/118271636/118279190 |
| 3 | bmor04141 | Protein processing in endoplasmic reticulum | 12/82 | 0.002348 | 118281274/118279023/118272987/118276394/118272400/118274207/118267118/118268792/118272540/118280733/118280747/118269145 |
| 4 | bmor00983 | Drug metabolism - other enzymes | 10/82 | 0.002348 | 118279155/118277779/118269785/118279189/118261929/118277778/118279411/118278849/118266190/118279190 |
| 5 | bmor00040 | Pentose and glucuronate interconversions | 7/82 | 0.002348 | 118279155/118279189/118272158/118279411/118278849/118277029/118279190 |
| 6 | bmor00830 | Retinol metabolism | 7/82 | 0.003114 | 118279155/118279189/118277894/118269538/118279411/118278849/118279190 |
| 7 | bmor00053 | Ascorbate and aldarate metabolism | 6/82 | 0.008391 | 118279155/118279189/118268491/118279411/118278849/118279190 |
| 8 | bmor00270 | Cysteine and methionine metabolism | 5/82 | 0.023048 | 118268701/118272480/118268145/118277020/118280096 |
| 9 | bmor00860 | Porphyrin and chlorophyll metabolism | 5/82 | 0.032764 | 118279155/118279189/118279411/118278849/118279190 |
| 10 | bmor00010 | Glycolysis / Gluconeogenesis | 5/82 | 0.032764 | 118269393/118272480/118277526/118268491/118271607 |
| 11 | bmor00330 | Arginine and proline metabolism | 4/82 | 0.058423 | 118273671/118269617/118268491/118273944 |
| 12 | bmor00620 | Pyruvate metabolism | 4/82 | 0.058423 | 118272480/118277526/118269578/118268491 |
| 13 | bmor00480 | Glutathione metabolism | 6/82 | 0.093593 | 118277779/118269785/118261929/118277778/118266190/118271636 |
| 14 | bmor01230 | Biosynthesis of amino acids | 5/82 | 0.126078 | 118268701/118268972/118268145/118277790/118277020 |
| 15 | bmor00030 | Pentose phosphate pathway | 3/82 | 0.133318 | 118277790/118274581/118271607 |
| 16 | bmor00500 | Starch and sucrose metabolism | 3/82 | 0.172478 | 118273028/118268996/118271607 |
| 17 | bmor00770 | Pantothenate and CoA biosynthesis | 2/82 | 0.215738 | 118279354/118274957 |
| 18 | bmor00100 | Steroid biosynthesis | 3/82 | 0.215738 | 118280738/118271187/118272789 |
| 19 | bmor00760 | Nicotinate and nicotinamide metabolism | 2/82 | 0.283674 | 118280033/118272792 |
| 20 | bmor00260 | Glycine, serine and threonine metabolism | 3/82 | 0.286605 | 118268972/118277020/118263297 |

GeneRatio, means the ratio of the number of differential genes annotated to the KEGG pathway number to the total number of differential genes.

**Supplement Table 9 Expression profile of GABA receptor candidates of larvae treated with cyproflanilide**

| Gene_id | Whole body | | Midgut | | | Gene description^＆^ |
| --- | --- | --- | --- | --- | --- | --- |
|  | log_2_(F) | padj | | log_2_(F) | padj |  |
| 118262401^﹟^ | 1.30 | <0.01 | | 1.01 | 0.17 | GABA(B) receptor subunit 1-like |
| 118280690^﹟^ | 1.32 | <0.01 | | 1.04 | 1.00 | GABA(B) receptor subunit 2-like |
| 118265570 | 0.74 | 0.41 | | 1.63 | 1.00 | GABA(B) receptor subunit 2-like |
| 118265550 | 0.73 | 0.28 | | 0.36 | 0.84 | GABA(B) receptor subunit 2-like |
| 118277195^﹟^ | 1.14 | <0.01 | | -0.15 | 1.00 | GABA(A) receptor subunit beta-like |
| 118263634^﹟^ | 1.12 | 0.01 | | -1.70 | 1.00 | GABA(A) receptor subunit beta-like |
| 118273029^﹟^ | 0.99 | 0.01 | | 1.07 | 1.00 | GABA(A) receptor subunit beta-like |
| 118263641 | 1.67 | 1.00 | | / | / | GABA(A) receptor alpha-like |
| 118263649 | 2.54 | 1.00 | | / | / | GABA(A) receptor subunit beta-like |
| 118263650 | 1.99 | 1.00 | | / | / | GABA(A) receptor subunit beta-like |
| 118267290 | 0.60 | 0.38 | | -1.78 | 1.00 | GABA(A) receptor subunit delta-like |
| 118266945 | -0.15 | 0.72 | | 0.04 | 1.00 | GABA(A) receptor subunit delta-like |

﹟ belongs to DEGs; ＆, according to the genome annotations of *Spodoptera frugiperda* (Xiao et al., 2020)

**Supplement Table 10 P450 genes which were descripted in the annotation files of FAW genome (Xiao et al. 2020)**

| No. | P450 GeneID | Chr NO. | Start | Stop | Strand | Protein Product | Length  (aa) | BlastP (Expect value≤1.0E-100)  (select the first alignments target) | | Amino acid seq.identities (%) |
| --- | --- | --- | --- | --- | --- | --- | --- | --- | --- | --- |
|  |  |  |  |  |  |  |  | **Protein Name**  **(Xiao et al., 2020)** | **Protein Name**  **(Gouin et al., 2017)** |  |
| 1 | 118282213 | Chr 1 | 1407447 | 1409510 | + | XP_035459074.1 | 500 | probable P450 303a1 | GSSPFG00014994001.3-PA gene=CYP303A1 | 98 |
| 2 | 118266520 | Chr 1 | 10567822 | 10591252 | - | XP_035435890.1 | 489 | probable P450 305a1 isoform X1 | GSSPFG00029631001.2-PA gene=CYP305B1 fragment3 | 100 |
|  |  | Chr 1 | 10567822 | 10590868 | - | XP_035435898.1 | 463 | probable P450 305a1 isoform X2 | GSSPFG00029631001.2-PA gene=CYP305B1 fragment3 | 100 |
| 3 | 118281490 | Chr 3 | 203225 | 204724 | + | XP_035457962.1 | 499 | P450 6B5-like | GSSPFG00004596001.2-PA gene=CYP321A15 | 98 |
| 4 | 118282199 | Chr 3 | 2251398 | 2252906 | + | XP_035459052.1 | 502 | P450 6B5-like | GSSPFG00004596001.2-PA gene=CYP321A15 | 98 |
| 5 | 118282431 | Chr 3 | 2894548 | 2899278 | + | XP_035459401.1 | 501 | P450 4d2-like | GSSPFG00033314001-PA gene=GSSPFG00033314001 | 99 |
| 6 | 118262005 | Chr 3 | 3125754 | 3130654 | + | XP_035428980.1 | 505 | P450 4V2-like | GSSPFG00000132001-PA gene=GSSPFG00000132001 | 91 |
| 7 | 118262004 | Chr 3 | 3132971 | 3140488 | + | XP_035428979.1 | 508 | P450 4V2-like | GSSPFG00000132001-PA gene=GSSPFG00000132001 | 97 |
| 8 | 118261999 | Chr 3 | 3146019 | 3163985 | + | XP_035428975.1 | 501 | P450 4d2-like | GSSPFG00000135001-PA gene=GSSPFG00000135001 | 56 |
| 9 | 118282316 | Chr 3 | 3168650 | 3174969 | + | XP_035459231.1 | 504 | P450 4C1-like | GSSPFG00000135001-PA gene=GSSPFG00000135001 | 99 |
| 10 | 118282408 | Chr 3 | 3180791 | 3187816 | + | XP_035459368.1 | 505 | P450 4V2-like | GSSPFG00000135001-PA gene=GSSPFG00000135001 | 51 |
| 11 | 118282305 | Chr 3 | 4324099 | 4328979 | + | XP_035459218.1 | 504 | P450 4C1-like | GSSPFG00000135001-PA gene=GSSPFG00000135001 | 53 |
| 12 | 118282370 | Chr 3 | 7348613 | 7361158 | + | XP_035459320.1 | 511 | P450 4d2-like isoform X1 | GSSPFG00007240001.2-PA gene=CY341A11 partial_fragment1 | 97 |
|  |  | Chr 3 | 7353569 | 7361158 | + | XP_035459321.1 | 457 | P450 4C1-like isoform X2 | GSSPFG00007240001.2-PA gene=CY341A11 partial_fragment1 | 97 |
| 13 | 118282379 | Chr 3 | 3188727 | 3194289 | - | XP_035459330.1 | 507 | P450 4C1-like | GSSPFG00000135001-PA gene=GSSPFG00000135001 | 52 |
| 14 | 118282416 | Chr 3 | 4330007 | 4337545 | - | XP_035459383.1 | 505 | P450 4V2-like | GSSPFG00000135001-PA gene=GSSPFG00000135001 | 51 |
| 15 | 118282336 | Chr 3 | 4342618 | 4348864 | - | XP_035459267.1 | 504 | P450 4C1-like | GSSPFG00000135001-PA gene=GSSPFG00000135001 | 99 |
| 16 | 118282337 | Chr 3 | 4353213 | 4364667 | - | XP_035459268.1 | 501 | P450 4d2-like | GSSPFG00000135001-PA gene=GSSPFG00000135001 | 56 |
| 17 | 118282334 | Chr 3 | 4368482 | 4374160 | - | XP_035459264.1 | 508 | P450 4V2-like | GSSPFG00000132001-PA gene=GSSPFG00000132001 | 98 |
| 18 | 118282338 | Chr 3 | 4376285 | 4382364 | - | XP_035459269.1 | 508 | P450 4V2-like | GSSPFG00000132001-PA gene=GSSPFG00000132001 | 92 |
| 19 | 118282335 | Chr 3 | 4384439 | 4389770 | - | XP_035459265.1 | 505 | P450 4V2-like | GSSPFG00000132001-PA gene=GSSPFG00000132001 | 92 |
| 20 | 118282331 | Chr 3 | 4612488 | 4617671 | - | XP_035459258.1 | 501 | P450 4d2-like | GSSPFG00033314001-PA gene=GSSPFG00033314001 | 99 |
| 21 | 118261893 | Chr 3 | 6389236 | 6392478 | - | XP_035428809.1 | 517 | P450 6B6-like | GSSPFG00009831001.2-PA gene=CYP6AN4 | 99 |
| 22 | 118263012 | Chr 4 | 313794 | 322266 | + | XP_035430637.1 | 503 | P450 6l1-like | / | / |
| 23 | 118263002 | Chr 4 | 8998733 | 9001335 | + | XP_035430621.1 | 492 | P450 6B5-like | GSSPFG00035913001.2-PA gene=CYP337B5_partial | 99 |
| 24 | 118262642 | Chr 4 | 9087629 | 9089116 | + | XP_035430063.1 | 495 | P450 6B6-like | GSSPFG00015195001.2-PA gene=CYP321B3 | 98 |
| 25 | 118262785 | Chr 4 | 12194296 | 12196909 | + | XP_035430276.1 | 492 | P450 6B5-like | GSSPFG00035913001.2-PA gene=CYP337B5_partial | 99 |
| 26 | 118263048 | Chr 4 | 12280620 | 12282107 | + | XP_035430699.1 | 495 | P450 6B6-like | GSSPFG00015195001.2-PA gene=CYP321B3 | 98 |
| 27 | 118263010 | Chr 4 | 608596 | 616968 | - | XP_035430635.1 | 503 | P450 6l1-like | / | / |
| 28 | 118263005 | Chr 4 | 9075171 | 9076658 | - | XP_035430624.1 | 495 | P450 6B6-like | GSSPFG00015622001.2-PA gene=CYP321B1 | 96 |
| 29 | 118263083 | Chr 4 | 9091783 | 9093273 | - | XP_035430760.1 | 496 | P450 6k1-like | GSSPFG00035221001.3-PA gene=CYP321B4 | 99 |
| 30 | 118262727 | Chr 4 | 9287764 | 9297453 | - | XP_035430189.1 | 531 | P450 9e2-like | GSSPFG00008268001.2-PA gene=CYP9A30 | 77 |
|  |  | Chr 4 | 9287764 | 9297453 | - | XP_035430190.1 | 531 |  |  |  |
| 31 | 118262947 | Chr 4 | 12268094 | 12269749 | - | XP_035430547.1 | 551 | P450 6B6-like | GSSPFG00015622001.2-PA gene=CYP321B1 | 96 |
| 32 | 118263077 | Chr 4 | 12284240 | 12285730 | - | XP_035430751.1 | 496 | P450 6k1-like | GSSPFG00035221001.3-PA gene=CYP321B4 | 99 |
| 33 | 118263659 | Chr 5 | 477527 | 483717 | + | XP_035431664.1 | 508 | P450 CYP12A2-like | GSSPFG00002699001.4-PA gene=GSSPFG00002699001.4 | 98 |
| 34 | 118263660 | Chr 5 | 485365 | 495425 | + | XP_035431665.1 | 501 | P450 CYP12A2-like | GSSPFG00002700001.1-PA gene=GSSPFG00002700001.1 | 98 |
| 35 | 118263397 | Chr 5 | 6755788 | 6761402 | + | XP_035431241.1 | 493 | P450 4d2-like | GSSPFG00002703001.3-PA gene=CYP4L13 | 94 |
| 36 | 118263399 | Chr 5 | 6763154 | 6773150 | + | XP_035431242.1 | 493 | P450 4d2-like | GSSPFG00002703001.3-PA gene=CYP4L13 | 78 |
| 37 | 118263400 | Chr 5 | 6775047 | 6790924 | + | XP_035431243.1 | 492 | P450 4d2-like | GSSPFG00002703001.3-PA gene=CYP4L13 | 75 |
| 38 | 118263350 | Chr 5 | 12869108 | 12875682 | + | XP_035431171.1 | 571 | P450 4g15-like | GSSPFG00000960001.1-PA gene=GSSPFG00000960001.1 | 98 |
| 39 | 118263457 | Chr 5 | 12880710 | 12885872 | + | XP_035431368.1 | 563 | P450 4g15-like | GSSPFG00000960001.1-PA gene=GSSPFG00000960001.1 | 100 |
|  |  | Chr 5 | 12880710 | 12885872 | + | XP_035431369.1 | 563 |  |  |  |
| 40 | 118263448 | Chr 5 | 12892758 | 12901735 | + | XP_035431348.1 | 566 | P450 4g15-like | GSSPFG00031555001.1-PA gene=GSSPFG00031555001.1 | 83 |
| 41 | 118263554 | Chr 5 | 541614 | 558326 | - | XP_035431513.1 | 492 | P450 4d2-like | GSSPFG00002703001.3-PA gene=CYP4L13 | 75 |
| 42 | 118263555 | Chr 5 | 560106 | 569185 | - | XP_035431514.1 | 493 | P450 4d2-like | GSSPFG00002703001.3-PA gene=CYP4L13 | 85 |
| 43 | 118263710 | Chr 5 | 570831 | 574616 | - | XP_035431755.1 | 416 | P450 4d2-like | GSSPFG00002703001.3-PA gene=CYP4L13 | 97 |
| 44 | 118263547 | Chr 5 | 6834693 | 6845340 | - | XP_035431502.1 | 501 | P450 CYP12A2-like | GSSPFG00002700001.1-PA gene=GSSPFG00002700001.1 | 98 |
| 45 | 118263546 | Chr 5 | 6846999 | 6853150 | - | XP_035431501.1 | 508 | P450 CYP12A2-like | GSSPFG00002699001.4-PA gene=GSSPFG00002699001.4 | 98 |
| 46 | 118263357 | Chr 5 | 11904295 | 11909076 | - | XP_035431180.1 | 557 | P450 4g15-like | GSSPFG00026573001.4-PA gene=CYP4G75 | 99 |
| 47 | 118263885 | Chr 5 | 12051284 | 12056065 | - | XP_035431989.1 | 557 | P450 4g15-like | GSSPFG00026573001.4-PA gene=CYP4G75 | 99 |
| 48 | 118264057 | Chr 6 | 17220270 | 17223131 | + | XP_035432292.1 | 530 | P450 9e2-like | GSSPFG00003574001.4-PA gene=CYP9A26 partial | 98 |
| 49 | 118264059 | Chr 6 | 17433295 | 17438878 | + | XP_035432294.1 | 530 | P450 9e2-like | GSSPFG00024476001.2-PA gene=CYP9A58 | 55 |
| 50 | 118264055 | Chr 6 | 17526364 | 17534965 | + | XP_035432290.1 | 531 | P450 9e2-like | GSSPFG00008268001.2-PA gene=CYP9A30 | 98 |
| 51 | 118264058 | Chr 6 | 17550174 | 17553297 | + | XP_035432293.1 | 528 | P450 9e2-like | GSSPFG00024476001.2-PA gene=CYP9A58 | 68 |
| 52 | 118264056 | Chr 6 | 17555151 | 17558073 | + | XP_035432291.1 | 530 | P450 9e2-like | GSSPFG00003574001.4-PA gene=CYP9A26 partial | 97 |
| 53 | 118264788 | Chr 6 | 15682817 | 15697633 | - | XP_035433316.1 | 513 | P450 9e2-like | GSSPFG00026919001.2-PA gene=CYP9AJ1 partial | 98 |
| 54 | 118264635 | Chr 6 | 16033783 | 16039675 | - | XP_035433105.1 | 492 | P450 9e2-like | GSSPFG00008208001.5-PA gene=CYP9G17 partial | 99 |
| 55 | 118264410 | Chr 6 | 17175310 | 17182324 | - | XP_035432794.1 | 530 | P450 9e2-like | GSSPFG00024476001.2-PA gene=CYP9A58 | 55 |
|  |  | Chr 6 | 17175310 | 17182324 | - | XP_035432795.1 | 530 |  |  |  |
| 56 | 118264054 | Chr 6 | 17499432 | 17506021 | - | XP_035432287.1 | 574 | P450 9e2-like isoform X1 | GSSPFG00024476001.2-PA gene=CYP9A58 | 55 |
|  |  | Chr 6 | 17499432 | 17505432 | - | XP_035432289.1 | 530 | P450 9e2-like isoform X2 | GSSPFG00024476001.2-PA gene=CYP9A58 | 55 |
| 57 | 118265371 | Chr 7 | 4269709 | 4276240 | - | XP_035434100.1 | 465 | P450 6j1-like | GSSPFG00018669001.2-PB gene=CYP338A1 | 99 |
| 58 | 118265346 | Chr 7 | 6282695 | 6288639 | - | XP_035434067.1 | 465 | P450 6j1-like | GSSPFG00018669001.2-PB gene=CYP338A1 | 99 |
| 59 | 118265141 | Chr 7 | 15247798 | 15269660 | - | XP_035433755.1 | 1025 | P450 709B2-like | GSSPFG00022863001.2-PA gene=CYP4CG16 | 96 |
| 60 | 118265707 | Chr 8 | 5598416 | 5602442 | + | XP_035434678.1 | 505 | probable P450 49a1 | GSSPFG00035665001.3-PA gene=GSSPFG00035665001.3 | 93 |
|  |  | Chr 8 | 5598416 | 5602442 | + | XP_035434679.1 | 505 |  |  |  |
| 61 | 118266312 | Chr 8 | 5537815 | 5541880 | - | XP_035435611.1 | 505 | probable P450 49a1 | GSSPFG00035665001.3-PA gene=GSSPFG00035665001.3 | 92 |
| 62 | 118267194 | Chr 9 | 7403380 | 7405946 | + | XP_035436925.1 | 518 | P450 18a1-like | GSSPFG00000264001.3-PA gene=GSSPFG00000264001.3 | 83 |
| 63 | 118266961 | Chr 9 | 7467584 | 7469900 | + | XP_035436527.1 | 537 | P450 18a1-like | GSSPFG00024856001.2-PB gene=CYP18A1 | 98 |
| 64 | 118266714 | Chr 9 | 7471626 | 7481100 | - | XP_035436111.1 | 543 | P450 306a1-like isoform X1 | GSSPFG00016125001.1-PA gene=GSSPFG00016125001.1 | 99 |
|  |  | Chr 9 | 7471626 | 7477419 | - | XP_035436113.1 | 539 | P450 306a1-like isoform X2 | GSSPFG00016125001.1-PA gene=GSSPFG00016125001.1 | 99 |
|  |  | Chr 9 | 7471626 | 7477419 | - | XP_035436114.1 | 539 |  |  |  |
|  |  | Chr 9 | 7471626 | 7477419 | - | XP_035436115.1 | 539 |  |  |  |
|  |  | Chr 9 | 7471626 | 7477419 | - | XP_035436116.1 | 539 |  |  |  |
| 65 | 118266763 | Chr 9 | 10512149 | 10514256 | - | XP_035436192.1 | 495 | P450 6a2-like | GSSPFG00009765001.2-PA gene=CYP6AW1 partial | 100 |
| 66 | 118267855 | Chr 10 | 1131678 | 1133720 | + | XP_035437993.1 | 516 | P450 6B4-like | GSSPFG00021543001.2-PA gene=CYP3097A1 | 93 |
| 67 | 118267996 | Chr 10 | 4422697 | 4426447 | - | XP_035438156.1 | 579 | probable P450 301a1, mitochondrial | GSSPFG00006987001.2-PA gene=CYP339A1 | 99 |
| 68 | 118268506 | Chr 11 | 5062999 | 5064492 | + | XP_035438923.1 | 497 | P450 6B5-like | GSSPFG00008047001.2-PA gene=CYP321A9V2 | 98 |
| 69 | 118268618 | Chr 11 | 5069545 | 5071032 | + | XP_035439064.1 | 495 | probable P450 6a13 | GSSPFG00008048001.3-PA gene=Cytochrome P450 321A7 | 98 |
| 70 | 118268549 | Chr 11 | 5478125 | 5479618 | + | XP_035438979.1 | 497 | P450 6B5-like | GSSPFG00008047001.2-PA gene=CYP321A9V2 | 81 |
| 71 | 118268605 | Chr 11 | 5483490 | 5484980 | + | XP_035439042.1 | 496 | probable P450 6a13 | GSSPFG00008050001.2-PB gene=CYP321A8 | 98 |
| 72 | 118268604 | Chr 11 | 5073026 | 5074516 | - | XP_035439041.1 | 496 | probable P450 6a13 | GSSPFG00008050001.2-PB gene=CYP321A8 | 99 |
| 73 | 118268617 | Chr 11 | 5078888 | 5080381 | - | XP_035439063.1 | 497 | P450 6B5-like | GSSPFG00008047001.2-PA gene=CYP321A9V2 | 82 |
| 74 | 118268527 | Chr 11 | 5487259 | 5488746 | - | XP_035438952.1 | 495 | probable P450 6a13 | GSSPFG00008048001.3-PA gene=Cytochrome P450 321A7 | 99 |
| 75 | 118268486 | Chr 11 | 5493285 | 5494778 | - | XP_035438897.1 | 497 | P450 6B5-like | GSSPFG00008047001.2-PA gene=CYP321A9V2 | 97 |
| 76 | 118268370 | Chr 11 | 12224582 | 12252847 | - | XP_035438725.1 | 511 | probable P450 304a1 | GSSPFG00013052001-PA gene=GSSPFG00013052001 | 99 |
| 77 | 118268212 | Chr 11 | 12971230 | 13008181 | - | XP_035438483.1 | 511 | probable P450 304a1 | GSSPFG00013052001-PA gene=GSSPFG00013052001 | 98 |
| 78 | 118268718 | Chr 12 | 3794401 | 3802325 | + | XP_035439212.1 | 518 | P450 9e2-like | GSSPFG00016046001.2-PA gene=CYP354A14 partial | 89 |
| 79 | 118269181 | Chr 12 | 4026647 | 4034546 | + | XP_035440045.1 | 518 | P450 9e2-like | GSSPFG00016046001.2-PA gene=CYP354A14 partial | 89 |
| 80 | 118268818 | Chr 12 | 4638852 | 4642074 | + | XP_035439389.1 | 510 | P450 12b1, mitochondrial-like | GSSPFG00035934001.2-PA gene=CYP333A12 partial | 99 |
| 81 | 118269078 | Chr 12 | 17029867 | 17069294 | + | XP_035439893.1 | 540 | probable P450 49a1 | GSSPFG00016404001.2-PA gene=CYP428A1V1 | 79 |
| 82 | 118269113 | Chr 12 | 2055759 | 2060365 | - | XP_035439929.1 | 485 | P450 315a1, mitochondrial-like | GSSPFG00023669001.3-PA gene=CYP315A1 partial | 99 |
| 83 | 118269105 | Chr 12 | 10627105 | 10666627 | - | XP_035439922.1 | 540 | probable P450 49a1 | GSSPFG00016404001.2-PA gene=CYP428A1V1 | 78 |
| 84 | 118270822 | Chr 14 | 145601 | 151180 | + | XP_035442499.1 | 450 | P450 4C1-like | GSSPFG00005210001-PA gene=GSSPFG00005210001 | 97 |
| 85 | 118270695 | Chr 14 | 154025 | 158299 | + | XP_035442327.1 | 501 | P450 4C1-like | GSSPFG00005208001-PA gene=GSSPFG00005208001 | 97 |
| 86 | 118270694 | Chr 14 | 159440 | 165813 | + | XP_035442325.1 | 501 | P450 4V2-like isoform X1 | GSSPFG00005208001-PA gene=GSSPFG00005208001 | 88 |
|  |  | Chr 14 | 159440 | 165813 | + | XP_035442326.1 | 471 | P450 4V2-like isoform X2 | GSSPFG00030289001.2-PA gene=CYP421B1 | 65 |
| 87 | 118270823 | Chr 14 | 167531 | 173175 | + | XP_035442500.1 | 455 | P450 4C1-like | GSSPFG00030289001.2-PA gene=CYP421B1 | 96 |
| 88 | 118270750 | Chr 14 | 351166 | 361560 | + | XP_035442404.1 | 492 | P450 4c3-like | GSSPFG00005210001-PA gene=GSSPFG00005210001 | 41 |
| 89 | 118270763 | Chr 14 | 363162 | 370218 | + | XP_035442424.1 | 490 | P450 4c21-like | GSSPFG00005210001-PA gene=GSSPFG00005210001 | 43 |
| 90 | 118270458 | Chr 14 | 6373444 | 6384094 | + | XP_035441942.1 | 492 | P450 4c21-like | GSSPFG00027679001-PA gene=GSSPFG00027679001 | 100 |
| 91 | 118270459 | Chr 14 | 6391336 | 6396807 | + | XP_035441943.1 | 487 | P450 4C1-like | GSSPFG00031119001.2-PA gene=CYP340AB1 | 45 |
| 92 | 118270460 | Chr 14 | 6400328 | 6416385 | + | XP_035441944.1 | 487 | P450 4V2-like isoform X1 | GSSPFG00020736001.2-PA gene=CYP340L20 | 96 |
|  |  | Chr 14 | 6400328 | 6416385 | + | XP_035441945.1 | 487 | P450 4V2-like isoform X2 | GSSPFG00020736001.2-PA gene=CYP340L20 | 96 |
|  |  | Chr 14 | 6412860 | 6416385 | + | XP_035441946.1 | 388 | P450 4C1-like isoform X3 | GSSPFG00020736001.2-PA gene=CYP340L20 | 96 |
| 93 | 118270855 | Chr 14 | 6435522 | 6447850 | + | XP_035442532.1 | 535 | P450 4C1-like | GSSPFG00035919001.2-PA gene=CYP340L4 | 60 |
| 94 | 118270644 | Chr 14 | 6448644 | 6465444 | + | XP_035442275.1 | 479 | P450 4C1-like isoform X2 | GSSPFG00001900001.2-PA gene=CYP340L unknown_fragment3 | 79 |
|  |  | Chr 14 | 6449342 | 6465444 | + | XP_035442276.1 | 440 | P450 4C1-like isoform X3 | GSSPFG00001900001.2-PA gene=CYP340L unknown_fragment3 | 79 |
|  |  | Chr 14 | 6459286 | 6465444 | + | XP_035442274.1 | 494 | P450 4C1-like isoform X1 | GSSPFG00001900001.2-PA gene=CYP340L unknown_fragment3 | 78 |
| 95 | 118270856 | Chr 14 | 6467163 | 6470616 | + | XP_035442533.1 | 388 | P450 4C1-like | GSSPFG00001900001.2-PA gene=CYP340L unknown_fragment3 | 83 |
| 96 | 118270857 | Chr 14 | 6471029 | 6476023 | + | XP_035442534.1 | 494 | P450 4C1-like | GSSPFG00001900001.2-PA gene=CYP340L unknown_fragment3 | 80 |
| 97 | 118270645 | Chr 14 | 6481220 | 6485957 | + | XP_035442277.1 | 486 | P450 4C1-like | GSSPFG00035919001.2-PA gene=CYP340L4 | 84 |
| 98 | 118270858 | Chr 14 | 6513347 | 6520222 | + | XP_035442535.1 | 257 | P450 4c21-like | / | / |
| 99 | 118270350 | Chr 14 | 6640616 | 6659248 | + | XP_035441808.1 | 496 | P450 4C1-like | GSSPFG00027168001.2-PA gene=CYP340AD3 partial | 99 |
| 100 | 118270797 | Chr 14 | 6664937 | 6677192 | + | XP_035442467.1 | 488 | P450 4C1-like | GSSPFG00027152001.1-PA gene=GSSPFG00027152001.1 | 98 |
| 101 | 118270311 | Chr 14 | 7464136 | 7473854 | + | XP_035441738.1 | 487 | P450 4d1-like isoform X1 | GSSPFG00031882001-PA gene=GSSPFG00031882001 | 66 |
|  |  | Chr 14 | 7464136 | 7473854 | + | XP_035441739.1 | 450 | P450 4C1-like isoform X2 | GSSPFG00031882001-PA gene=GSSPFG00031882001 | 62 |
| 102 | 118270304 | Chr 14 | 7480699 | 7486522 | + | XP_035441727.1 | 500 | P450 4C1-like isoform X1 | GSSPFG00017290001.2-PB gene=CYP340L16C | 91 |
|  |  | Chr 14 | 7480699 | 7486522 | + | XP_035441728.1 | 496 | P450 4C1-like isoform X2 | GSSPFG00017290001.2-PB gene=CYP340L16C | 90 |
| 103 | 118270314 | Chr 14 | 7510721 | 7513968 | + | XP_035441743.1 | 373 | P450 4C1-like | GSSPFG00014033001.2-PA gene=CYP340L1 fragment3 | 57 |
|  |  | Chr 14 | 7510721 | 7513968 | + | XP_035441744.1 | 373 | P450 4C1-like | GSSPFG00014033001.2-PA gene=CYP340L1 fragment3 | 57 |
| 104 | 118270305 | Chr 14 | 7521673 | 7532630 | + | XP_035441730.1 | 482 | P450 4C1-like isoform X2 | GSSPFG00001900001.2-PA gene=CYP340L unknown_fragment3 | 82 |
|  |  | Chr 14 | 7521673 | 7532630 | + | XP_035441731.1 | 482 | P450 4C1-like isoform X3 | GSSPFG00001900001.2-PA gene=CYP340L unknown_fragment3 | 77 |
|  |  | Chr 14 | 7521673 | 7532630 | + | XP_035441732.1 | 482 | P450 4C1-like isoform X4 | GSSPFG00001900001.2-PA gene=CYP340L unknown_fragment3 | 79 |
|  |  | Chr 14 | 7527640 | 7532630 | + | XP_035441729.1 | 494 | P450 4C1-like isoform X1 | GSSPFG00001900001.2-PA gene=CYP340Lunknown_fragment3 | 76 |
| 105 | 118270864 | Chr 14 | 7533213 | 7538510 | + | XP_035442541.1 | 494 | P450 4C1-like | GSSPFG00001900001.2-PA gene=CYP340L unknown_fragment3 | 77 |
| 106 | 118270865 | Chr 14 | 7544800 | 7547349 | + | XP_035442542.1 | 320 | P450 4C1-like | GSSPFG00035919001.2-PA gene=CYP340L4 | 82 |
| 107 | 118270315 | Chr 14 | 7580835 | 7582830 | + | XP_035441745.1 | 220 | P450 4V2-like | / | / |
| 108 | 118270774 | Chr 14 | 7670477 | 7683905 | + | XP_035442441.1 | 496 | P450 4C1-like | GSSPFG00027168001.2-PA gene=CYP340AD3 partial | 99 |
| 109 | 118270778 | Chr 14 | 7688782 | 7699833 | + | XP_035442446.1 | 488 | P450 4C1-like | GSSPFG00027152001.1-PA gene=GSSPFG00027152001.1 | 98 |
| 110 | 118270719 | Chr 14 | 5671943 | 5676720 | - | XP_035442369.1 | 432 | P450 4C1-like isoform X1 | GSSPFG00030289001.2-PA gene=CYP421B1 | 83 |
|  |  | Chr 14 | 5671943 | 5676720 | - | XP_035442370.1 | 431 | P450 4C1-like isoform X2 | GSSPFG00030289001.2-PA gene=CYP421B1 | 83 |
| 111 | 118270718 | Chr 14 | 5677872 | 5687026 | - | XP_035442365.1 | 784 | P450 4C1-like isoform X1 | GSSPFG00005208001-PA gene=GSSPFG00005208001 | 98 |
|  |  | Chr 14 | 5677872 | 5684083 | - | XP_035442366.1 | 721 | P450 4C1-like isoform X2 | GSSPFG00005208001-PA gene=GSSPFG00005208001 | 98 |
|  |  | Chr 14 | 5677872 | 5681918 | - | XP_035442367.1 | 446 | P450 4C1-like isoform X3 | GSSPFG00030289001.2-PA gene=CYP421B1 | 68 |
| 112 | 118270594 | Chr 14 | 5688755 | 5693670 | - | XP_035442118.1 | 487 | P450 4C1-like | GSSPFG00005210001-PA gene=GSSPFG00005210001 | 97 |
|  |  | Chr 14 | 5688755 | 5693670 | - | XP_035442119.1 | 487 |  |  |  |
| 113 | 118270854 | Chr 14 | 6307835 | 6323930 | - | XP_035442531.1 | 947 | P450 4V2-like | GSSPFG00031882001-PA gene=GSSPFG00031882001 | 66 |
| 114 | 118270689 | Chr 14 | 6334194 | 6339289 | - | XP_035442321.1 | 490 | P450 4C1-like | GSSPFG00031882001-PA gene=GSSPFG00031882001 | 94 |
| 115 | 118270691 | Chr 14 | 6341549 | 6352366 | - | XP_035442322.1 | 490 | P450 4C1-like | GSSPFG00031882001-PA gene=GSSPFG00031882001 | 98 |
| 116 | 118270461 | Chr 14 | 6427610 | 6432966 | - | XP_035441947.1 | 360 | P450 4C1-like | GSSPFG00017290001.2-PB gene=CYP340L16CTERM | 97 |
| 117 | 118270728 | Chr 14 | 6487714 | 6500351 | - | XP_035442380.1 | 492 | P450 4c3-like | GSSPFG00027679001-PA gene=GSSPFG00027679001 | 78 |
| 118 | 118270576 | Chr 14 | 6524347 | 6532176 | - | XP_035442093.1 | 490 | P450 4C1-like | GSSPFG00014033001.2-PA gene=CYP340L1 fragment3 | 99 |
| 119 | 118270574 | Chr 14 | 6534840 | 6542670 | - | XP_035442091.1 | 490 | P450 4C1-like | GSSPFG00014033001.2-PA gene=CYP340L1 fragment3 | 97 |
| 120 | 118270577 | Chr 14 | 6545331 | 6553772 | - | XP_035442094.1 | 431 | P450 4C1-like | GSSPFG00014033001.2-PA gene=CYP340L1 fragment3 | 96 |
| 121 | 118270578 | Chr 14 | 6565073 | 6570700 | - | XP_035442095.1 | 490 | P450 4C1-like | GSSPFG00031119001.2-PA gene=CYP340AB1 | 49 |
| 122 | 118270575 | Chr 14 | 6573537 | 6584606 | - | XP_035442092.1 | 490 | P450 4V2-like | GSSPFG00031119001.2-PA gene=CYP340AB1 | 96 |
| 123 | 118270758 | Chr 14 | 6592130 | 6609764 | - | XP_035442419.1 | 485 | P450 4C1-like | GSSPFG00031119001.2-PA gene=CYP340AB1 | 46 |
| 124 | 118270860 | Chr 14 | 7333188 | 7343364 | - | XP_035442538.1 | 511 | P450 4C1-like | GSSPFG00031882001-PA gene=GSSPFG00031882001 | 64 |
| 125 | 118270313 | Chr 14 | 7389974 | 7396265 | - | XP_035441741.1 | 487 | P450 4C1-like | GSSPFG00031119001.2-PA gene=CYP340AB1 | 44 |
| 126 | 118270308 | Chr 14 | 7405180 | 7419560 | - | XP_035441735.1 | 492 | P450 4c21-like | GSSPFG00027679001-PA gene=GSSPFG00027679001 | 97 |
| 127 | 118270307 | Chr 14 | 7549064 | 7560628 | - | XP_035441733.1 | 492 | P450 4C1-like | GSSPFG00027679001-PA gene=GSSPFG00027679001 | 78 |
| 128 | 118270309 | Chr 14 | 7583679 | 7590379 | - | XP_035441736.1 | 490 | P450 4C1-like | GSSPFG00014033001.2-PA gene=CYP340L1 fragment3 | 99 |
| 129 | 118270310 | Chr 14 | 7592864 | 7597381 | - | XP_035441737.1 | 490 | P450 4C1-like | GSSPFG00031119001.2-PA gene=CYP340AB1 | 48 |
| 130 | 118270316 | Chr 14 | 7607490 | 7615573 | - | XP_035441746.1 | 490 | P450 4V2-like | GSSPFG00031119001.2-PA gene=CYP340AB1 | 97 |
| 131 | 118270312 | Chr 14 | 7626686 | 7641537 | - | XP_035441740.1 | 485 | P450 4C1-like | GSSPFG00031119001.2-PA gene=CYP340AB1 | 46 |
| 132 | 118271197 | Chr 15 | 2292627 | 2294658 | - | XP_035443083.1 | 524 | P450 6B5-like | GSSPFG00003900001.2-PA gene=CYP6AE44 | 78 |
| 133 | 118271250 | Chr 15 | 8540431 | 8556753 | - | XP_035443151.1 | 529 | probable P450 301a1, mitochondrial | GSSPFG00028615001.3-PA gene=CYP301A1 fragment3 | 98 |
| 134 | 118272240 | Chr 16 | 8675383 | 8682993 | + | XP_035444503.1 | 485 | P450 4c3-like | GSSPFG00028938001.2-PA gene=CYP4AU15V1 | 99 |
| 135 | 118271683 | Chr 16 | 8655841 | 8664728 | - | XP_035443718.1 | 494 | P450 4V2-like | GSSPFG00029216001.3-PA gene=CYP4AU17 | 96 |
| 136 | 118272349 | Chr 17 | 9984546 | 9986772 | + | XP_035444666.1 | 511 | P450 6B2-like | GSSPFG00017614001.2-PC gene=CYP6AB61 | 99 |
| 137 | 118274242 | Chr 19 | 527240 | 547398 | + | XP_035447568.1 | 500 | P450 6k1-like | GSSPFG00030424001.1-PA gene=GSSPFG00030424001.1 | 98 |
| 138 | 118274285 | Chr 19 | 553614 | 561660 | + | XP_035447641.1 | 505 | P450 6k1-like | GSSPFG00017166001.2-PA gene=CYP324A16 | 99 |
| 139 | 118274407 | Chr 19 | 1681300 | 1697435 | + | XP_035447792.1 | 564 | probable P450 6a17 | GSSPFG00032991001.2-PA gene=CYP365A1 partial | 99 |
| 140 | 118274106 | Chr 19 | 2082004 | 2094757 | + | XP_035447373.1 | 564 | P450 6B1-like | GSSPFG00032991001.2-PA gene=CYP365A1 partial | 99 |
| 141 | 118273913 | Chr 19 | 6008996 | 6010894 | + | XP_035447007.1 | 504 | P450 6B7-like | GSSPFG00034089001.3-PA gene=GSSPFG00034089001.3 | 98 |
| 142 | 118273911 | Chr 19 | 6015394 | 6017413 | + | XP_035447005.1 | 505 | P450 6B6-like | GSSPFG00003241001.2-PB gene=CYP6B41V2 partial | 97 |
| 143 | 118273912 | Chr 19 | 6018546 | 6020374 | + | XP_035447006.1 | 504 | P450 6B6-like | GSSPFG00034089001.3-PA gene=GSSPFG00034089001.3 | 75 |
| 144 | 118273914 | Chr 19 | 6024101 | 6026373 | + | XP_035447008.1 | 503 | P450 6B2-like | GSSPFG00034089001.3-PA gene=GSSPFG00034089001.3 | 75 |
| 145 | 118273915 | Chr 19 | 6028183 | 6030458 | + | XP_035447009.1 | 503 | P450 6B2-like | GSSPFG00034089001.3-PA gene=GSSPFG00034089001.3 | 76 |
| 146 | 118274094 | Chr 19 | 10086603 | 10088292 | + | XP_035447350.1 | 518 | P450 6B6-like | GSSPFG00012279001.2-PB gene=CYP6AB59 | 98 |
| 147 | 118273800 | Chr 19 | 473093 | 476268 | - | XP_035446809.1 | 523 | P450 6B2-like isoform X1 | GSSPFG00026621001.1-PA gene=GSSPFG00026621001.1 | 99 |
|  |  | Chr 19 | 473093 | 481701 | - | XP_035446810.1 | 519 | P450 6B2-like isoform X2 | GSSPFG00017257001.4-PA gene=CYP6AE74 partial | 99 |
|  |  | Chr 19 | 473093 | 483435 | - | XP_035446811.1 | 519 | P450 6B2-like isoform X3 | GSSPFG00017256001.2-PA gene=CYP6AE70 partial | 97 |
|  |  | Chr 19 | 473093 | 485141 | - | XP_035446812.1 | 519 | P450 6B2-like isoform X4 | GSSPFG00017257001.4-PA gene=CYP6AE74 partial | 97 |
|  |  | Chr 19 | 473093 | 491281 | - | XP_035446813.1 | 519 | P450 6B2-like isoform X5 | GSSPFG00017255001.4-PA gene=CYP6AE69 partial | 97 |
| 148 | 118274311 | Chr 19 | 491422 | 493596 | - | XP_035447666.1 | 464 | P450 6B2-like | GSSPFG00035325001.3-PA gene=CY6AE72 partial | 94 |
| 149 | 118273960 | Chr 19 | 1402636 | 1437671 | - | XP_035447139.1 | 490 | P450 4C1-like | GSSPFG00001269001-PA gene=GSSPFG00001269001 | 96 |
|  |  | Chr 19 | 1402636 | 1437671 | - | XP_035447140.1 | 490 |  |  |  |
| 150 | 118273959 | Chr 19 | 1467158 | 1485465 | - | XP_035447138.1 | 495 | P450 4C1-like | GSSPFG00019109001-PA gene=GSSPFG00019109001 | 99 |
| 151 | 118274185 | Chr 19 | 2633775 | 2634664 | - | XP_035447508.1 | 134 | P450 6a9-like | / | / |
| 152 | 118274283 | Chr 19 | 4513182 | 4516063 | - | XP_035447638.1 | 501 | P450 6B6-like | GSSPFG00035425001.4-PA gene=GSSPFG00035425001.4 | 98 |
| 153 | 118274175 | Chr 19 | 10095305 | 10097364 | - | XP_035447497.1 | 515 | P450 6B6-like | GSSPFG00033556001.2-PA gene=CYP6AB60 | 99 |
| 154 | 118275081 | Chr 20 | 7060282 | 7084282 | + | XP_035448830.1 | 495 | P450 4C1-like | GSSPFG00009679001.2-PA gene=CYP367A12 | 67 |
|  |  | Chr 20 | 7060282 | 7084282 | + | XP_035448831.1 | 495 |  |  |  |
| 155 | 118275193 | Chr 20 | 7098527 | 7104690 | + | XP_035448956.1 | 495 | P450 4C1-like | GSSPFG00009679001.2-PA gene=CYP367A12 | 99 |
| 156 | 118274795 | Chr 20 | 7119723 | 7129325 | + | XP_035448397.1 | 500 | P450 4C1-like | GSSPFG00033942001-PA gene=GSSPFG00033942001 | 98 |
| 157 | 118274506 | Chr 20 | 12631495 | 12633367 | + | XP_035447901.1 | 525 | P450 6B5-like | GSSPFG00013634001.3-PA gene=CYP6AE49 | 78 |
| 158 | 118274503 | Chr 20 | 12814445 | 12816294 | + | XP_035447899.1 | 525 | P450 6B5-like | GSSPFG00013634001.3-PA gene=CYP6AE49 | 77 |
| 159 | 118274504 | Chr 20 | 12833541 | 12836404 | + | XP_035447900.1 | 525 | P450 6B4-like | GSSPFG00013634001.3-PA gene=CYP6AE49 | 98 |
| 160 | 118275709 | Chr 21 | 7312656 | 7316779 | + | XP_035449665.1 | 517 | probable P450 6a14 | GSSPFG00018442001.3-PA gene=CYP6CT1 | 99 |
| 161 | 118279835 | Chr 28 | 8144467 | 8146605 | + | XP_035455539.1 | 514 | P450 6B7-like | GSSPFG00027565001.2-PA gene=CYP6AB58 | 98 |
| 162 | 118279736 | Chr 28 | 10129878 | 10133463 | + | XP_035455352.1 | 514 | P450 6B7-like | GSSPFG00027565001.2-PA gene=CYP6AB58 | 98 |
| 163 | 118279836 | Chr 28 | 8157208 | 8158946 | - | XP_035455540.1 | 514 | P450 6B7-like | GSSPFG00027565001.2-PB gene=CYP6AB58 | 83 |
| 164 | 118279731 | Chr 28 | 10143271 | 10145021 | - | XP_035455344.1 | 514 | P450 6B7-like | GSSPFG00027565001.2-PB gene=CYP6AB58 | 83 |
| 165 | 118281189 | Chr 31 | 1196148 | 1202471 | + | XP_035457638.1 | 539 | P450 307a1-like | GSSPFG00002834001.2-PA gene=CYP307A2 partial | 100 |
| 166 | 118281224 | Chr 31 | 4601583 | 4609121 | - | XP_035457685.1 | 539 | P450 307a1-like | GSSPFG00002834001.2-PA gene=CYP307A2 partial | 100 |
| 167 | 118281843 | Un | 84452 | 91613 | + | XP_035458500.1 | 485 | P450 4c3-like | GSSPFG00028938001.2-PA gene=CYP4AU15V1 | 98 |
| 168 | 118281717 | Un | 93640 | 112825 | + | XP_035458293.1 | 495 | P450 4C1-like | GSSPFG00009679001.2-PA gene=CYP367A12 | 67 |
| 169 | 118281718 | Un | 120497 | 126706 | + | XP_035458294.1 | 494 | P450 4C1-like | GSSPFG00009679001.2-PA gene=CYP367A12 | 99 |
| 170 | 118281716 | Un | 139966 | 150026 | + | XP_035458292.1 | 500 | P450 4C1-like | GSSPFG00033942001-PA gene=GSSPFG00033942001 | 98 |
| 171 | 118281625 | Un | 57 | 1943 | - | XP_035458145.1 | 198 | P450 302a1, mitochondrial-like, partial | GSSPFG00016942001.2-PA gene=CYP302A1 | 97 |
| 172 | 118282155 | Un | 28552 | 31689 | - | XP_035458985.1 | 517 | P450 6B6-like | GSSPFG00019609001-PA gene=GSSPFG00019609001 | 99 |
|  |  | Un | 28552 | 31689 | - | XP_035458986.1 | 517 |  |  |  |
|  |  | Un | 28552 | 31689 | - | XP_035458987.1 | 517 |  |  |  |
| 173 | 118281824 | Un | 70864 | 79519 | - | XP_035458450.1 | 494 | P450 4V2-like | GSSPFG00029216001.3-PA gene=CYP4AU17 | 96 |

/, means we could not find the homologous P450s in the potein data of FAW (Gouin et al., 2017).

**Supplement table 11 Nine gene loci in the FAW genome which possible relates to P450 family**

| No. | GeneID | Chr NO. | Start | Stop | Strand | Protein Product | Length  (aa) | BlastP (Expect value≤1.0E-100)  (select the first alignments target) | | Amino acid seq.identities (%) |
| --- | --- | --- | --- | --- | --- | --- | --- | --- | --- | --- |
|  |  |  |  |  |  |  |  | **Protein Name**  **(Xiao et al., 2020)** | **Protein Name**  **(Gouin et al., 2017)** |  |
| 1 | 118270597 | Chr2 | 6491176 | 6500688 | - | transcribed  pseudogene | / | / | GSSPFG00018886001.5-RA gene=CYP4M15V2^#^ | 98^#^ |
| 2 | 118264847 | Chr6 | 17223945 | 17254102 | + | protein_coding | 2251 | uncharacterized LOC118264847 && Q964T2.1 RecName: Full=Cytochrome P450 9e2**^＆^** | Amino acid sequence regions:1-529: GSSPFG00024476001.2-PA gene=CYP9A58 | 75 |
|  |  |  |  |  |  |  |  |  | Amino acid sequence regions:640-1169: GSSPFG00024476001.2-PA gene=CYP9A58 | 99 |
|  |  |  |  |  |  |  |  |  | Amino acid sequence regions:1182-1712: GSSPFG00024476001.2-PA gene=CYP9A58 | 64 |
|  |  |  |  |  |  |  |  |  | AA sequence regions:1727-2250: GSSPFG00024476001.2-PA gene=CYP9A58 | 65 |
| 3 | 118264846 | Chr6 | 17198356 | 17218444 | + | protein_coding | 2206 | uncharacterized LOC118264846 && Q964T2.1 RecName: Full=Cytochrome P450 9e2**^＆^** | Amino acid sequence regions:1-528: GSSPFG00008268001.2-PA gene=CYP9A30 | 99 |
|  |  |  |  |  |  |  |  |  | Amino acid sequence regions:615-1144: GSSPFG00008268001.2-PA gene=CYP9A30 | 80 |
|  |  |  |  |  |  |  |  |  | Amino acid sequence regions:1678-2204: GSSPFG00008268001.2-PA gene=CYP9A30 | 61 |
| 4 | 118264849 | Chr6 | 17558935 | 17573574 | + | protein_coding | 1667 | uncharacterized LOC118264849 && Q9VG82.1 RecName: Full=Probable cytochrome P450 9f2**^＆^** | Amino acid sequence regions:47-574: GSSPFG00024476001.2-PA gene=CYP9A58 | 86 |
|  |  |  |  |  |  |  |  |  | Amino acid sequence regions:575-1103: GSSPFG00024476001.2-PA gene=CYP9A58 | 63 |
|  |  |  |  |  |  |  |  |  | Amino acid sequence regions:1138-1667:GSSPFG00024476001.2-PA gene=CYP9A58 | 98 |
| 5 | 118264068 | Chr6 | 17190502 | 17197078 | + | Transcribed  pseudogene | / | / | GSSPFG00008269001.4-RA gene=CYP9A31PARTIAL^#^ | 100^#^ |
| 6 | 118264850 | Chr6 | 17573890 | 17592827 | + | protein_coding | 1089 | uncharacterized LOC118264850 && Q964T2.1 RecName: Full=Cytochrome P450 9e2**^＆^** | Amino acid sequence regions:1-386: GSSPFG00033969001.2-PA gene=CYP9A59PARTIAL | 92 |
|  |  |  |  |  |  |  |  |  | Amino acid sequence regions:565-944: GSSPFG00033969001.2-PA gene=CYP9A59PARTIAL | 56 |
| 7 | 118271251 | Chr15 | 8515903 | 8522493 | - | lncRNA | / | / | GSSPFG00035682001.2-PA gene=CYP301B1 fragment2 | 93 |
| 8 | 118272796 | Chr17 | 7797450 | 7803289 | + | protein_coding | 492 | farnesoate epoxidase-like && C0SPF7.2 AltName: Full=Cytochrome P450 15C1 | GSSPFG00020555001.2-PA gene=CYP15C1 | 98 |
| 9 | 118278003 | Chr25 | 6402468 | 6424711 | - | protein_coding | 517 | ecdysone 20-monooxygenase-like%2C transcript variant X1 && Q9VUF8.3 AltName: Full=Cytochrome P450 314a1, mitochondrial | GSSPFG00014039001.4-PA gene=CYP314A1 | 99 |

＆, The annotation of this candidate gene may be wrong, because there is actually more than one P450 gene within this locus.

#, Although it was considered as a pseudogene, we could find its peptide products homologous to protein of known P450.

**Supplement Table 12 CPs genes which were descripted in the annotation files of FAW genome (Xiao et al. 2020)**

| No | Gene ID | Chr. | Start | Stop | Strand | Protein Product | Length(aa) | Gene description |
| --- | --- | --- | --- | --- | --- | --- | --- | --- |
| 1 | 118276210 | Chr 2 | 13275613 | 13278419 | + | XP_035450341.1 | 197 | larval cuticle protein LCP-30-like |
| 2 | 118276814 | Chr 2 | 13028572 | 13031343 | + | XP_035451272.1 | 197 | larval cuticle protein LCP-30-like |
| 3 | 118274522 | Chr 2 | 8705189 | 8711284 | - | XP_035447939.1 | 437 | protein naked cuticle homolog |
| 4 | 118282269 | Chr 3 | 9293952 | 9309135 | + | LOC118282269 ^&^ | 2040 | uncharacterized LOC118282269 |
| 5 | 118282374 | Chr 3 | 9614037 | 9615358 | + | XP_035459323.1 | 134 | endocuticle structural glycoprotein ABD-5-like |
| 6 | 118282341 | Chr 3 | 9765977 | 9769217 | + | LOC118282341 ^&^ | 1176 | uncharacterized LOC118282341 |
| 7 | 118281432 | Chr 3 | 11980539 | 11983892 | + | XP_035457901.1 | 195 | cuticle protein 19-like |
| 8 | 118281375 | Chr 3 | 12082224 | 12083054 | + | XP_035457844.1 | 220 | cuticle protein 8-like |
| 9 | 118281423 | Chr 3 | 12111590 | 12115307 | + | XP_035457891.1 | 177 | cuticle protein 7-like isoform X1 |
| 10 | 118281423 | Chr 3 | 12111590 | 12112293 | + | XP_035457896.1 | 176 | cuticle protein 7-like |
| 11 | 118281416 | Chr 3 | 12114573 | 12115307 | + | XP_035457883.1 | 187 | cuticle protein 7-like |
| 12 | 118261754 | Chr 3 | 12177489 | 12178132 | + | XP_035428596.1 | 127 | cuticle protein 19-like |
| 13 | 118261756 | Chr 3 | 12181581 | 12182209 | + | XP_035428598.1 | 127 | cuticle protein 19-like |
| 14 | 118261758 | Chr 3 | 12184587 | 12186642 | + | XP_035428602.1 | 147 | cuticle protein 19-like |
| 15 | 118261762 | Chr 3 | 12202428 | 12203101 | + | XP_035428605.1 | 129 | cuticle protein 18.6-like |
| 16 | 118261991 | Chr 3 | 12228255 | 12230485 | + | LOC118261991 ^&^ | 1232 | uncharacterized LOC118261991 |
| 17 | 118262009 | Chr 3 | 12622140 | 12624075 | + | XP_035428989.1 | 195 | cuticle protein 19-like |
| 18 | 118262019 | Chr 3 | 12730797 | 12731627 | + | XP_035429011.1 | 220 | cuticle protein 8-like |
| 19 | 118262026 | Chr 3 | 12757385 | 12758088 | + | XP_035429017.1 | 176 | cuticle protein 7-like |
| 20 | 118262024 | Chr 3 | 12760359 | 12761092 | + | XP_035429015.1 | 187 | cuticle protein 7-like |
| 21 | 118261796 | Chr 3 | 12818509 | 12824378 | + | XP_035428647.1 | 127 | cuticle protein 19-like |
| 22 | 118261799 | Chr 3 | 12827726 | 12828777 | + | XP_035428650.1 | 130 | cuticle protein 18.6-like |
| 23 | 118261802 | Chr 3 | 12843422 | 12844145 | + | XP_035428654.1 | 150 | cuticle protein 18.6-like |
| 24 | 118282313 | Chr 3 | 4761122 | 4769301 | - | XP_035459228.1 | 236 | cuticle protein 8-like isoform X2 |
| 25 | 118262125 | Chr 3 | 6629173 | 6637915 | - | XP_035429164.1 | 236 | cuticle protein 8-like isoform X2 |
| 26 | 118261958 | Chr 3 | 9596499 | 9601160 | - | XP_035428927.1 | 103 | endocuticle structural glycoprotein ABD-5-like |
| 27 | 118261870 | Chr 3 | 9617974 | 9620132 | - | XP_035428778.1 | 103 | endocuticle structural glycoprotein SgAbd-5-like |
| 28 | 118262087 | Chr 3 | 9621135 | 9634094 | - | XP_035429098.1 | 107 | endocuticle structural glycoprotein ABD-5-like |
| 29 | 118261848 | Chr 3 | 9655115 | 9655915 | - | XP_035428739.1 | 115 | endocuticle structural glycoprotein SgAbd-5-like |
| 30 | 118261769 | Chr 3 | 9656750 | 9657484 | - | XP_035428613.1 | 114 | endocuticle structural glycoprotein SgAbd-5-like |
| 31 | 118282330 | Chr 3 | 9659118 | 9659871 | - | XP_035459257.1 | 121 | endocuticle structural glycoprotein SgAbd-5-like |
| 32 | 118261898 | Chr 3 | 9660499 | 9661214 | - | XP_035428813.1 | 112 | endocuticle structural protein SgAbd-6-like |
| 33 | 118282407 | Chr 3 | 9662911 | 9663723 | - | XP_035459366.1 | 115 | endocuticle structural glycoprotein SgAbd-5-like |
| 34 | 118261868 | Chr 3 | 9669318 | 9670192 | - | XP_035428776.1 | 117 | endocuticle structural glycoprotein SgAbd-5-like |
| 35 | 118282288 | Chr 3 | 9672859 | 9673779 | - | XP_035459178.1 | 120 | endocuticle structural glycoprotein SgAbd-5-like |
| 36 | 118282353 | Chr 3 | 9687567 | 9692827 | - | XP_035459286.1 | 147 | larval cuticle protein 1-like |
| 37 | 118282354 | Chr 3 | 9698322 | 9700477 | - | XP_035459289.1 | 119 | endocuticle structural glycoprotein SgAbd-5-like |
| 38 | 118282356 | Chr 3 | 9702382 | 9703640 | - | XP_035459292.1 | 119 | endocuticle structural glycoprotein SgAbd-5-like |
| 39 | 118282355 | Chr 3 | 9705703 | 9706960 | - | XP_035459291.1 | 127 | endocuticle structural glycoprotein SgAbd-5-like |
| 40 | 118261896 | Chr 3 | 9712400 | 9717355 | - | XP_035428812.1 | 151 | endocuticle structural glycoprotein SgAbd-2-like |
| 41 | 118261895 | Chr 3 | 9731589 | 9740177 | - | XP_035428811.1 | 180 | endocuticle structural glycoprotein ABD-4-like |
| 42 | 118282343 | Chr 3 | 9747124 | 9751279 | - | XP_035459274.1 | 214 | endocuticle structural glycoprotein SgAbd-8-like |
| 43 | 118282340 | Chr 3 | 9759062 | 9764415 | - | LOC118282340 ^&^ | 4659 | uncharacterized LOC118282340 |
| 44 | 118282348 | Chr 3 | 9774487 | 9793929 | - | XP_035459282.1 | 133 | endocuticle structural glycoprotein ABD-4-like |
| 45 | 118282345 | Chr 3 | 9808958 | 9809813 | - | XP_035459276.1 | 184 | larval cuticle protein LCP-22-like |
| 46 | 118282346 | Chr 3 | 9814127 | 9815126 | - | XP_035459277.1 | 149 | larval cuticle protein LCP-22-like |
| 47 | 118282347 | Chr 3 | 9823633 | 9824635 | - | XP_035459281.1 | 149 | larval cuticle protein LCP-22-like isoform X3 |
| 48 | 118282342 | Chr 3 | 9845466 | 9848240 | - | XP_035459273.1 | 321 | larval cuticle protein LCP-30-like |
| 49 | 118282344 | Chr 3 | 9856162 | 9857584 | - | XP_035459275.1 | 188 | larval cuticle protein LCP-17-like |
| 50 | 118282349 | Chr 3 | 9866623 | 9867193 | - | XP_035459283.1 | 127 | endocuticle structural glycoprotein SgAbd-8-like |
| 51 | 118282304 | Chr 3 | 9873068 | 9873692 | - | XP_035459217.1 | 132 | endocuticle structural glycoprotein SgAbd-8-like |
| 52 | 118282151 | Chr 3 | 9893661 | 9894334 | - | XP_035458982.1 | 147 | larval cuticle protein 16/17-like |
| 53 | 118282159 | Chr 3 | 9900814 | 9902190 | - | XP_035458995.1 | 131 | larval cuticle protein 1-like |
| 54 | 118282178 | Chr 3 | 9907768 | 9908291 | - | XP_035459025.1 | 111 | larval cuticle protein 1-like isoform X2 |
| 55 | 118282167 | Chr 3 | 9912527 | 9913311 | - | XP_035459007.1 | 113 | larval cuticle protein 1-like |
| 56 | 118261853 | Chr 3 | 9917119 | 9918029 | - | XP_035428746.1 | 134 | larval cuticle protein 1-like |
| 57 | 118281401 | Chr 3 | 12008347 | 12009288 | - | XP_035457869.1 | 199 | cuticle protein 8-like |
| 58 | 118261732 | Chr 3 | 12015343 | 12020745 | - | XP_035428574.1 | 359 | cuticle protein 18.6-like |
| 59 | 118261733 | Chr 3 | 12025494 | 12029015 | - | XP_035428575.1 | 290 | cuticle protein 18.6-like |
| 60 | 118261734 | Chr 3 | 12031590 | 12035434 | - | XP_035428576.1 | 330 | cuticle protein 18.6-like |
| 61 | 118281445 | Chr 3 | 12037946 | 12038806 | - | XP_035457914.1 | 162 | cuticle protein 7-like |
| 62 | 118281347 | Chr 3 | 12046162 | 12047886 | - | LOC118281347 ^&^ | 1481 | uncharacterized LOC118281347 |
| 63 | 118281454 | Chr 3 | 12051932 | 12056111 | - | XP_035457922.1 | 127 | cuticle protein 19-like |
| 64 | 118281367 | Chr 3 | 12059244 | 12062256 | - | XP_035457839.1 | 244 | cuticle protein 8-like |
| 65 | 118281439 | Chr 3 | 12069142 | 12070191 | - | XP_035457908.1 | 163 | cuticle protein 8-like |
| 66 | 118281391 | Chr 3 | 12080363 | 12081079 | - | XP_035457861.1 | 208 | cuticle protein 8-like |
| 67 | 118261737 | Chr 3 | 12085407 | 12089782 | - | XP_035428578.1 | 449 | cuticle protein-like |
| 68 | 118281381 | Chr 3 | 12101051 | 12101860 | - | XP_035457852.1 | 213 | cuticle protein 7-like |
| 69 | 118281406 | Chr 3 | 12116960 | 12117696 | - | XP_035457877.1 | 187 | cuticle protein 7-like |
| 70 | 118261743 | Chr 3 | 12131330 | 12131878 | - | XP_035428584.1 | 107 | cuticle protein 18.6-like |
| 71 | 118261748 | Chr 3 | 12155683 | 12156503 | - | XP_035428589.1 | 130 | cuticle protein 18.6-like |
| 72 | 118281467 | Chr 3 | 12158571 | 12159504 | - | XP_035457935.1 | 123 | cuticle protein 19-like |
| 73 | 118261749 | Chr 3 | 12162376 | 12163444 | - | XP_035428590.1 | 146 | cuticle protein 19-like |
| 74 | 118261753 | Chr 3 | 12175749 | 12177451 | - | XP_035428595.1 | 136 | larval cuticle protein A2B-like |
| 75 | 118261755 | Chr 3 | 12179979 | 12180808 | - | XP_035428597.1 | 128 | cuticle protein 19-like |
| 76 | 118261757 | Chr 3 | 12182368 | 12184502 | - | XP_035428601.1 | 150 | cuticle protein 19-like |
| 77 | 118261759 | Chr 3 | 12187210 | 12190449 | - | XP_035428603.1 | 156 | cuticle protein-like |
| 78 | 118261760 | Chr 3 | 12191286 | 12191952 | - | XP_035428604.1 | 129 | cuticle protein 18.6-like |
| 79 | 118261764 | Chr 3 | 12211015 | 12211694 | - | XP_035428608.1 | 150 | cuticle protein 18.6-like |
| 80 | 118261992 | Chr 3 | 12233042 | 12234856 | - | XP_035428967.1 | 241 | cuticle protein 19-like |
| 81 | 118261962 | Chr 3 | 12237069 | 12239173 | - | XP_035428931.1 | 176 | cuticle protein 19-like |
| 82 | 118261963 | Chr 3 | 12260877 | 12265216 | - | XP_035428932.1 | 143 | cuticle protein 19-like |
| 83 | 118262071 | Chr 3 | 12387702 | 12392098 | - | XP_035429073.1 | 175 | cuticle protein 7-like |
| 84 | 118262072 | Chr 3 | 12397915 | 12403482 | - | XP_035429074.1 | 139 | cuticle protein 19-like |
| 85 | 118282308 | Chr 3 | 12416211 | 12418863 | - | XP_035459220.1 | 155 | cuticle protein 6-like |
| 86 | 118282350 | Chr 3 | 12422307 | 12428651 | - | XP_035459284.1 | 158 | cuticle protein 8-like |
| 87 | 118261907 | Chr 3 | 12460475 | 12463166 | - | XP_035428833.1 | 205 | cuticle protein 8-like |
| 88 | 118261773 | Chr 3 | 12637147 | 12639500 | - | XP_035428617.1 | 163 | larval cuticle protein A3A-like |
| 89 | 118262022 | Chr 3 | 12648381 | 12649296 | - | XP_035429013.1 | 193 | cuticle protein 8-like |
| 90 | 118262025 | Chr 3 | 12655942 | 12657294 | - | XP_035429016.1 | 184 | cuticle protein 19-like |
| 91 | 118262023 | Chr 3 | 12662241 | 12663452 | - | XP_035429014.1 | 192 | cuticle protein 7-like |
| 92 | 118262030 | Chr 3 | 12666840 | 12667622 | - | XP_035429020.1 | 162 | cuticle protein 7-like |
| 93 | 118261774 | Chr 3 | 12669905 | 12671298 | - | XP_035428618.1 | 143 | cuticle protein 7-like |
| 94 | 118261775 | Chr 3 | 12672644 | 12678766 | - | XP_035428619.1 | 329 | cuticle protein-like |
| 95 | 118262028 | Chr 3 | 12681261 | 12682111 | - | XP_035429019.1 | 162 | cuticle protein 7-like |
| 96 | 118262016 | Chr 3 | 12688337 | 12690067 | - | LOC118262016 ^&^ | 1491 | uncharacterized LOC118262016 |
| 97 | 118262027 | Chr 3 | 12716106 | 12717171 | - | XP_035429018.1 | 163 | cuticle protein 8-like |
| 98 | 118262018 | Chr 3 | 12725167 | 12727447 | - | LOC118262018 ^&^ | 1037 | uncharacterized LOC118262018 |
| 99 | 118262020 | Chr 3 | 12728936 | 12729652 | - | XP_035429012.1 | 208 | cuticle protein 8-like |
| 100 | 118261779 | Chr 3 | 12740199 | 12743820 | - | XP_035428625.1 | 398 | cuticle protein-like |
| 101 | 118261780 | Chr 3 | 12746305 | 12749819 | - | XP_035428626.1 | 471 | cuticle protein-like |
| 102 | 118262033 | Chr 3 | 12765410 | 12766145 | - | XP_035429024.1 | 187 | cuticle protein 7-like |
| 103 | 118261786 | Chr 3 | 12771311 | 12772163 | - | XP_035428636.1 | 102 | cuticle protein 19-like |
| 104 | 118261788 | Chr 3 | 12774602 | 12782285 | - | LOC118261788 | 777 | Cuticle protein 18.6 |
| 105 | 118261789 | Chr 3 | 12783009 | 12786591 | - | XP_035428640.1 | 282 | pupal cuticle protein Edg-84A-like |
| 106 | 118261790 | Chr 3 | 12787107 | 12788908 | - | XP_035428641.1 | 129 | cuticle protein 19-like |
| 107 | 118261798 | Chr 3 | 12825944 | 12826612 | - | XP_035428649.1 | 129 | cuticle protein 19-like |
| 108 | 118261804 | Chr 3 | 12869585 | 12871650 | - | XP_035428656.1 | 241 | cuticle protein 19-like |
| 109 | 118282419 | Chr 3 | 12872439 | 12874322 | - | XP_035459385.1 | 176 | cuticle protein 19-like |
| 110 | 118262049 | Chr 3 | 12895839 | 12901707 | - | XP_035429043.1 | 143 | cuticle protein 19-like |
| 111 | 118261840 | Chr 3 | 13064509 | 13067150 | - | XP_035428726.1 | 155 | cuticle protein 6-like |
| 112 | 118282332 | Chr 3 | 13070642 | 13078014 | - | XP_035459259.1 | 164 | cuticle protein 8-like |
| 113 | 118261945 | Chr 3 | 13109170 | 13111004 | - | XP_035428896.1 | 172 | cuticle protein 8-like |
| 114 | 118280910 | Chr 3 | 14669671 | 14674798 | - | LOC118280910 ^&^ | 1703 | uncharacterized LOC118280910 |
| 115 | 118262246 | Chr 4 | 15627295 | 15627612 | - | XP_035429332.1 | 105 | cuticle protein 63-like |
| 116 | 118263980 | Chr 6 | 12298856 | 12301200 | + | XP_035432168.1 | 224 | pupal cuticle protein 20-like |
| 117 | 118263981 | Chr 6 | 12133493 | 12135858 | + | XP_035432169.1 | 224 | pupal cuticle protein 20-like |
| 118 | 118263985 | Chr 6 | 12306124 | 12307471 | + | XP_035432174.1 | 165 | pupal cuticle protein 20-like |
| 119 | 118263986 | Chr 6 | 12309681 | 12311355 | - | XP_035432176.1 | 169 | pupal cuticle protein 20-like isoform X2 |
| 120 | 118263987 | Chr 6 | 12314446 | 12318045 | - | XP_035432177.1 | 149 | pupal cuticle protein 20-like |
| 121 | 118264710 | Chr 6 | 3658800 | 3660670 | - | XP_035433205.1 | 206 | cuticle protein 7-like |
| 122 | 118265064 | Chr 7 | 661959 | 674439 | - | LOC118265064 ^&^ | 1342 | uncharacterized LOC118265064 |
| 123 | 118265573 | Chr 7 | 246599 | 256674 | - | LOC118265573 ^&^ | 1254 | uncharacterized LOC118265573 |
| 124 | 118265978 | Chr 8 | 11762487 | 11763470 | + | XP_035435186.1 | 240 | cuticle protein 3-like |
| 125 | 118265980 | Chr 8 | 11768230 | 11769202 | + | XP_035435191.1 | 230 | cuticle protein 3-like |
| 126 | 118265973 | Chr 8 | 11786158 | 11790996 | + | LOC118265973 ^&^ | 4196 | uncharacterized LOC118265973 |
| 127 | 118266013 | Chr 8 | 11836295 | 11844766 | + | XP_035435242.1 | 336 | pupal cuticle protein 36-like |
| 128 | 118266051 | Chr 8 | 11860312 | 11861760 | + | XP_035435293.1 | 204 | pupal cuticle protein 36-like |
| 129 | 118266107 | Chr 8 | 11877161 | 11879500 | + | XP_035435373.1 | 196 | pupal cuticle protein 27-like |
| 130 | 118266528 | Chr 8 | 11972426 | 11981204 | + | XP_035435897.1 | 166 | endocuticle structural glycoprotein SgAbd-3-like |
| 131 | 118266475 | Chr 8 | 13397685 | 13403397 | + | LOC118266475 | 3156 | Pupal cuticle protein 36 |
| 132 | 118266269 | Chr 8 | 13413311 | 13425828 | + | LOC118266269 ^&^ | 3633 | uncharacterized LOC118266269 |
| 133 | 118266484 | Chr 8 | 13418183 | 13419172 | + | XP_035435851.1 | 240 | cuticle protein 3-like |
| 134 | 118266483 | Chr 8 | 13421794 | 13422784 | + | XP_035435849.1 | 240 | cuticle protein 2-like |
| 135 | novel.173 | Chr 8 | 13450413 | 13451460 | + | novel.173 ^&^ | 726 | uncharacterized protein |
| 136 | 118265846 | Chr 8 | 13499198 | 13509218 | + | XP_035434981.1 | 336 | pupal cuticle protein 36-like |
| 137 | 118265850 | Chr 8 | 13544826 | 13547477 | + | XP_035434986.1 | 196 | pupal cuticle protein 27-like |
| 138 | 118266524 | Chr 8 | 13648356 | 13656120 | + | XP_035435892.1 | 166 | endocuticle structural glycoprotein SgAbd-3-like |
| 139 | 118266417 | Chr 8 | 15801268 | 15802567 | + | XP_035435763.1 | 358 | cuticle protein-like |
| 140 | 118266419 | Chr 8 | 15804697 | 15805541 | + | XP_035435766.1 | 215 | cuticle protein 19-like |
| 141 | 118266418 | Chr 8 | 15808000 | 15808924 | + | XP_035435765.1 | 229 | cuticle protein 19-like |
| 142 | 118265979 | Chr 8 | 11772101 | 11773729 | - | XP_035435189.1 | 230 | cuticle protein 3-like isoform X2 |
| 143 | 118265977 | Chr 8 | 11780236 | 11781642 | - | XP_035435185.1 | 316 | pupal cuticle protein 36a-like |
| 144 | 118266482 | Chr 8 | 11870023 | 11871186 | - | XP_035435848.1 | 149 | pupal cuticle protein 36-like |
| 145 | 118266512 | Chr 8 | 11883034 | 11891724 | - | XP_035435880.1 | 172 | cuticle protein 3-like |
| 146 | 118265845 | Chr 8 | 13428834 | 13429786 | - | XP_035434979.1 | 231 | cuticle protein 3-like |
| 147 | 118265844 | Chr 8 | 13438016 | 13439001 | - | XP_035434978.1 | 240 | cuticle protein 3-like |
| 148 | 118265842 | Chr 8 | 13443063 | 13444185 | - | XP_035434977.1 | 316 | pupal cuticle protein 36a-like isoform X2 |
| 149 | 118265848 | Chr 8 | 13530055 | 13531149 | - | XP_035434984.1 | 213 | pupal cuticle protein 36-like isoform X2 |
| 150 | 118265849 | Chr 8 | 13534970 | 13540245 | - | XP_035434985.1 | 152 | pupal cuticle protein 36-like |
| 151 | 118265851 | Chr 8 | 13550568 | 13559171 | - | XP_035434987.1 | 172 | cuticle protein 3-like |
| 152 | 118266383 | Chr 8 | 13572765 | 13578315 | - | LOC118266383 ^&^ | 3362 | uncharacterized LOC118266383 |
| 153 | 118266485 | Chr 8 | 13586569 | 13590402 | - | XP_035435852.1 | 176 | cuticle protein 3-like |
| 154 | 118266390 | Chr 8 | 16444691 | 16445984 | - | XP_035435722.1 | 292 | cuticle protein 16.5-like |
| 155 | 118267802 | Chr 10 | 13205988 | 13208522 | + | XP_035437888.1 | 150 | larval cuticle protein F1-like |
| 156 | 118267715 | Chr 10 | 3228455 | 3229623 | - | LOC118267715 ^&^ | 1169 | uncharacterized LOC118267715 |
| 157 | 118268279 | Chr 11 | 9129765 | 9131272 | - | XP_035438589.1 | 134 | flexible cuticle protein 12-like |
| 158 | 118268277 | Chr 11 | 9142211 | 9142799 | - | XP_035438587.1 | 106 | flexible cuticle protein 12-like |
| 159 | 118268278 | Chr 11 | 9146599 | 9147194 | - | XP_035438588.1 | 106 | flexible cuticle protein 12-like |
| 160 | 118269434 | Chr 12 | 887840 | 890823 | + | XP_035440438.1 | 128 | larval cuticle protein LCP-14-like |
| 161 | 118269458 | Chr 12 | 903065 | 906404 | + | XP_035440470.1 | 140 | larval cuticle protein LCP-17-like |
| 162 | 118269378 | Chr 12 | 919054 | 920893 | + | XP_035440338.1 | 176 | cuticle protein CP14.6-like |
| 163 | 118269179 | Chr 12 | 12060940 | 12064063 | + | XP_035440042.1 | 128 | larval cuticle protein LCP-14-like |
| 164 | 118269443 | Chr 12 | 12075427 | 12079104 | + | XP_035440451.1 | 140 | larval cuticle protein LCP-17-like |
| 165 | 118269457 | Chr 12 | 12090879 | 12092600 | + | XP_035440469.1 | 138 | cuticle protein CP14.6-like |
| 166 | 118269802 | Chr 13 | 12142317 | 12143659 | + | XP_035441009.1 | 87 | larval/pupal cuticle protein H1C-like |
| 167 | 118269800 | Chr 13 | 12124147 | 12124689 | + | XP_035441008.1 | 153 | pupal cuticle protein C1B-like |
| 168 | 118272785 | Chr 17 | 7949488 | 7949809 | + | XP_035445355.1 | 78 | pupal cuticle protein C1B-like |
| 169 | 118272659 | Chr 17 | 7953137 | 7953512 | + | XP_035445181.1 | 77 | pupal cuticle protein C1B-like |
| 170 | 118272743 | Chr 17 | 7983036 | 7983681 | + | XP_035445288.1 | 94 | cuticle protein 63-like |
| 171 | 118272879 | Chr 17 | 7995235 | 7995737 | + | XP_035445488.1 | 138 | cuticle protein 16.5-like |
| 172 | 118272646 | Chr 17 | 8005344 | 8006301 | + | XP_035445159.1 | 163 | cuticle protein 16.5-like |
| 173 | 118272670 | Chr 17 | 8014724 | 8018504 | + | XP_035445191.1 | 97 | cuticle protein 70, isoforms A and B-like |
| 174 | 118272927 | Chr 17 | 8022012 | 8022722 | + | XP_035445547.1 | 236 | cuticle protein 16.5-like |
| 175 | 118272369 | Chr 17 | 8035756 | 8036352 | + | XP_035444708.1 | 198 | cuticle protein LPCP-23-like |
| 176 | 118272371 | Chr 17 | 8044097 | 8044567 | + | XP_035444710.1 | 156 | cuticle protein 21-like |
| 177 | 118272366 | Chr 17 | 8074855 | 8075655 | + | XP_035444705.1 | 266 | cuticle protein 16.5-like |
| 178 | 118272903 | Chr 17 | 8079095 | 8079895 | + | XP_035445518.1 | 266 | cuticle protein 16.5-like |
| 179 | 118272876 | Chr 17 | 7937794 | 7945482 | - | XP_035445484.1 | 307 | pupal cuticle protein C1B-like |
| 180 | 118272786 | Chr 17 | 7940797 | 7941398 | - | XP_035445356.1 | 77 | pupal cuticle protein C1B-like |
| 181 | 118272373 | Chr 17 | 8048690 | 8049157 | - | XP_035444711.1 | 155 | cuticle protein 21-like |
| 182 | 118272370 | Chr 17 | 8050430 | 8051026 | - | XP_035444709.1 | 198 | cuticle protein 16.5-like |
| 183 | 118272834 | Chr 17 | 8081866 | 8082882 | - | XP_035445426.1 | 338 | cuticle protein LPCP-23-like |
| 184 | 118272499 | Chr 17 | 8086779 | 8087333 | - | XP_035444944.1 | 184 | larval/pupal cuticle protein H1C-like |
| 185 | 118273653 | Chr 18 | 13056040 | 13056660 | + | XP_035446628.1 | 164 | cuticle protein 7-like |
| 186 | 118273654 | Chr 18 | 13057311 | 13057952 | + | XP_035446629.1 | 151 | larval/pupal rigid cuticle protein 66-like |
| 187 | 118273466 | Chr 18 | 13062455 | 13063421 | + | XP_035446338.1 | 198 | larval/pupal rigid cuticle protein 66-like |
| 188 | 118273319 | Chr 18 | 13072433 | 13073128 | + | XP_035446113.1 | 170 | larval/pupal rigid cuticle protein 66-like |
| 189 | 118273336 | Chr 18 | 13079184 | 13079879 | + | XP_035446133.1 | 170 | larval/pupal rigid cuticle protein 66-like |
| 190 | 118273295 | Chr 18 | 4452035 | 4453038 | - | XP_035446085.1 | 189 | larval cuticle protein A2B-like |
| 191 | 118273296 | Chr 18 | 4641324 | 4642321 | - | XP_035446086.1 | 189 | larval cuticle protein A2B-like |
| 192 | 118273370 | Chr 18 | 12994832 | 12995520 | - | XP_035446198.1 | 133 | larval/pupal rigid cuticle protein 66-like |
| 193 | 118273465 | Chr 18 | 13059340 | 13060274 | - | XP_035446337.1 | 183 | larval/pupal rigid cuticle protein 66-like isoform X2 |
| 194 | 118273639 | Chr 18 | 13082153 | 13082848 | - | XP_035446599.1 | 170 | larval/pupal rigid cuticle protein 66-like |
| 195 | 118273864 | Chr 19 | 5554597 | 5555151 | + | XP_035446927.1 | 184 | larval/pupal cuticle protein H1C-like |
| 196 | 118273856 | Chr 19 | 5609445 | 5610218 | + | XP_035446919.1 | 257 | cuticle protein 65-like |
| 197 | 118274215 | Chr 19 | 8247282 | 8253983 | + | LOC118274215 ^&^ | 1002 | uncharacterized LOC118274215 |
| 198 | 118274403 | Chr 19 | 13733931 | 13740780 | + | XP_035447786.1 | 417 | cuticle protein-like |
| 199 | 118273854 | Chr 19 | 5563930 | 5564730 | - | XP_035446916.1 | 266 | cuticle protein 16.5-like |
| 200 | 118273855 | Chr 19 | 5568032 | 5568832 | - | XP_035446917.1 | 266 | cuticle protein 16.5-like |
| 201 | 118273868 | Chr 19 | 5619792 | 5625073 | - | XP_035446932.1 | 97 | cuticle protein 70, isoforms A and B-like |
| 202 | 118273867 | Chr 19 | 5639421 | 5640258 | - | XP_035446931.1 | 119 | cuticle protein 16.5-like |
| 203 | 118273866 | Chr 19 | 5649491 | 5649999 | - | XP_035446930.1 | 138 | cuticle protein 16.5-like |
| 204 | 118273869 | Chr 19 | 5661153 | 5661743 | - | XP_035446933.1 | 94 | cuticle protein 63-like |
| 205 | 118274137 | Chr 19 | 13705875 | 13709278 | - | XP_035447420.1 | 327 | cuticle protein 18.6-like |
| 206 | 118274138 | Chr 19 | 13712585 | 13719101 | - | XP_035447421.1 | 309 | cuticle protein 18.6-like |
| 207 | 118274134 | Chr 19 | 13721675 | 13724782 | - | XP_035447415.1 | 225 | cuticle protein 8-like |
| 208 | 118274134 | Chr 19 | 13721675 | 13722349 | - | XP_035447416.1 | 185 | cuticle protein 8-like isoform X2 |
| 209 | 118274135 | Chr 19 | 13726870 | 13727605 | - | XP_035447417.1 | 185 | cuticle protein 8-like |
| 210 | 118274136 | Chr 19 | 13729779 | 13733070 | - | XP_035447418.1 | 345 | cuticle protein-like |
| 211 | 118274730 | Chr 20 | 1256555 | 1263862 | + | XP_035448307.1 | 247 | pupal cuticle protein-like |
| 212 | 118275060 | Chr 20 | 1542395 | 1550438 | + | XP_035448803.1 | 220 | pupal cuticle protein-like |
| 213 | 118275098 | Chr 20 | 11407105 | 11413689 | + | XP_035448852.1 | 183 | cuticle protein 16.8-like |
| 214 | 118274704 | Chr 20 | 10968330 | 10972663 | - | XP_035448266.1 | 183 | cuticle protein 16.8-like |
| 215 | 118275749 | Chr 21 | 5786172 | 5788114 | + | LOC118275749 ^&^ | 572 | adult-specific cuticular protein ACP-20-like |
| 216 | 118275283 | Chr 21 | 7865498 | 7866200 | - | XP_035449085.1 | 106 | endocuticle structural glycoprotein ABD-5-like |
| 217 | 118276091 | Chr 22 | 8668477 | 8669242 | + | XP_035450147.1 | 226 | larval/pupal cuticle protein H1C-like |
| 218 | 118276092 | Chr 22 | 8677041 | 8677953 | + | XP_035450148.1 | 211 | cuticle protein 16.5-like |
| 219 | 118276403 | Chr 22 | 8700779 | 8701723 | - | XP_035450594.1 | 198 | cuticle protein 16.5-like |
| 220 | 118276281 | Chr 22 | 8703979 | 8705166 | - | XP_035450427.1 | 288 | cuticle protein 16.5-like |
| 221 | 118276464 | Chr 22 | 8718240 | 8719259 | - | XP_035450684.1 | 260 | cuticle protein 16.5-like |
| 222 | 118276778 | Chr 23 | 10524138 | 10529686 | + | LOC118276778 ^&^ | 1918 | pro-resilin-like |
| 223 | 118276799 | Chr 23 | 11136664 | 11167123 | + | LOC118276799 ^&^ | 1734 | uncharacterized LOC118276799 |
| 224 | 118276590 | Chr 23 | 12126680 | 12128339 | + | LOC118276590 ^&^ | 477 | pro-resilin-like |
| 225 | 118276965 | Chr 23 | 6380200 | 6409339 | - | LOC118276965 ^&^ | 2275 | uncharacterized LOC118276965 |
| 226 | 118276575 | Chr 23 | 7077632 | 7084009 | - | LOC118276575 ^&^ | 1455 | pro-resilin-like |
| 227 | 118276937 | Chr 23 | 11785272 | 11802202 | - | LOC118276937 ^&^ | 1254 | pro-resilin-like |
| 228 | 118276983 | Chr 23 | 12581331 | 12598891 | - | LOC118276983 ^&^ | 964 | pro-resilin-like |
| 229 | 118276729 | Chr 23 | 12635031 | 12640482 | - | LOC118276729 ^&^ | 1797 | pro-resilin-like |
| 230 | 118277988 | Chr 25 | 6510837 | 6521901 | + | XP_035452932.1 | 314 | larval cuticle protein A1A-like |
| 231 | 118277984 | Chr 25 | 6516432 | 6517147 | + | XP_035452922.1 | 168 | larval cuticle protein A2B-like |
| 232 | 118278046 | Chr 25 | 6525541 | 6531384 | + | XP_035452996.1 | 388 | cuticle protein-like |
| 233 | 118277982 | Chr 25 | 6532575 | 6533666 | + | XP_035452919.1 | 199 | larval cuticle protein A2B-like |
| 234 | 118277980 | Chr 25 | 6571951 | 6572669 | + | XP_035452917.1 | 203 | larval cuticle protein A3A-like |
| 235 | 118277907 | Chr 25 | 4937772 | 4938471 | - | XP_035452813.1 | 101 | cuticle protein 16.5-like |
| 236 | 118277983 | Chr 25 | 6523303 | 6524043 | - | XP_035452921.1 | 198 | larval cuticle protein A3A-like |
| 237 | 118277981 | Chr 25 | 6536392 | 6537095 | - | XP_035452918.1 | 203 | cuticle protein 7-like |
| 238 | 118277985 | Chr 25 | 6537769 | 6539738 | - | XP_035452923.1 | 143 | larval cuticle protein A2B-like |
| 239 | 118277978 | Chr 25 | 6543298 | 6544295 | - | XP_035452916.1 | 267 | cuticle protein 19.8-like |
| 240 | 118277977 | Chr 25 | 6548866 | 6549990 | - | XP_035452915.1 | 321 | cuticle protein 19.8-like |
| 241 | 118278717 | Chr 26 | 4543457 | 4568122 | + | XP_035453936.1 | 127 | endocuticle structural glycoprotein ABD-4-like |
| 242 | 118278803 | Chr 26 | 1357474 | 1392852 | - | XP_035454058.1 | 127 | endocuticle structural glycoprotein ABD-4-like |
| 243 | 118279159 | Chr 27 | 2036702 | 2052962 | - | XP_035454591.1 | 168 | larval cuticle protein A3A-like |
| 244 | 118279659 | Chr 28 | 3193283 | 3194188 | + | XP_035455243.1 | 240 | cuticle protein 16.5-like |
| 245 | 118279656 | Chr 28 | 3212999 | 3215522 | + | XP_035455240.1 | 284 | pupal cuticle protein PCP52-like |
| 246 | 118279662 | Chr 28 | 2736281 | 2743743 | - | XP_035455247.1 | 160 | cuticle protein 1-like |
| 247 | 118279657 | Chr 28 | 2758701 | 2761292 | - | XP_035455241.1 | 284 | pupal cuticle protein PCP52-like |
| 248 | 118279658 | Chr 28 | 2777962 | 2778867 | - | XP_035455242.1 | 240 | cuticle protein 16.5-like |
| 249 | 118280040 | Chr 28 | 6854669 | 6858998 | - | XP_035455805.1 | 89 | cuticle protein 1-like |
| 250 | 118280436 | Chr 29 | 8096080 | 8096964 | - | XP_035456361.1 | 167 | cuticle protein 16.5-like |
| 251 | 118280980 | Chr 31 | 7756421 | 7761401 | + | XP_035457293.1 | 136 | larval cuticle protein LCP-17-like |
| 252 | 118281698 | Un | 1064 | 13545 | + | LOC118281698 ^&^ | 1343 | uncharacterized LOC118281698 |

&, It contains a domain (PF00379: insect cuticle protein) and needs to be verified.

**Supplement Table 13. P value associated to fold change of seven target proteins**

| Gene_id  (gene description) | Larval body | | | | | | Larval midgut | |
| --- | --- | --- | --- | --- | --- | --- | --- | --- |
|  | **Cy/ck** | | **Av/ck** | | **Cl/ck** | | **CyM/ck** | |
|  | **log_2_**  **(FoldChange)** | **padj** | **log_2_**  **(FoldChange)** | **padj** | **log_2_**  **(FoldChange)** | **padj** | **log_2_**  **(FoldChange)** | **padj** |
| 118262401  [GABA(B) receptor subunit 1-like] | 1.304^﹟^ | 0.000 | 0.855 | 0.015 | 2.306^﹟^ | 0 | 1.005 | 0.176 |
| 118280690  [GABA(B) receptor subunit 2-like] | 1.319^﹟^ | 0.000 | 1.659^﹟^ | 0.000 | 2.587^﹟^ | 0 | 1.044 | 1 |
| 118277195  [GABA(A) receptor subunit beta-like] | 1.137^﹟^ | 0.005 | 1.598^﹟^ | 0.000 | 2.244^﹟^ | 0 | -0.149 | 1 |
| 118263634  [GABA(A) receptor subunit beta-like] | 1.118^﹟^ | 0.011 | 0.978 | 0.008 | 1.893^﹟^ | 0 | -1.701 | 1 |
| 118273029  [GABA(A) receptor subunit beta-like] | 1.000^﹟^ | 0.012 | 0.987 | 0.001 | 2.177^﹟^ | 0 | 1.071 | 1 |
| 118268071 (GluCl like) | 1.197^﹟^ | 0.025 | 1.623^﹟^ | 0.000 | 1.953^﹟^ | 0 | -2.425 | 1 |
| 118277865 (GluCl like) | 1.257^﹟^ | 0.000 | 0.591 | 0.077 | 0.681 | 0.137 | -1.253 | 1 |

Cy, cyproflanilide; Av, avermectin; Cl, chlorantraniliprole; ck, control group, CyM, midgut group under cyproflanilide stress.﹟, indicates this gene was considered as differential expression genes (DEGs) under the corresponding stress conditions. GABA, γ-aminobutyric acid, GluCl: glutamate-gated chloride channel-like

**Supplement Table 14 Comparison and analysis of two sets of differentially expressed P450 genes**

| DEGs specifically respond to cyproflanilide in this research  (18 P450,noted in Table 1) | |  |  | BlastP analysis (Expect value≤1.0E-100)  (select the first alignments target) | |
| --- | --- | --- | --- | --- | --- |
| Gene_id | **Protein_id** |  |  | **Gene_id (Identities values),according to Gui et al.(2022)** | **Regarded as DEGs under 23 pesticides stress**  **(P1~P23)** |
| 118264847^﹟^ | XP_035433400.1 |  |  | SFR13174 (96%) | P4、P8、P10、P18、P19、P20、P22、P23 |
| 118264846^﹟^ | XP_035433397.1 |  |  | SFR16592 (97%) | P18 |
| 118264849^﹟^ | XP_035433402.1 |  |  | SFR13174 (73%) | P4、P8、P10、P18、P19、P20、P22、P23 |
| 118273800 | XP_035446809.1 |  |  | SFR20451 (98%) | P4、P6、P8、P9、P10、P11、P16、P17、P18、P19、P20、P21、P22、P23 |
| 118273911^*^ | XP_035447005.1(up-regulated) |  |  | SFR10855 (98%) | Not regarded as DEGs |
| 118263357^*^ | XP_035431180.1(up-regulated) |  |  | SFR06040 (99%) | Not regarded as DEGs |
| 118266763 | XP_035436192.1 |  |  | SFR00474 (99%) | P10、P17 |
| 118270311 | XP_035441738.1 |  |  | / | / |
| 118269078 | XP_035439893.1 |  |  | SFR08808 (99%) | P4、P10、P14、P17、P18、P19、P20、P21、P22、P23 |
| 118268617 | XP_035439063.1 |  |  | / | / |
| 118263660 | XP_035431665.1 |  |  | SFR04210 (98%) | P20、P22 |
| 118264058^*^ | XP_035432293.1(up-regulated) |  |  | SFR13177 (97%) | Not regarded as DEGs |
| 118264056 | XP_035432291.1 |  |  | / | / |
| 118279731 | XP_035455344.1 |  |  | SFR06338 (98%) | P2、P4、P12、P13、P20、P23 |
| 118274106^*^ | XP_035447373.1(down-regulated) |  |  | SFR04638 (98%) | Not regarded as DEGs |
| 118272240^*^ | XP_035444503.1(up-regulated) |  |  | SFR02819 (98%) | Not regarded as DEGs |
| 118264055 | XP_035432290.1 |  |  | SFR16593 (98%) | P4、P20、P22 |
| 118264068 | misc_RNA^＆^ | |  | SFR16594 | P20、P22 |

﹟, This ID possible including several P450 members

* , P450 gene which changed significantly under stress of cyproflanilide, but not be DEGs under our avermectin and chlorantraniliprole treatments, and other 23 pesticdie treatments (Gui et al.,2022);

/, Did not find the same P450 gene in the annotations file of FAW genome (Gui et al.,2022);

＆, The prediction and annotation of this gene were different between Xiao et al.(2020) and Gui et al.(2022)

**Supplement Table 15 Real-time PCR primer**

| Gene_id | Gene_name | Primer name | Primer sequence |
| --- | --- | --- | --- |
| 118279155 | UGT2B19-like | UGT2B19-F | GCCTGGCATCCATTGCTCTA |
|  |  | UGT2B19-R | TGCCTTCCCAAATCGGATGA |
| 118276233 | Esterase FE4-like | FE4-F | TGCAGTCGTTGGAGACTTCC |
|  |  | FE4-R | TCCCTGAATCGGAGTTTGCC |
| 118269785 | GST1-like | GST1-F | CCTGGTTGATGGCGACTTCT |
|  |  | GST1-R | CCCCACCGAAGATTTGTGGA |
| 118273911 | CYP6B6-like | CYP6B6-F | CTTGCGAGACCAAGTCATGC |
|  |  | CYP6B6-R | TCCCCCATTTCACGGAGTTC |
| 118262785 | CYP6B5-like | CYP6B5-F | CGGCATCTATCGGAGCATCA |
|  |  | CYP6B5-R | TAGGACAGCTCGAACAGCAC |
| 118282331 | CYP4d2-like | CYP4d2-F | ACGCCATGGATTCTGTTCGT |
|  |  | CYP4d2-R | ATAGAGGCTCGAAGTGCAGC |
| 118266961 | CYP18a1-like | CYP18a1-F | TGATGTCGACAACGTGAGGG |
|  |  | CYP18a1-R | TACGCGTCTCTTCACATCGG |
| 118279357 | UGT2B7-like | UGT2B7-F | GTGTGTGAGACCCGCTCTTT |
|  |  | UGT2B7-R | TAATCGCTGCCGTCTCAGTC |

**Supplement Figure 1. Liner regression of morality (probit unit) of *Spodoptera frugiperda* and cyproflanilide concentration (Log transformed)**


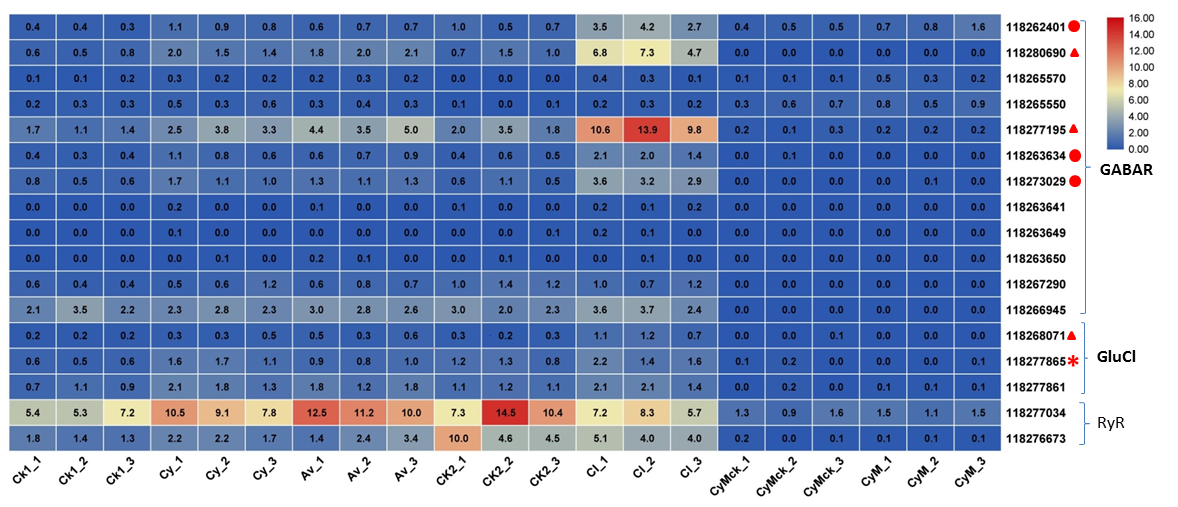


**Supplement Figure 2 Heat map of DEGs on different target proteins based on fragments per kilobase per million (FPKM)**

Cy, cyproflanilide; Av, avermectin; Cl, chlorantraniliprole; ck, control group, CyM, midgut group under cyproflanilide stress.

**
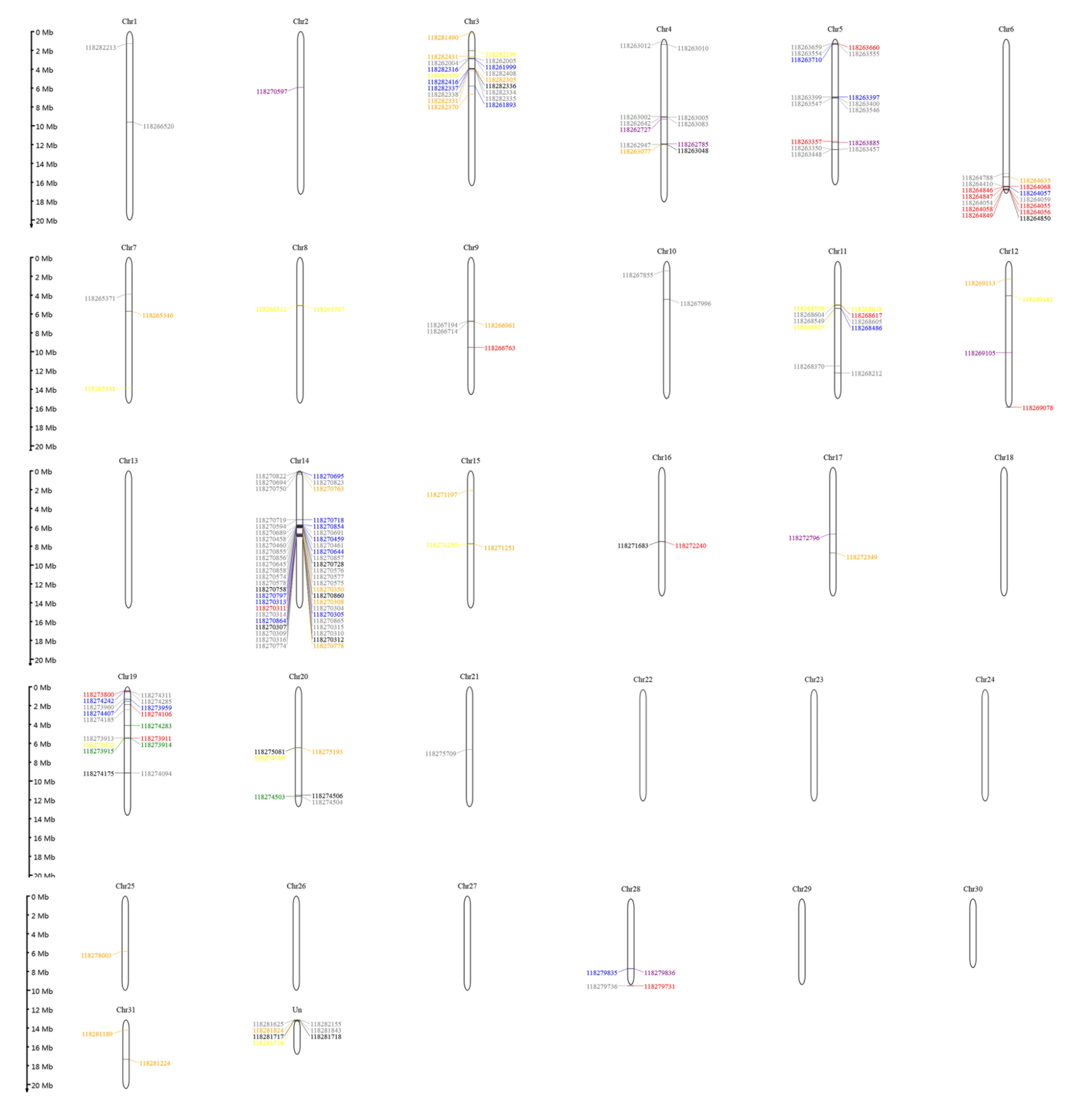
**


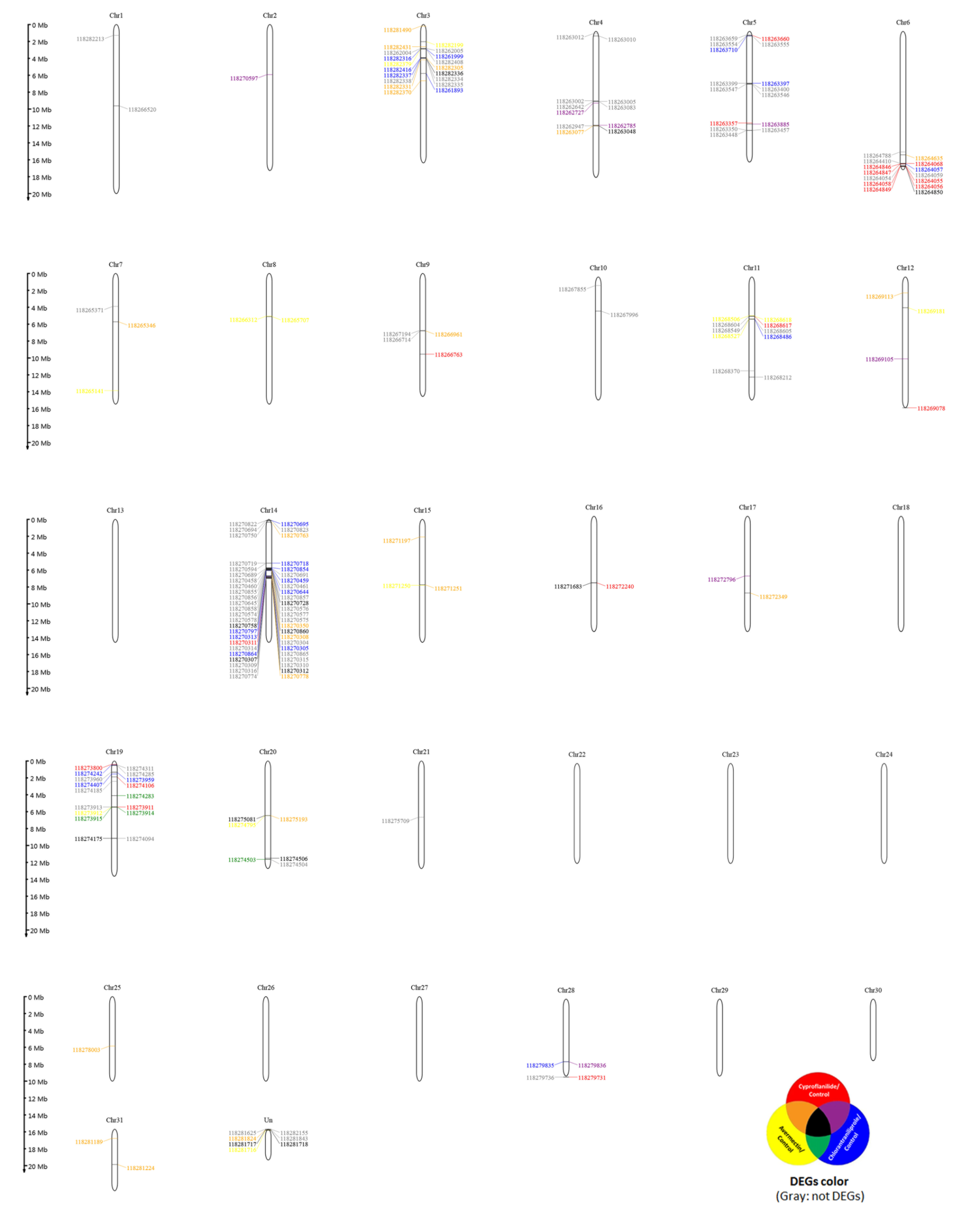


**Supplement Figure 3 P450s scattered among different chromosomes of *Spodoptera frugiperda***

Red color of the accession number indicates P450s that respond specifically to cyproflanilide; yellow color indicates P450s specifically to avermectin; blue color indicates P450s specifically to chlorantraniliprole; orange, purple and green colors in the intersectional region indicates P450s that respond to both two insecticides; black color indicates P450s specifically to all three insecticides; gray color indicates P450s did no response to any of the three insecticides. These loci including 9 gene loci which still to be proved that possibly relates to P450 members (Supplement table 11)


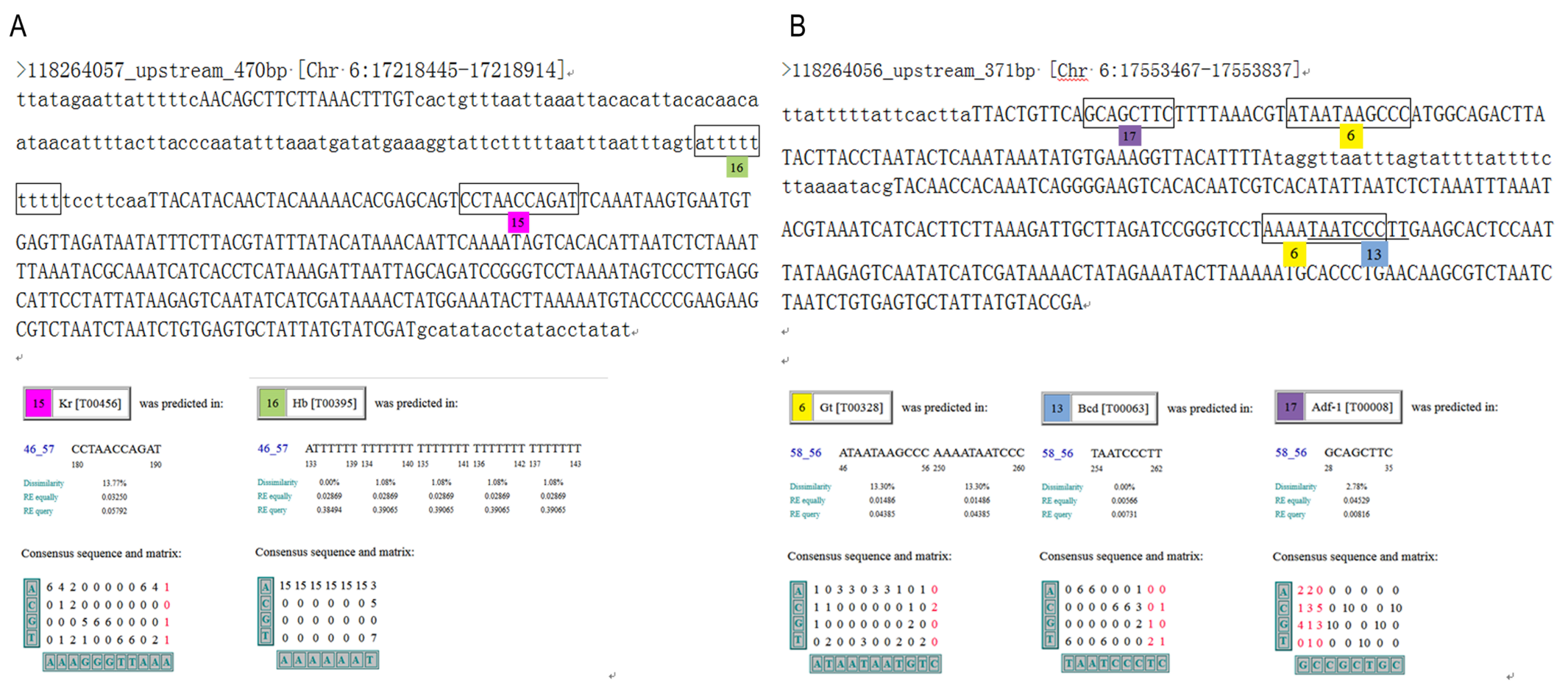


**Supplement Figure 4 TF binding site prediction in the upstream of DGEs.**

A. Upstream of 118264057 (CYP9A26-like); B. Upstream of 118264056(CYP9A26-like).Online software of PROMO website is http://alggen.lsi.upc.es/cgi- bin/promo_v3/promo/promoinit.cgi?dirDB=TF_8.3. Parameters of [SelectSpecies](http://alggen.lsi.upc.es/cgi-bin/promo_v3/promo/promomenu.cgi?dirDB=TF_8.3&Option=0&idCon=166848129400) and SelctFactor were both selected as insecta. Parameters of maximum matrix dissimilarity rate was set as 15%. The 16 common transcription factors shared by 118264057 and 118264056 were: B factor [T00061],Bcd [T00063],Dfd [T00193],Dl [T00196],DSXF [T00955],DSXM [T00956],En [T00253],Eve [T00272],Ftz [T00295],Mad [T04378],Prd [T00699], SGF-3 [T00746],Tll [T00789],Zen-1 [T00917],Zeste [T00918],Zeste [T02100].

**
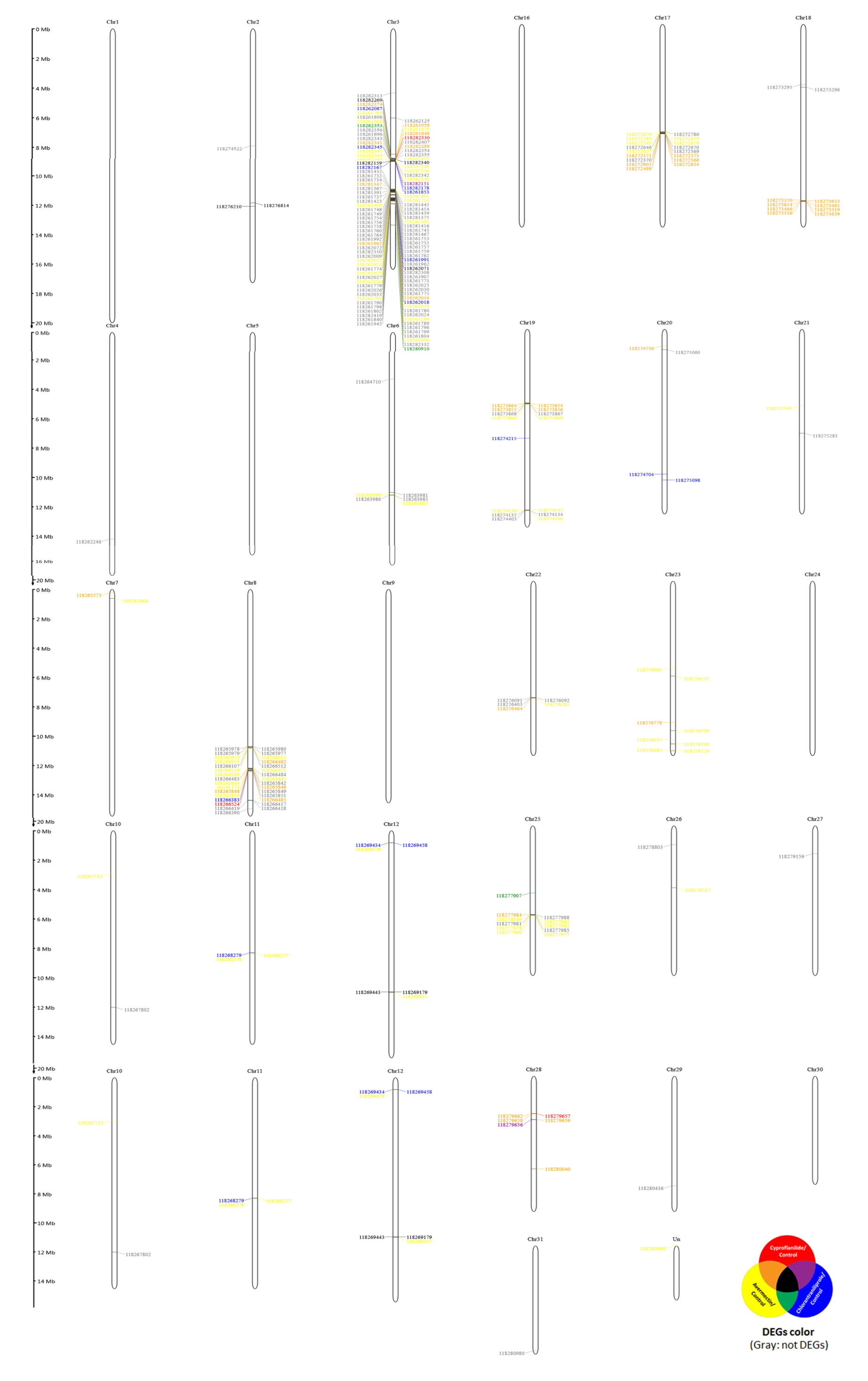
**

**
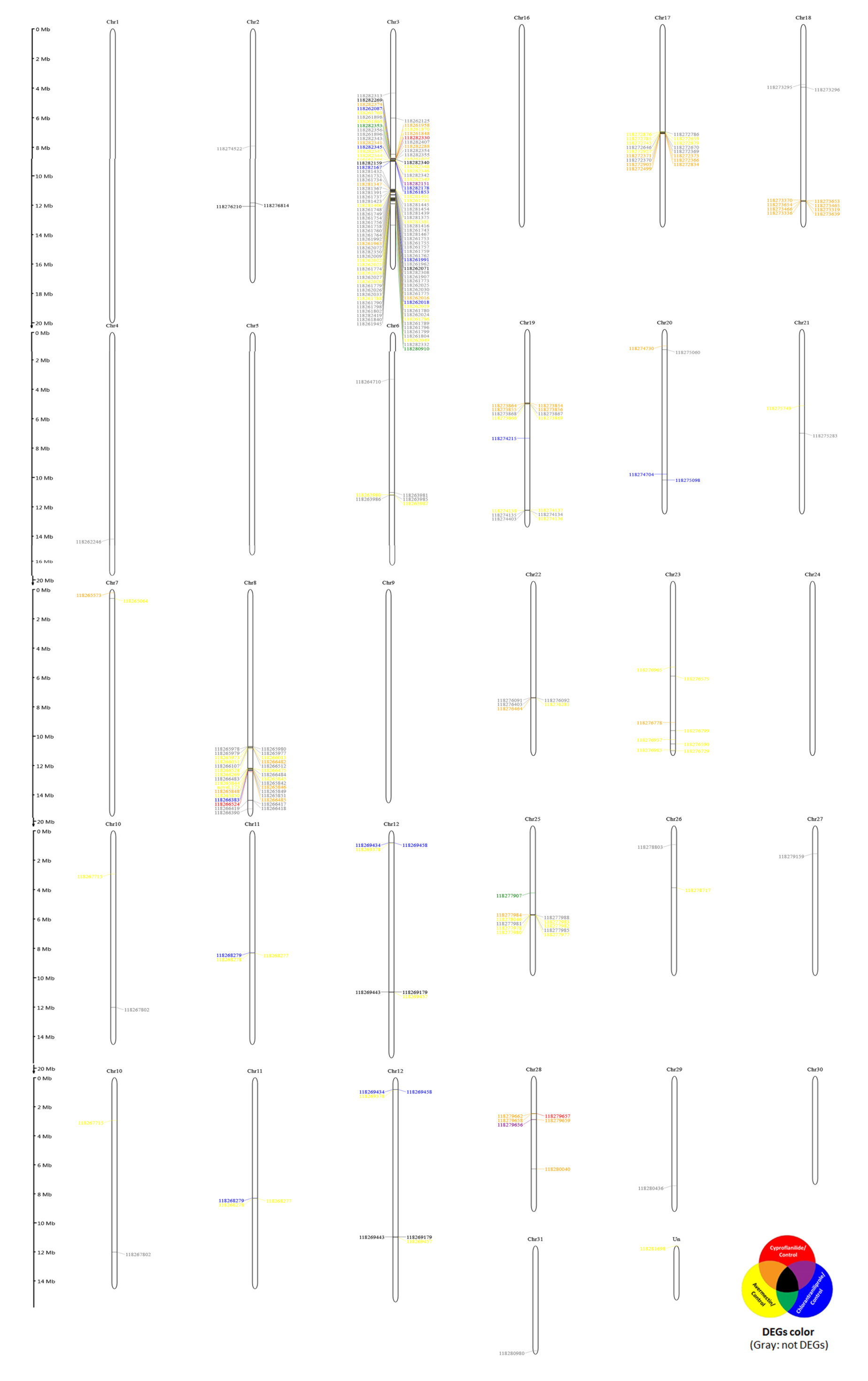
**

**
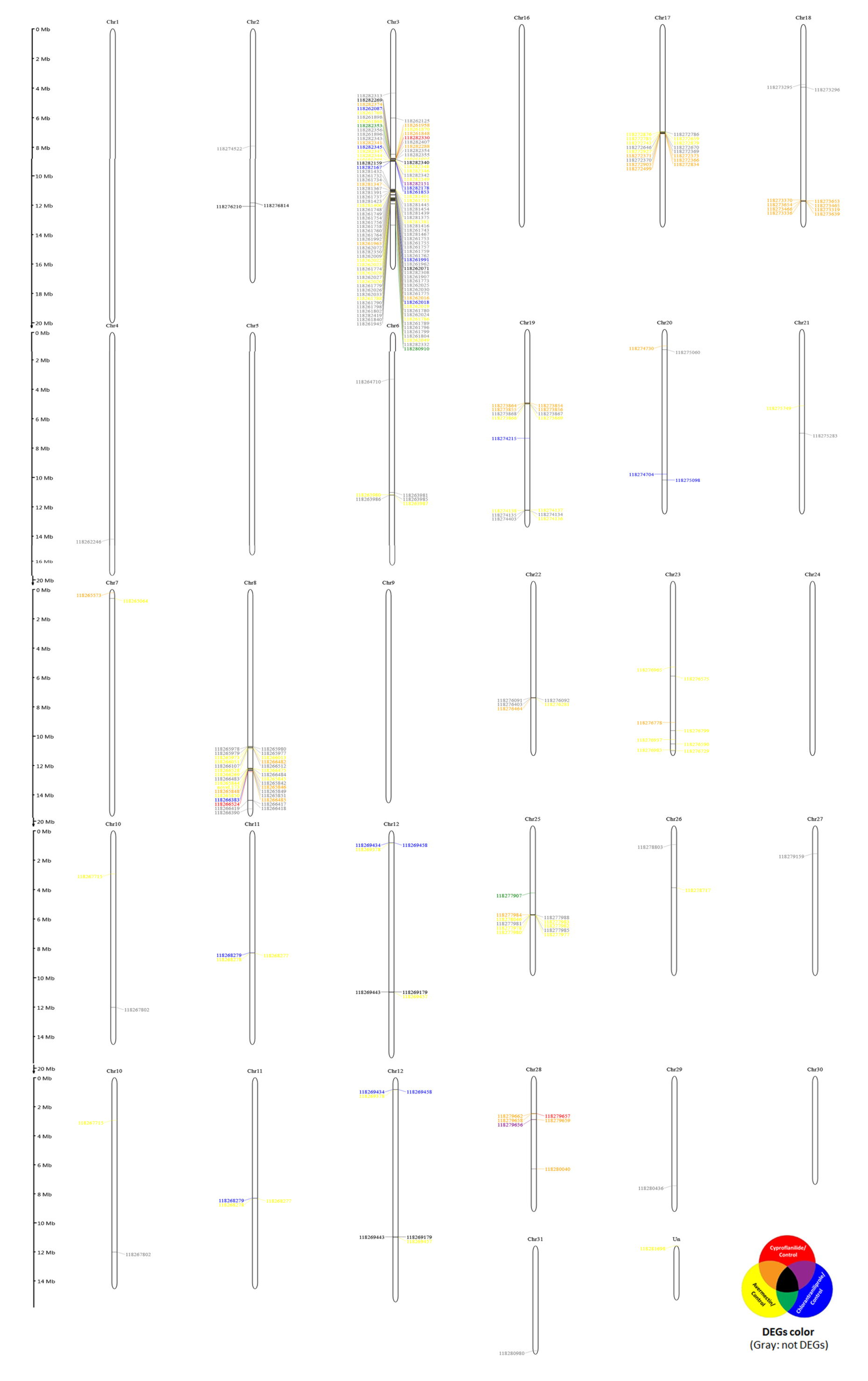
**

**Supplement Figure 5 CPs scattered among different chromosomes of *Spodoptera frugiperda***

Red color of the accession number indicates CPs that respond specifically to cyproflanilide; yellow color indicates CPs specifically to avermectin; blue color indicates CPs specifically to chlorantraniliprole; orange, purple and green colors in the intersectional region indicates CPs that respond to both two insecticides; black color indicates CPs specifically to all three insecticides; gray color indicates CPs did no response to any of the three insecticides.
